# Comparative Proteomic and Morpho-Physiological Analyses of Maize Wild-Type Vp16 and Mutant vp16 Germinating Seed Responses to PEG-Induced Drought Stress

**DOI:** 10.3390/ijms20225586

**Published:** 2019-11-08

**Authors:** Songtao Liu, Tinashe Zenda, Anyi Dong, Yatong Yang, Xinyue Liu, Yafei Wang, Jiao Li, Yongsheng Tao, Huijun Duan

**Affiliations:** 1 Department of Crop Genetics and Breeding, College of Agronomy, Hebei Agricultural University, Baoding 071001, China; m15028293845@163.com (S.L.); tzenda@hebau.edu.cn (T.Z.); 18331220513@163.com (A.D.); 18233230155@163.com (Y.Y.); lxy1696164468@163.com (X.L.); wyf360536991@163.com (Y.W.); LJ337871790219@163.com (J.L.); 2 North China Key Laboratory for Crop Germplasm Resources of the Education Ministry, Hebei Agricultural University, Baoding 071001, China

**Keywords:** proteome profiling, iTRAQ, polyethylene glycol (PEG)-induced drought, energy metabolism, *Zea mays* L.

## Abstract

Drought stress is a major abiotic factor compromising plant cell physiological and molecular events, consequently limiting crop growth and productivity. Maize (*Zea mays* L.) is among the most drought-susceptible food crops. Therefore, understanding the mechanisms underlying drought-stress responses remains critical for crop improvement. To decipher the molecular mechanisms underpinning maize drought tolerance, here, we used a comparative morpho-physiological and proteomics analysis approach to monitor the changes in germinating seeds of two incongruent (drought-sensitive wild-type Vp16 and drought-tolerant mutant vp16) lines exposed to polyethylene-glycol-induced drought stress for seven days. Our physiological analysis showed that the tolerant line mutant vp16 exhibited better osmotic stress endurance owing to its improved reactive oxygen species scavenging competency and robust osmotic adjustment as a result of greater cell water retention and enhanced cell membrane stability. Proteomics analysis identified a total of 1200 proteins to be differentially accumulated under drought stress. These identified proteins were mainly involved in carbohydrate and energy metabolism, histone H2A-mediated epigenetic regulation, protein synthesis, signal transduction, redox homeostasis and stress-response processes; with carbon metabolism, pentose phosphate and glutathione metabolism pathways being prominent under stress conditions. Interestingly, significant congruence (R^2^ = 81.5%) between protein and transcript levels was observed by qRT-PCR validation experiments. Finally, we propose a hypothetical model for maize germinating-seed drought tolerance based on our key findings identified herein. Overall, our study offers insights into the overall mechanisms underpinning drought-stress tolerance and provides essential leads into further functional validation of the identified drought-responsive proteins in maize.

## 1. Introduction

Crop plants, as sessile organisms, are constantly subjected to a plethora of abiotic (drought, heat, cold, salinity, metal toxicity, nutrient deficiency, etc.) and biotic (pathogens, herbivores, nematodes, weeds, etc.) stress factors [1]. All these stress factors impose serious limitations on crop survival, growth and productivity [2,3]. Among the abiotic stresses, drought is the sole factor affecting agricultural crops more than any other, contributing to approximately 70% of potential yield loss globally [4]. The decline in crop yield emanates from drought’s interference with several physiological processes such as growth, photosynthesis and assimilate partitioning [2,5]. In the context of the unequivocal and continuing global climate change, drought events are forecasted to increase in occurrence, duration and intensity, especially in the arid and semi-arid regions of the world [6]. Consequently, the production and productivity of various crops in such regions will be drastically affected [5]. This poses an austere challenge to the food security of the growing world human population, projected to hit above 9 billion people by the year 2050 [7,8].

Maize (*Zea mays* L.), is the third most important cereal crop in the world after rice (*Oryza sativa* L.) and wheat (*Triticum aestivum* L.) [9]. Jointly with rice and wheat, maize provides at least 30% of the food calorie requirements to more than 4.5 billion people worldwide. Additionally, it critically serves its function as a raw material in the food and oil processing, as well as the animal feed and biofuel manufacturing industries [10]. At national level, available data (years 2013–2017) suggests that maize ranks first ahead of rice and wheat in China, in terms of planting area and yield of main food crops (Figure S1). At present, China’s annual maize planting area is approximately 42 million ha (http://data.stats.gov.cn/index.htm). However, about 60% of this area (encompassing the major maize production region of Hebei Province, found in the northern parts of China) is under dry-land farming and located in arid regions, with 20–30% annual maize yield loss experienced attributed to moisture deficit stress [11]. These arid and semi-arid climates are prone to frequent droughts; hence, the negative drought effects have been overwhelmingly observed in such regions where crops are often exposed to soil moisture-deficit stress at the seed germination stage [12]. Unfortunately, maize is susceptible to moisture deficit stress at this stage. Water deficit at this stage impairs germination by inhibiting the imbibition process and reducing seed vigor [13]. This consequently results in germination percentage and rate reduction, poor crop stand establishment and retarded plant growth [2,14], effectually leading to significant yield reductions [15]. Therefore, germination-stage drought stress becomes an area of focus in maize breeding efforts aimed at developing drought-tolerant crop cultivars. An array of studies related to the adverse effects of water deficit on seed germination has been reported in many plant species, including maize [16,17].

In drought-stress response studies [18,19], polyethylene glycol (PEG), a neutral, non-ionic and non-toxic polymer, with high water solubility, has become the widely used osmoticum to mimic decreases in soil water potential. High molecular weight PEG (6000 or above) cannot penetrate the plant cell wall pores, providing conditions closely matching the effect of the reduced matric potentials, thus causing a loss of water from both the protoplast and the cell wall and the collapse of the entire cell, including the wall, with limited metabolic interferences [17]. Thus, PEG solutions have been the most feasible option for simulating drought conditions in short-term experiments.

To cope with drought and other stressors within their environments, plants harbor numerous adaptive response strategies that are activated at physiological, biochemical and molecular levels [3]. Metabolic adjustment (accumulation of osmotically active solutes such as different sugars, betaine and polyamines, as well as amino acids, predominantly proline and glycine) is critical for the maintenance of water status and physiological activity of plant cells, particularly during relatively short-term drought [2]. Osmoprotection via osmotic adjustment and antioxidant scavenging defense system, aided by plant growth regulators, constitute a vital drought-stress response strategy in plants [20]. At the molecular level, plants institute stress-responsive proteins, transcription factors and signaling pathways, among other strategies, to respond to drought stress [21]. Biosynthesis and pronounced accumulation of a broad array of drought-protective proteins, predominantly chaperons, late embryogenesis abundant (LEA), aquaporins and enzymes of antioxidant defense constitute vital molecular responses to drought stress [22]. These molecules confer drought tolerance through protection of cellular contents or via regulation of stress-responsive genes [3].

In recent years, advances in molecular biology techniques have revolutionized comprehensive studies on plant abiotic stress responses [9]. Several -omics strategies such as transcriptomics, genomics, metabolomics and proteomics have become useful in elucidating the intricate drought-stress response mechanisms in crops [15]. Understanding plant drought-stress responses through transcriptomics has been an area of interest in the past two decades, mostly relying on comparative studies of different genetic backgrounds under drought [18]. Several reports [23,24] have focused on studying the plants’ gene expression regulation via transcriptional changes in the regulatory and functional proteins. However, these gene expression regulation studies do not provide much insight into the amount and quality of proteins because the quantitative mRNA data are not always correlated to their corresponding proteins [18]; post-transcriptional and post-translational modifications (phosphorylation, glycosylation, removal of signal peptides, etc.) can result in poor correlations between transcripts and their cognate proteins [25]. Consequently, studying the plant response to abiotic stress at the protein level may offer some useful insights [26]. 

The evolution of the proteomics approach has revolutionized our study of the molecular mechanisms of plant abiotic stress responses. In the previous decade, two-dimensional electrophoresis (2-DE) with peptide mass fingerprinting generated from matrix-assisted laser desorption/ionization (MALDI) mass spectrometry (MS), or peptide sequence tag obtained by MS/MS were applied in seed proteomic studies [27,28]. However, the number of proteins identified in this method is very small [29]. At present, most proteomic studies employ the use of the isobaric tags for relative and absolute quantification (iTRAQ)-based approach. The iTRAQ-based method is a second-generation technique that provides a gel-free shotgun quantitative analysis and uses isobaric reagents to label tryptic peptides and to monitor relative protein and peptide mass tolerance (PMT) abundance changes, as well as allowing for up to eight samples in a single experiment. Thus, the method especially allows for the time-dependent analysis of plant stress-responses or biological replicates in a single experiment [30] and has become increasingly useful in plant stress-response studies. Particularly, the technique has been popular in seed germination studies of a number of plant species, including wheat [31], soybean (*Glycine max* L.) [32] and *Arabidopsis thaliana* L. [27]. Despite all this, however, the interference of germination by drought stress in maize has not been well documented at the proteomic level. Therefore, iTRAQ-mediated analysis can be a powerful tool to examine the molecular changes in specific drought-responsive proteins in maize seeds.

To gain a comprehensive understanding of the molecular mechanisms underpinning maize germinating seeds’ response to PEG-induced drought stress, herein, we have performed high-throughput proteome profiling of the wild-type Vp16 and mutant vp16 lines by iTRAQ-based strategy, after 20% PEG treatment of the germinating seeds. Additionally, comparative morphological and physiological analyses of these maize lines’ responses to drought stress have buttressed the proteomic analysis results in a systems-biology approach. Our findings propel the understanding of the molecular mechanisms of drought-stress response, which could provide solid foundation in the seed germination studies and genetic engineering of new drought-tolerant maize cultivars.

## 2. Results

### 2.1. Morphological Responses of Wild-Type Vp16 and Mutant vp16 Maize Lines to Drought Stress

**Figure 1:**
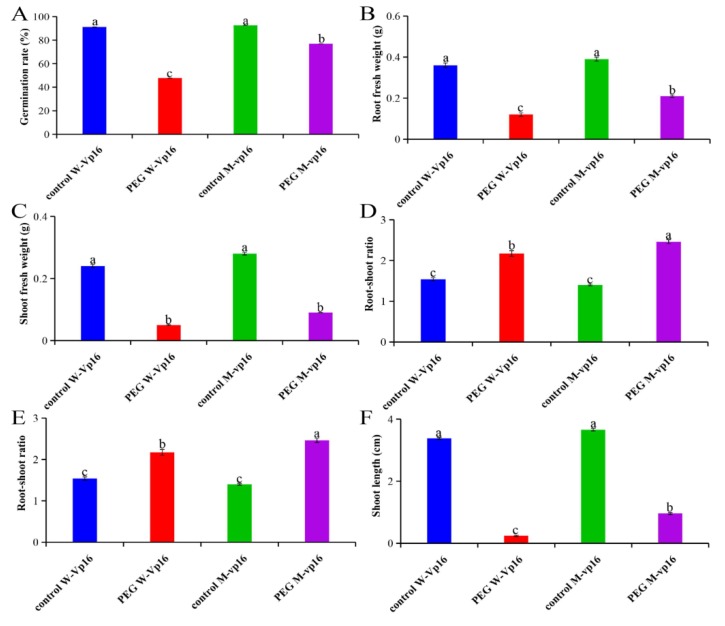
Growth parameters of wild-type Vp16 and its mutant vp16 germinating seeds under well-watered and drought-stress conditions. (**A**–**F**) Germination seeds after a 7-day treatment with or without 20% PEG. (**A**) Germination rate; (**B**) root fresh weight; (**C**) shoot fresh weight; (**D**) root–shoot ratio, root–shoot ratio = root fresh weight/shoot fresh weight; (**E**) root length; (**F**) shoot length. Different letters on error bars represent significant difference at 0.01 level. Data are shown as means ± standard error (SE) (*n* = 5).

Two drought tolerance contrasting materials, sensitive wild-type Vp16 and comparably more tolerant mutant vp16 were used in the present study. Initially, our germination screening results for 20 maize lines showed that mutant vp16 is comparably more drought tolerant than its wild-type Vp16 (Table S1). To confirm these results, here, we measured the two lines’ germinating seeds’ morphological responses to drought stress after seven days PEG treatment under greenhouse environment. With respect to germination rate (GR), root fresh weight (RFW), shoot fresh weight (SFW), root–shoot ratio (RSR), root length (RL) and shoot length (SL), the wild-type Vp16 and mutant vp16 lines did not differ significantly (*p* < 0.01) under control conditions. However, 20% PEG treatment significantly (*p* < 0.01) decreased GR, RFW and SFW in the two lines, with the rate of decline being significantly greater in wild-type Vp16 than in mutant vp16 (Figure 1A–C). Compared to control conditions, RSR significantly increased in both maize lines under drought conditions and was higher (2.46) in mutant vp16 than in wild-type Vp16 (2.17) (Figure 1D). Additionally, RL was significantly (*p* < 0.01) greater (1.38 cm) in mutant line than in wild-type line (0.64 cm) (Figure 1E). Similarly, SL was significantly higher (0.96 cm) in mutant line than its wild-type counterpart (0.24 cm) under PEG treatment conditions (Figure 1F). Overall, our morphological results revealed that the mutant vp16 line had greater growth performance at the germination stage than its wild-type Vp16 under drought-stress conditions.

### 2.2. Summary Output Details of Maize Germinating Seed Proteins Identified by iTRAQ

To identify osmotic stress altered proteins during maize seed germination, total proteins in PEG-6000 treated and untreated maize wild-type Vp16 and its mutant vp16 seeds (three biological replicates each condition) were extracted and analyzed by gel-free iTRAQ labeling and LC-MS/MS methods. We used Mascot software to search MGF files against the Uniprot database. Resultantly, from the twelve protein samples, we detected a total 29,596 peptides (comprising 16,587 unique peptides) and corresponding to 4265 proteins at a false discovery rate of 1%. The molecular weight (MW) distribution of these 4265 identified proteins exhibited broad coverage, ranging from 2 to 200 kDa (Figure S2A). Among them, 121 (3%) weighed <10 kDa, 3583 (84%) weighed 10–70 kDa and 567 (13%) weighed >70 kDa (Figure S2A). The number of peptides making up each protein is distributed in Figure S2B, whereby 2673 (63%) of the total 4265 proteins (Table S2) were detected based on at least two unique peptides (Figure S2B). Additionally, proteins’ sequence coverage was generally below 25% (Figure S2C). Furthermore, Figure S2D shows the length distribution of the peptides defining each protein, whereby most peptides range between 5 to 21 amino acids, with 7–9 and 9–11 amino acids as modal lengths. For the subsequent analysis of differentially abundant proteins (DAPs), we used proteins with at least two unique peptides. 

### 2.3. Analysis of Differentially Abundant Proteins (DAPs) Identified in Different Comparisons

We conducted a comparative proteomic analysis to investigate the changes of protein profiles in germinating seeds of maize wild-type Vp16 (drought-sensitive, W) and its mutant vp16 (drought tolerant, M) under water-sufficient (control, C) and drought-stress (PEG-6000, D) conditions. The following lines by treatment combinations were obtained: WC, WD, MC and MD. Each condition was represented by three biological replicates per line, resulting in 12 samples in total. To evaluate the similarities and differences between these two samples, we conducted a principal component analysis (PCA). The PCA results showed a clear separation between the drought-sensitive wild type and the drought-tolerant mutant lines. Interestingly, the replicates of each treatment clustered together (Figure S3). These results showed that our experiment was reproducible and can be relied upon. 

**Figure 2:**
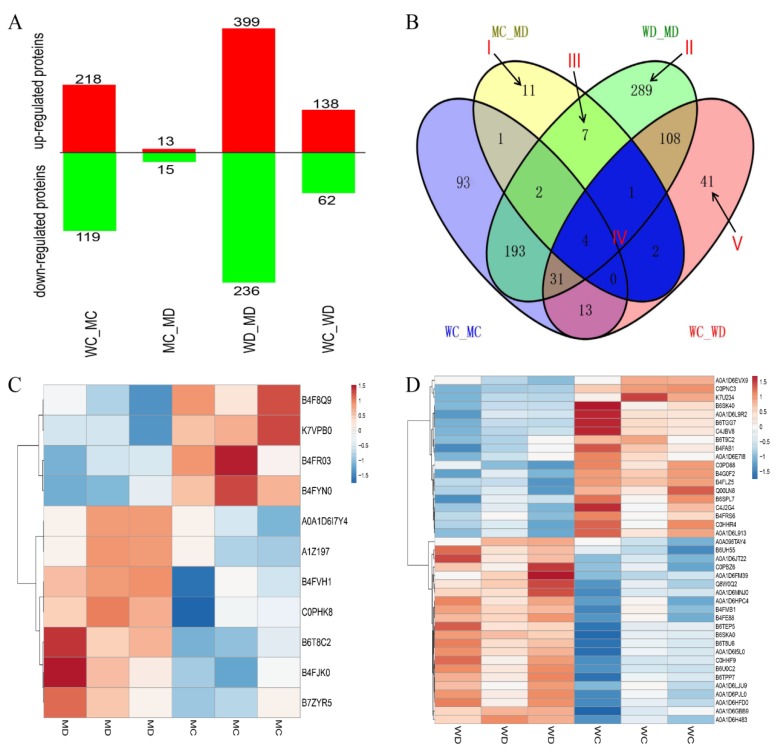
(**A**) Number of differentially abundant proteins (DAPs) expressed in different experimental comparisons. (**B**) Venn diagram analysis of DAPs. The regions labeled Areas I–V identify proteins described under Section 2.3 above. (**C**) Clustering analysis of DAPs unique to MC_MD comparison. (**D**) Clustering analysis of DAPs specific to WC_WD comparison. Each row represents a protein significantly expressed (up-regulated in red and down-regulated in blue); with columns showing three technical replicates for wild-type Vp16 PEG-treated (WD) and wild-type Vp16 under control (WC).

A pairwise comparison of before and after treatments was performed in mutant vp16 (MC_MD) and wild-type Vp16 (WC_WD) individually. Additionally, a comparison research on the drought-related DAPs was carried out between the two lines under PEG-6000 (WD_MD) and water-sufficient (WC-MC) conditions, yielding four experimental comparisons (Figure 2A). Proteins with a fold-change >1.2 (increased) or <0.83 (decreased) were considered to be significantly differentially expressed. Resultantly, we found 1200 DAPs among four groups (Figure 2A). Before drought treatment, we found 337 DAPs, comprising 218 up-regulated and 119 down-regulated (WC_MC in Figure 2A). In the tolerant line mutant vp16, we observed 28 DAPs (comprising 13 up-regulated and 15 down-regulated) before and after drought treatment (MC-MD). In the sensitive line wild-type Vp16, 200 DAPs including 138 up-regulated and 62 down-regulated were identified before and after treatment (WC-WD) (Figure 2A). After drought treatment, a total of 635 DAPs (399 up-regulated and 236 down-regulated) were identified (WD_MD in Figure 2A).

To study the impact of lines or treatments, the distribution of up- and down-regulated DAPs of the four comparison groups were presented in a Venn diagram (Figure 2B). Only seven DAPs were shared among four groups, indicating that these proteins are less affected by environmental and varietal differences. In respect of drought tolerance, some of the group (Venn) combinations are more vital than others. Area I represents specific DAPs of MC_MD, that is, the specific drought-responsive DAPs of the drought-tolerant line mutant vp16. Of these 11 DAPs, 7 were up-regulated and 4 were down-regulated (Table 1). Area II represents the 289 drought-responsive DAPs unique to WD_MD (Table S3); of which 151 were up-regulated and 138 down-regulated, that is, specific DAPs shared between the drought-sensitive and drought-tolerant lines after drought treatment. Area III represents the seven specifically shared DAPs between MC_MD and WD_MD, that is, drought-responsive DAPs of the tolerant line that were also differentially expressed between the tolerant and sensitive lines after drought treatment. Of these seven DAPs, one DAP was up-regulated in both groups; three DAPs were up-regulated and four DAPs down-regulated in WD_MD, whereas the expression of these proteins in group MC_MD showed an opposite trend (Table S4). Area IV represents the seven DAPs shared by MC_MD and WC_WD, that is, the common (overlapping) drought-responsive DAPs within line. Of these seven common drought-responsive DAPs, five were down-regulated and one was up-regulated in both the tolerant mutant vp16 line and sensitive wild-type Vp16 line; whereas one DAP was up-regulated in wild-type Vp16 but down-regulated in mutant vp16 (Table S5). For comparative analysis, Table S6 shows the 41 drought-responsive DAPs unique to WC_WD (labeled V in Figure 2B), of which 22 were up-regulated and 19 down-regulated. 

To further understand the protein expression between drought and well-watered conditions, we performed the hierarchical clustering analysis of the identified DAPs (Figure 2C,D). The DAP in different replicates of the same line and treatment condition showed similar expression patterns. However, the same DAP showed different expression patterns in different treatment condition (Figure 2C,D). 

### 2.4. Gene Ontology (GO) Classification and Analysis of Drought-Responsive DAPs

To further characterize the DAPs identified from the pairwise comparisons, we performed gene ontology (GO) annotation to assign level 2 GO terms to the DAPs using Blast2GO web-based program (https://www.blast2go.com/). Our analysis results showed that a larger number of DAPs in the biological process (BP) and molecular functions (MF) categories were shared among the WC_WD, WD_MD and MC_MD experimental comparisons under stress conditions. These include cellular process (GO:0009987), metabolic process (GO:0008152), response to stimulus (GO:0050896), developmental process (GO:0032502), biological regulation (GO:0065007) and multicellular organismal process (GO:0032501) in the BP category; whereas binding (GO:0005488), catalytic activity (GO:0003824), structural molecule activity (GO:0005198) and transporter activity (GO:0005215) were prominent in the MF category (Figure S4A–C). 

**Figure 3:**
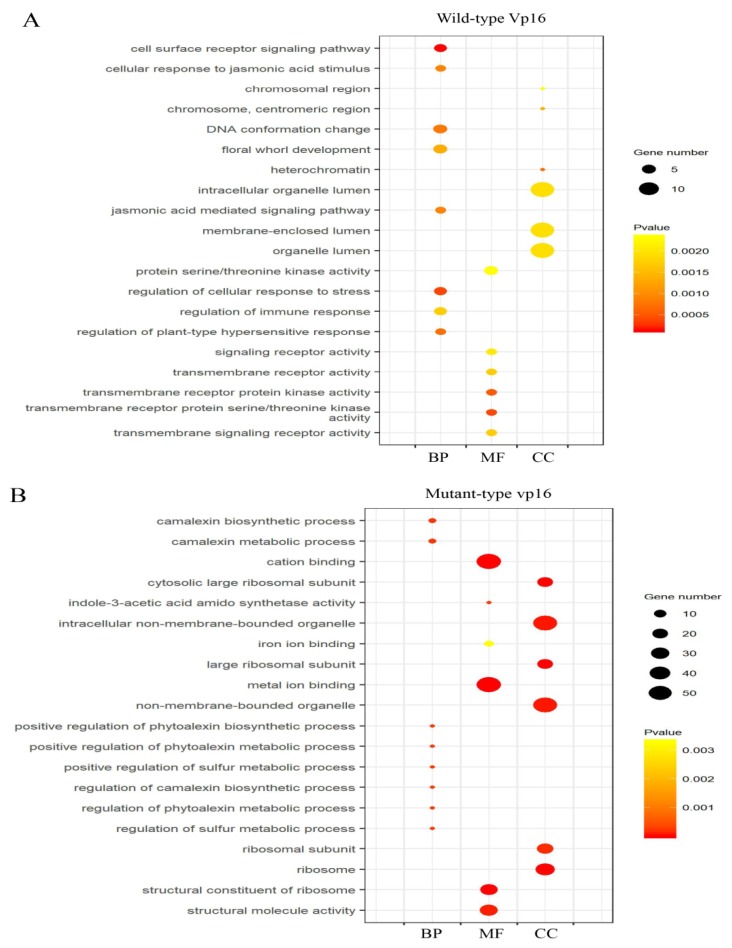
GO functional classification of drought-responsive proteins. Most significantly enriched GO terms (top 20) under PEG treatment in (**A**) wild-type Vp16; and (**B**) mutant vp16. The color and size of the dot reflect the level of enrichment and the number of genes enriched in each GO term, respectively. BP, biological process; MF, molecular function; CC, cellular components functional categories.

However, further analysis of the enriched top 20 GO terms by GO enrichment analysis (*p*-value <0.05) revealed clear differences in the GO terms between the two lines under drought stress (Figure 3A,B). 

In osmotic-stressed wild-type Vp16 seeds, camalexin metabolic process (GO:0052317), positive regulation of camalexin biosynthetic process (GO:1901183) and regulation of sulfur metabolic process (GO:0042762) were dominant under the BP category, whereas GO terms metal ion binding (GO:0046872), cation binding (GO:0043169) and structural molecule activity (GO:0005198) were prominent in the MF category (Figure 3A). Contrastingly, in osmotic-stressed mutant vp16 seeds, cell surface receptor signaling pathway (GO:0007166), regulation of cellular response to stress (GO:0080135) and DNA conformation change (GO:0071103) were dominant terms in the BP category. In the MF category, transmembrane receptor protein (GO:0004675) and transmembrane receptor protein kinase activity (GO:0019199) were most apparent (Figure 3B). We assumed these differences in the most significantly enriched GO terms to contribute to the two maize lines’ contrasting drought tolerance, hence arousing our interest for further discussion.

### 2.5. KEGG Pathway Enrichment Analysis of DAPs

The KEGG database (available online: https://www.genome.jp/kegg/; accessed on 8 February 2019) was used to assign and perform pathway enrichment analysis of the DAPs from the WC_WD, MC_MD and WD_MD experimental comparisons. Consequently, DAPs from the WC_WD, MC_MD and WD_MD comparisons were assigned to 58, 27 and 91 KEGG metabolic pathways respectively. The top 20 KEGG pathways for each group are provided in Figure S5. Our analysis showed that ribosome, glutathione metabolism and mitogen-activated protein kinase (MAPK) signaling pathways were commonly enriched in both lines after PEG treatment (Figure S5A,B). However, linoleic acid metabolism and benzoxazinoid biosynthesis pathways were specifically enriched in wild-type Vp16, whereas carbon metabolism and glycolysis/glycogenesis pathways were uniquely enriched in mutant vp16 in response to drought stress (Figure S5B). Meanwhile, in WD_MD comparison, several pathways responded to drought stress including ribosome, pentose phosphatase, glutathione metabolism, glycolysis, and starch and sucrose metabolism pathways, among others (Figure S5C). 

**Figure 4:**
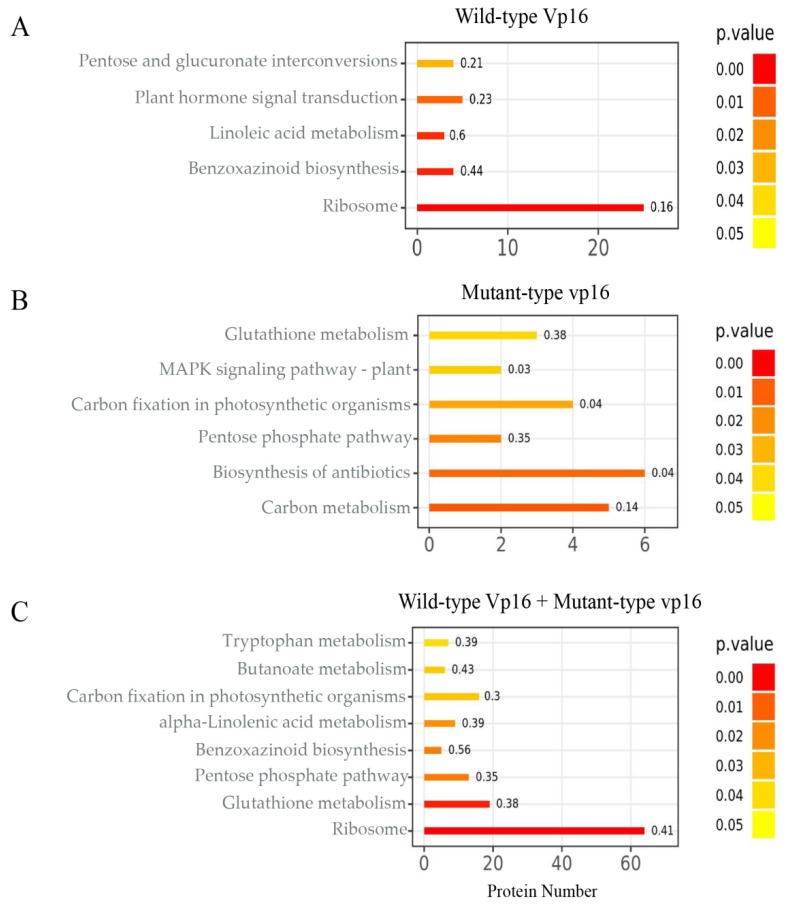
KEGG pathway enrichment analysis of DAPs. Most significantly enriched pathways under PEG treatment in (**A**) wild-type Vp16; (**B**) mutant vp16; and (**C**) WD_MD comparison. The color gradient (orange to red) corresponds to the level of significance (lower to higher; *p*-value <0.05; Student’s *t*-test) of enrichment of the corresponding KEGG pathway. The number on each bar graph indicates enrichment factor (rich factor ≤1).

We used the hypergeometric test (*p*-value <0.05) to explore the KEGG pathways that were significantly altered by drought stress. Resultantly, ribosome, benzoxazinoid biosynthesis, linoleic acid metabolism and plant hormone signal transduction pathways were the most significantly enriched in sensitive line wild-type Vp16 (Figure 4A). Contrastingly, carbon metabolism and pentose phosphate pathways were the most significantly enriched in tolerant line mutant vp16 (Figure 4B). In the WD_MD comparison, ribosome, glutathione metabolism and pentose phosphate pathways were the most significantly enriched (Figure 4C). For graphical view of the most significantly enriched metabolic pathways in these maize lines, we refer you to Figure S6. We suggest that the diverse drought-stress responses exhibited by the two lines, in relation to the most significantly enriched pathways, are an entry point to dissect the drought tolerance divergence of these two lines. 

### 2.6. Expression Levels of Genes Encoding DAPs in Response to Drought Stress

The protein expression levels obtained by iTRAQ sequencing were confirmed by quantitative real-time polymerase chain reaction (qRT-PCR). We randomly selected twenty-eight representative genes (Table S7) for qRT-PCR analysis based on the following criterion: highly differentially accumulated in response to PEG-induced drought stress and identified as key proteins according to the GO and KEGG enrichment analyses. Our results showed that there was high consistence (correlation coefficient, R^2^, of 81.51%) between the transcriptional patterns and levels and iTRAQ sequencing data of these twenty-eight representative genes (Figure S7; Table S8). In a nutshell, the qRT-PCR analysis results confirmed our iTRAQ analysis-based findings.

### 2.7. Physiological Responses of Wild-Type Vp16 and Mutant vp16 Maize Lines to Drought Stress

**Figure 5:**
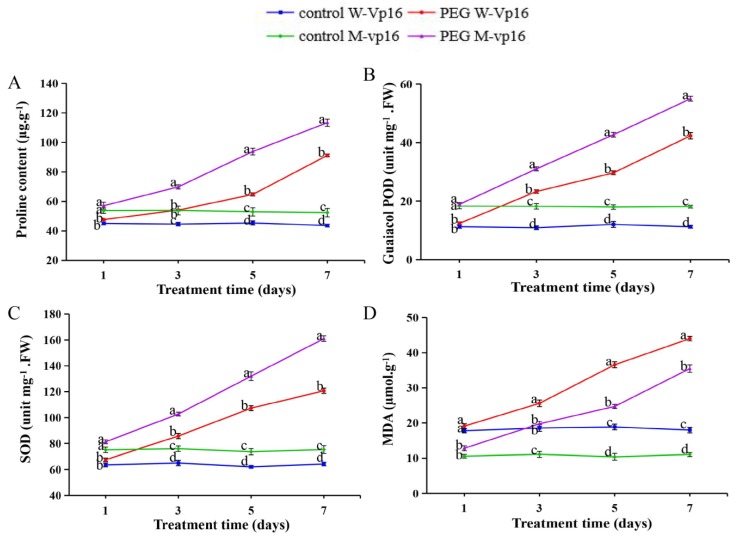
Physiological parameters of wild-type Vp16 and its mutant vp16 germinating seeds after a 7-day treatment with or without 20% PEG. (**A**) Proline content; (**B**) guaiacol peroxidase (POD) activity; (**C**) superoxide dismutase (SOD) activity; (**D**) malondialdehyde (MDA) content. Data are presented as means ± SE (*n* = 3). Different letters above line graphs show significant difference (*p* ≤ 0.01) among treatments at a given treatment time point.

To evaluate the two maize lines’ physiological responses to PEG-induced drought stress, we determined some physiological parameters, namely, proline (Pro) content, guaiacol peroxidase (POD) and superoxide dismutase (SOD) enzyme activities and malondialdehyde (MDA) content in the germinating seeds. Resultantly, Pro content significantly (*p* < 0.01) increased in both wild-type and mutant lines under drought-stress conditions. Interestingly, Pro content was generally higher in mutant vp16 than in wild-type Vp16 (Figure 5A). The POD and SOD enzyme activities showed a similar increasing trend under osmotic stress conditions (Figure 5B,C), indicating that certain drought-stress intensity could result in increased activity of antioxidant enzymes in maize germination seeds. Meanwhile, MDA content was significantly (*p* < 0.001) higher in wild-type Vp16 than in mutant vp16 under both non-stressed and stressed conditions. Notably, PEG-induced drought stress significantly increased MDA content in both lines, with much greater increase in wild-type Vp16 than in mutant vp16 (Figure 5D). Overall, these findings revealed that under PEG treatment, the Pro content, SOD and POD activities were higher in the mutant line, whilst MDA content was greater in the wild-type line. 

## 3. Discussion

To clarify the molecular mechanisms underpinning germinating maize seed drought-stress tolerance, here, we used iTRAQ proteomics-based method to conduct a comparative analysis of two (sensitive wild-type Vp16 and tolerant mutant vp16) lines considered to significantly vary with respect to their physiological drought-stress responses. Additionally, we performed morphological, physiological and qRT-PCR analyses to buttress the proteomics findings. Our results lay bare some insights into the molecular mechanisms associated with maize germinating seeds drought tolerance.

### 3.1. Wild-Type Vp16 and Mutant vp16 Lines Showed Significant Variation in Their Morphological and Physiological Drought-Stress Responses

In maize, as in other crop species, genotypic differences in drought-stress responses, with respect to phenotypic and physiological traits, have been identified [9,19,24,33]. Here, our experimental observations on both morphological and physiological parameters showed that maize wild-type Vp16 and mutant vp16 lines performed differently under osmotic stress conditions (Figure 1 and Figure 5). The growth rate index under water deficit conditions can reflect the germination ability in different plant species. Drought-stress-induced reduction in growth rate at the earliest stage of plants’ growth cycle has been reported [34,35]. Here, the GR of both wild-type and mutant maize lines declined obviously under osmotic conditions (Figure 1A). However, other growth traits, including RFW, SFW, RSR, RL and SL, were all less affected by drought stress in mutant vp16 than in wild-type Vp16 (Figure 1B–F). A direct consequence of water deficit is cellular dehydration, leading to reduced cell expansion [2]. Drought stress typically reduces shoot growth at the early seedling growth stage. On the other hand, root elongation is less sensitive to growth inhibition than shoot elongation under water-limited conditions [36]. Therefore, the maintenance of root growth under water deficit conditions plays a key role in water uptake. Consequently, this increases the possibilities of maize survival under such stressful conditions.

Plants have evolved an elaborate antioxidant defense system for protecting the cell against reactive oxygen species (ROS)-triggered oxidative damage [37]. The SOD enzyme constitutes the first line of defense via superoxide radicals’ detoxification [38]. The POD is important as a hydrogen peroxide (H_2_O_2_) scavenging enzyme [39]. The balances among the SOD, POD and catalase activities are pivotal to maintaining the H_2_O_2_ homeostasis in plants [40]. In the current study, the mutant vp16 line accumulated greater POD and SOD activities than wild-type Vp16 under drought-stress conditions (Figure 5B,C). This may have contributed to better ROS quenching capability of mutant vp16 as compared to its wild-type counterpart. Additionally, our investigation showed that mutant vp16 accumulated prominently greater proline content than wild-type Vp16 seeds under drought-stress conditions (Figure 5A). Increased proline content in the cells lowers cell water potential, thereby increasing cell water retention in response to water stress [40]. Consequently, greater accumulation of proline in mutant vp16 under drought-stress conditions may have contributed to its superior drought tolerance as compared to its wild-type counterpart. 

MDA is generated by lipid peroxidation and the change in MDA content is reflective of the extent of cell membrane damage [19]. In the present study, MDA content was significantly higher in wild-type Vp16 than in mutant vp16 under drought-stress conditions (Figure 5D). The higher membrane stability index may also have imparted improved drought-stress tolerance in mutant vp16 [41]. Taken collectively, our results showed that the two maize lines varied considerably in their morphological and physiological drought-stress responses, with mutant vp16 being comparably more tolerant than wild-type Vp16, probably due to its improved ROS scavenging competency, robust osmotic adjustment as a result of greater cell water retention and enhanced cell membrane stability index. 

### 3.2. Carbohydrate/Energy Metabolism-Related Proteins under Drought

Altering a network of events associated with carbohydrate (CHO) and energy metabolism under drought stress can be exploited to improve plants’ tolerance [24]. Previously, in studies involving wheat [42] and rice [43], proteins associated with CHO and energy metabolism were identified under water-deficient conditions. As anticipated, in this report, a fraction of DAPs related to carbohydrate and energy metabolism was observed to respond to drought stress (Table 1). Malate dehydrogenase (MDH) is a key enzyme in glyoxylate and dicarboxylate metabolism processes. As a key component of the tricarboxylic acid cycle (TCA), MDH reversibly catalyzes the oxidation of malate to oxaloacetate offering energy for the cell [44]. The increased abundance of MDH (B4FVH1) in tolerant line mutant vp16 facilitated cell energy homeostasis by reducing the equivalents between subcellular compartments in cooperation with the membrane-bound dicarboxylate transporters. Increased abundance of MDH in response to water-limited conditions has also been reported in barley [45]. 

Additionally, our analysis of the DAPs common between the sensitive wild-type line Vp16 and tolerant mutant line vp16 showed that alpha-amylase (B4G231) was the only DAP that was up-regulated in both lines in response to drought stress (Table S5). Alpha-amylase is involved in starch and sucrose metabolism. Particularly, alpha-amylase is the main enzyme for starch degradation [46]. We can herein infer that whilst plant accumulation of osmotic substances such as soluble sugar and proline can maintain osmotic regulation of cells [47], the degradation of starch into soluble sugar can improve the concentration of osmotic regulatory substances and provide abundant energy for normal growth of germinating seeds [48]. Furthermore, in the current study, we identified several uncharacterized proteins that responded to drought stress including three (A1Z197, B7ZYR5 and C0PHK8) that were specifically up-regulated in tolerant line mutant vp16. Interestingly, these were among the topmost expressed proteins in mutant vp16 (Table 1). We speculate that these predicted proteins have critical functions in the regulatory network for drought-stress tolerance in maize germinating seeds and may therefore need further characterization.

### 3.3. Histone H2A Are the Main Histone Proteins Responsive to Drought

Epigenetic regulation is a key mechanism that is involved in a wide range of biological phenomena, including genome stability, gene expression and developmental programming [49]. Particularly, chromatin-regulation-mediated epigenetic modulation processes, via histone modification, can be dynamically altered to maintain gene activities under stress conditions. In general, a basic core histone octamer for nucleosomes is built upon histones H2A, H2B, H3 and H4. At their tails (N-terminal regions), histones are enriched with basic amino acid residues such as lysine and arginine [50]. As these basic residues in histone tails are covalently modified by post-translational modifications (PTMs; acetylation, phosphorylation, methylation, or ubiquitination), the activity of the genes that are wrapped around the core histones are altered. The transcriptional responsiveness of stress-upregulated genes is therefore correlated with changes in histone modification [48,49]. In the current study, histone 2A proteins (B6T8C2 and B4FJK0) were uniquely expressed in tolerant line mutant vp16 and were the topmost up-regulated proteins (Table 1). Our results may suggest that these histone proteins play a critical role in drought-stress response, by providing the sites for covalent modification of the amino acid residues. However, it is not clear from our results which histone modification mechanism was prominent. Since histone modification can have different effects depending on the residue modified and the type of modification [50], further downstream analysis of the identified histone proteins will ascertain which among the PTMs is most essential in germinating seeds’ osmotic stress response. 

### 3.4. Protein Synthesis and Proteolysis-Related Proteins under Drought Stress

**Figure 6:**
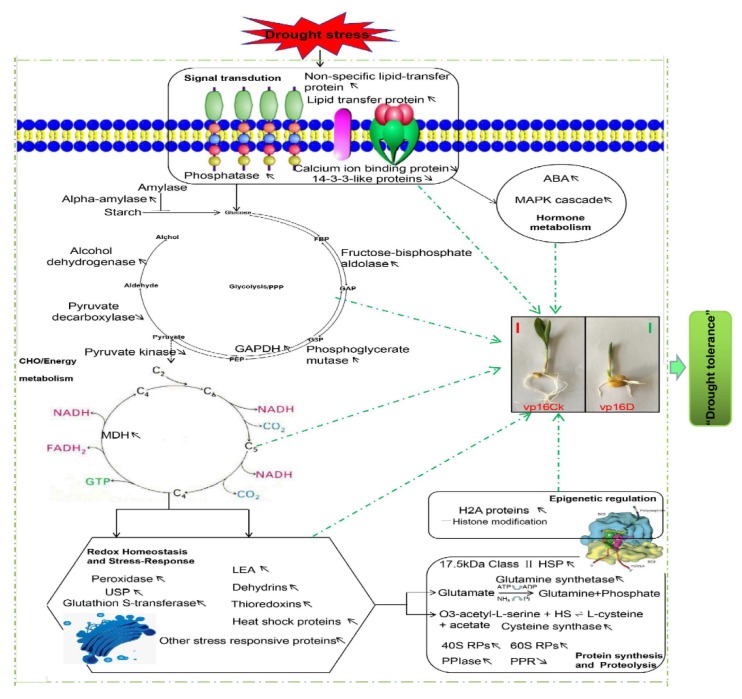
Proposed model of drought-stress tolerance in maize germinating seeds, based on our key findings of this study. Up- or down-regulation of proteins in both wild-type Vp16 and its mutant vp16 are marked by upward and downward pointing arrows, respectively. Key to abbreviations: ABA, abscisic acid; MAPK, mitogen-activated protein kinases; GADPH, glyceraldehyde-3-phosphate dehydrogenase; MDH, malate dehydrogenase; SDH, succinate dehydrogenase; LEA, late embryogenesis abundant proteins; 40S RPs, 40S ribosomal proteins; 60S RPs, 60S ribosomal proteins; PPIase, peptidylprolyl isomerase; PPR, pentatricopeptide repeat protein; USP, universal stress protein.

Under drought-stress conditions, the up-regulation of proteins involved in protein synthesis contributes to restoration of damaged proteins and synthesis of stress-defense proteins [51]. Additionally, maintenance of proper protein functional confirmations, accurate protein turn-over and prevention of protein misfolding are critical for cell survival under such adverse conditions [52]. In the current study, a set of protein synthesis-related DAPs were significantly up-regulated in response to drought stress (Table S3; Figure 6). In particular, twenty-four ribosomal (60S and 40S) proteins (RPs), eight amino acid (mostly cysteine and methionine) metabolism-related proteins and cysteine synthases were significantly up-regulated in the WD_MD experimental comparison (Table S3). The 60S and 40S RPs are vital cogs in the stress-defense protein biosynthesis machinery. Previously, RPs were identified to be significantly up-regulated in response to drought stress in wheat [53]. Cysteine synthase is a key enzyme for mediating abiotic stress tolerance through its catalysis in the production of antioxidants and metal chelators, including glutathione and phytochelatin [18]. The significant up-regulation of cysteine synthase was previously observed in the tolerant varieties in rice [18] and roots of wheat [54] during stress, suggesting its essential role in protein biogenesis in response to drought stress. 

Moreover, peptidylprolyl isomerase (PPIase) enzyme (A0A1D6I7Y4) was specifically up-regulated in mutant vp16 under drought stress (Table 1). The GO molecular function of PPIase is peptidyl-prolyl cis-trans isomerase activity (https://www.uniprot.org/uniprot/A0A1D6I7Y4). In *Arabidopsis*, PPIase was reported to catalyze cis–trans isomerization of the peptidyl–prolyl bond, a rate-limiting step in protein folding [55]. Here, we may also speculate that PPIase plays a crucial role in modulating protein folding in cells of drought-stressed germinating seeds [56]. Increased abundance of PPIase in response to drought stress has been reported in tolerant sorghum line [57]. Taken together, our results revealed that protein synthesis and proteolysis-related DAPs, crucial in re-establishing normal protein structures and cellular homeostasis, were abundantly expressed to help the germinating seeds cope with osmotic stress in the tolerant line mutant vp16. 

### 3.5. Stress Signal Transduction and Lipid-Metabolism-Related Proteins

Protein kinases and phosphates are central to the stress signal transduction machinery that facilitates protein phosphorylation and dephosphorylation, respectively [58,59]. Particularly, the MAPKs and calcium-dependent protein kinases (CDPKs) are vitally involved in plant abiotic stress responses [60]. Here, several non-specific lipid transfer proteins (nsLTPs, Table S3; Figure 6) and lipid-metabolism-related protein (B6T2T4 in Table S4) were up-regulated in response to drought stress. The nsLTPs have been implicated in numerous biological functions, including long-distance stress signal transduction, plant pathogen defenses and hydrophobic layer formation on the surfaces of plant organs [61,62]. Here, we speculate that the enhancement of lipid-metabolism-related proteins could effectually contribute to increased stress signaling and cell water retention, thereby allowing for normal growth under stress conditions. Further, nsLTPs improve the abiotic–biotic cross-tolerance mechanisms in stressed plants [63]. However, the down-regulation of the calcium ion binding protein (B4F8Q9, Table 1; Figure 6) and MAPK-related gene (Zm00001d045310, Table S5) may imply the complexity of the stress signaling network as components interact to effect certain drought-stress responses.

### 3.6. Cellular Redox Homeostasis and Stress-Related Proteins under Drought

To prevent the oxidative damage of cellular components arising from abiotic stress-induced ROS, plants have evolved intricate arrays of enzymatic and non-enzymatic mechanisms, essential in redox homeostasis for maintenance of the steady-state level of ROS [2,64,65]. Additionally, plants alter their metabolism processes and institute stress-related proteins (such as HSPs, LEA proteins, chaperons and water proteins) for an effective re-establishment of the cellular redox balance [3,66]. Here, the abundance of several protective and stress-related proteins, such as antioxidant enzymes, dehydrins (DHNs) and chaperones, was altered by drought stress. We observed four peroxidases, seven glutathione S-transferases (GSTs), 17.5 kDa class II HSP (B6T339), two thioredoxins (THXs) and three USP (universal stress protein) family proteins to be significantly up-regulated in the MD_WD experimental comparison in response to drought stress (Table S3). These results are consistent with our physiological analysis results that POD enzyme activity was significantly increased, alongside proline content, in response to drought stress (Figure 5A,B). They also resonate well with previous findings [44]. 

Peroxidases, mostly located in the vacuole, quench superoxide, hydroxyl radicals and singlet oxygen in cytosols, chloroplasts and mitochondria [67]. Previously, Faghani et al. [68] realized these proteins to be significantly up-regulated in wheat response to drought stress. Additionally, previous reports have revealed the up-regulated expression of peroxidases under drought stress, including those in maize [69]. GSTs play a role in conjugating tripeptide glutathione to a great number of exogenous and exogenous hydrophobic electrophiles [70]. In line with our research, GSTs were reported to have an increased accumulation in rice responding to osmotic stress [71]. Further, transgenic GST tobacco plants increased their resistance to drought stress [72]. 

Chaperones are low-molecular-weight HSPs that play a vital role in regulating proper folding or unfolding of proteins, as well as prevention of unwanted aggregation under abiotic stress conditions [73]. Here, the increased expression of small HSP (sHSP) (B6T339) indicated that higher accumulation of chaperons may assist maize germinating seeds’ cells to cope with drought stress (Figure 6). The up-regulation of sHSPs has been previously reported in response to drought stress in wheat [68]. The THXs are a critical component of the chloroplastic THX redox system [74]. DHNs are a distinct and ubiquitous group of LEAs that are abscisic acid (ABA)-responsive, suggesting that their expression can be directly increased by the phytohormone [75]. The critical roles played by DHNs in plant abiotic stress responses have been extensively acknowledged [76]. Taken together, this discussion may indicate that these identified stress-related proteins play key roles in drought tolerance in maize germinating seeds by promoting the maintenance of normal cellular redox homeostasis under drought conditions. 

### 3.7. Most Significantly Enriched Metabolic Pathways in Response to Drought

The germinating seeds’ proteomes revealed changes in major metabolic pathways (Figure 4). We paid much attention to the tolerant line mutant vp16 and WD_MD exclusive pathways and observed that carbon metabolism, pentose phosphate and glutathione metabolism pathways were the most significantly influenced in response to osmotic stress (Figure 4B,C). MDH was up-regulated in tolerant line mutant vp16, but not in sensitive line wild-type Vp16 in response to drought stress (Table 1). MDH is a key enzyme of the TCA cycle, which reversibly catalyzes the oxidation of malate to oxaloacetate. This reaction is accompanied by the production of NADPH, which is a reducing agent for various synthetic reactions in living systems [68]. Here, we suggest that mutant vp16 cells produced more NADPH under PEG treatment than wild-type Vp16 cells, consequently helping the germinating seeds to endure PEG-induced drought stress [44]. 

For the pentose phosphate pathway, fructose-bisphosphate aldolase, 6-phosphogluconate dehydrogenase and phosphoglycerate mutase were up-regulated in the tolerant line mutant vp16 under drought-stress condition, leading to abundant production of NADPH, an essential reducing agent for numerous synthetic reactions in the cells (Figure 6). Pentose phosphate pathway has been significantly enriched in response to various abiotic stresses [51,77]. Additionally, the NADPH resulting from the pentose phosphate pathway is the cofactor essential for the conversion of oxidized glutathione to reduced glutathione [78]. Glutathione is critical for redox homeostasis maintenance as one of the major antioxidants involved in ROS elimination [51,79]. Here, we infer that the up-regulated expression of GST in mutant vp16 line was crucial to the accumulation of glutathione, ROS scavenging and redox homeostasis maintenance. We also observed increased proline content and POD activity in response to PEG treatment in mutant vp16 (Figure 5A), which resulted in more antioxidant activity and ROS removal, consistent with our previous findings [47]. Increased accumulation of peroxidase, an integral component of the glutathione-ascorbate cycle, is critical for H_2_O_2_ scavenging and improving tolerance to oxidative stress [78]. The abundance of POD was previously reported in salt-tolerant [77] and drought-tolerant maize lines [63,80] suggesting that enhanced synthesis of glutathione and antioxidants, together with other stress-protective and -responsive proteins, is a common strategy for drought-tolerant plants. 

### 3.8. Proposed Hypothetical Model for Maize Germinating-Seed Drought Tolerance

We propose a hypothetical model for maize germinating seeds’ drought tolerance based on the physiological and proteome-level changes identified in the tolerant line mutant vp16 (Figure 6; Table 1 and Table S3). 

The most crucial drought tolerance mechanisms involve cellular CHO and energy metabolism, redox homeostasis maintenance, H2A-mediated PTMs, protein synthesis and proteolysis, stress signal transduction and abiotic–biotic cross-talk. These proteins chiefly participate in the carbon metabolism, pentose phosphate, glutathione metabolism and ribosome pathways. 

## 4. Materials and Methods

### 4.1. Plant Materials and Drought-Stress Treatment

In order to investigate the effect of PEG-induced drought stress on maize seeds during germination, two drought contrary lines, wild-type Vp16 and mutant vp16, were used. The wild-type material was provided by the Hebei Sub-Center of National Maize Improvement Center (College of Agronomy, Hebei Agricultural University, China). For the mutant line, BC4F2 (of Zong31 (Z31) genetic background populations) with novel vivipary mutation locus (vp16) from cross of W22::Mu and Z31 [81] was cultivated in two environments (Baoding, 38°50’N, 115°50’E, 2012; and Sanya, 18°15’N, 109°30’E, 2012) and the genetic analysis of their mutant traits conducted. The original Uniform MU population was provided by the National Maize Improvement Center of China Agricultural University (Beijing, China). The seed grains fetched 28 days post-pollination (Baoding, 38°50’N, 115°50’E, 2015) were used for this study.

For the experiment, we selected the full grain, same size, no-worm-hole maize seeds. The seeds were surface-sterilized with 1% sodium hypochlorite for ten minutes, followed by rinsing with sterile water three times. Then, ten seeds each line were placed on the moist Whatman germination paper (Sigma-Aldrich, Maidstone, UK) in 9-cm diameter sterilized petri dishes and germinated in a greenhouse at 26 ± 1 °C under a 12h light/12h dark cycle (light intensity during the daytime was 350 µmol m^−2^ s^−1^) with the relative humidity of 60–70%. After three days, half of the petri dishes were exposed to osmotic stress by application of 20% polyethylene glycol (PEG)-6000 for 7 days (half of the dishes were treated with distilled water as control). Subsequently, samples were collected for proteomic analysis, quantitative real-time polymerase chain reaction (qRT-PCR) and evaluation of physiological indices. Each treatment was repeated thrice.

### 4.2. Growth Parameter Measurements and Physiological Assays

The germination processes of three biological replicates (10 seeds each replicate) were observed daily and the GR (%) of the seeds was measured after 7 days of PEG treatment. The GR was calculated as follows: GR = (germinated seed number/test seed number) × 100%. Meanwhile, five consistently growing seedlings per replicate were chosen for sample collection. The root and shoot were cut with a shear and the RFW and SFW measured, respectively. The RL and SL were measured using a ruler. Maize seeds’ physiological parameters were assessed after 7 days of treatment with or without PEG. The osmolyte Pro content was determined using ninhydrin-based colorimetric method [82]. The POD activity was estimated by the guaiacol method [83], while SOD activity was determined using the nitroblue tetrazolium photoreduction method [84]. The MDA content in the seeds was measured by thiobarbituric acid method [85]. 

### 4.3. Protein Extraction

Total proteins were extracted from the non-stressed and stressed seed tissues of wild-type Vp16 and mutant vp16 (three biological replicates for each treatment–cultivar combination) using the cold acetone method as described in previous report [86]. In brief, approximately 0.5 g of seeds were ground to a fine powder in liquid nitrogen and further dissolved in 2 mL lysis buffer containing 8 M urea, 2% sodium dodecyl sulfate (SDS) and 1× Protease Inhibitor Cocktail (Thermo Fisher Scientific, Shanghai, China). Then, the solution was incubated on ice for 30 min prior to centrifugation at 12,000 rpm for 15 min at 4 °C. The upper phenol phase was then transferred into new tubes and precipitated with 10% TCA/90% acetone, followed by incubation at −20 °C overnight. After centrifugation at 12,000 rpm for 15 min at 4 °C, the supernatant was discarded and pellets were washed thrice with cold acetone. Finally, the precipitate was dissolved in 8 M urea under ultrasound irradiation. The protein concentration was determined using Pierce Bicinchoninic Acid (BCA) Protein Assay Kit (23225, Thermo Fisher Scientific, Shanghai, China) and absorbance measured at 562 nm using a SpectraMax iD3 Multi-Mode Microplate Reader (Molecular Devices, Shanghai, China) [87]. Protein quality was confirmed with SDS-PAGE (tricine-sodium dodecyl sulfate polyacrylamide gel electrophoresis) [88]. 

### 4.4. Protein Digestion and iTRAQ (Isobaric Tags for Relative and Absolute Quantification) Labeling

Reduction of disulfide bonds and alkylation of free cysteine residues (using 120 μL 55 mM iodoacetamide) were performed prior to protein digestion as fully described in our most recent paper. For digestion of total proteins (100 µg samples), trypsin (Promega, Madison, WI, USA) at a ratio of protein:trypsin of 30:1 at 37 °C overnight (16 h) was used. Post-digestion, the peptides were dried in a centrifugal vacuum concentrator and reconstituted in 0.5 M TEAB. For an explicit description of the digestion process, we refer you to our previous study [63]. Post protein digestion step, Applied Protein Technology Co. Limited (Shanghai, China) performed the iTRAQ labeling procedure using an iTRAQ Reagents 8-plex kit (AB Sciex, Foster City, CA USA) as per the manufacturer’s guidelines. Essentially, a single unit of iTRAQ reagent was liquefied and reorganized in 70 µL isopropanol as previously described [63]. The iTRAQ tags 114 and 115 were used to label the control replicates for drought-sensitive wild-type Vp16 and drought-tolerant mutant vp16 lines, respectively. Additionally, tags 116 and 117 where used to label PEG-treated replicates for wild-type Vp16 and mutant vp16, respectively, with three technical replicates each sample. 

### 4.5. Strong Cation Exchange (SCX) and LC-MS/MS Analysis

Strong cation exchange chromatography was performed with an Agilent 1100 high performance liquid chromatography (HPLC) system (Agilent Technologies, Waldbronn, Germany). The iTRAQ labeled peptide mixtures were reconstituted with a PolySulfoethyl A column (4.6 × 100 mm^2^, 5 µm, 300 Å; PolyLC, Columbia, MD, USA) as per the manufacturer’s guidelines. Each fraction was dissolved in 4 mL buffer A (10 mM KH_2_PO_4_, 25% ACN, pH 3.0), loaded and washed isocratically for 20 min at 0.5 mL/min to remove excess reagent. The retained peptides were then eluted with a linear gradient of 0–500 mM buffer B (10 mM KH_2_PO_4_, 500 mM KCl, 25% ACN, pH 3.0) over 15 min at a flow rate of 1 mL/min. The elution was monitored by absorbance at 214 nm and the fractions were collected every 1 min.

The eluted fractions (about 30 fractions) were combined into 10 pools and desalted with a Strata X C18 column (inner diameter 75 um). Each fraction was subjected to reverse phase nanoflow HPLC separation and quadruple time-of-flight (QSTAR XL) MS analysis [87]. The MS spectra with a mass range of 300–1800 m/z were acquired at a resolving power of 120 K; the primary MS resolution was set to 70,000 at 200 m/z. The automatic gain control for MS was set to 1 × 10^6^, maximum ion injection time was 50 ms and the dynamic exclusion time (active exclusion) was 60.0 s.

### 4.6. Protein Identification and Quantification

As fully described in our recent study [63], the LC-MS/MS raw files were converted into MGF files. MGF as the initial files were searched using Mascot software version 2.2 (Matrix Science, London, UK) embedded in Proteome Discovery 1.4 (Thermo Fisher Scientific Inc., Waltham, MA, USA) against the Uniprot database (uniprot_Zea mays_132339_20180112.FASTA; 76,417 sequences). The Mascot search parameters were set as follows: enzyme, trypsin; fragment mass tolerance was set at ±0.1 Da; peptide mass tolerance was set at ±20 ppm; mass value, monoisotopic; iTRAQ 8-plex (Y) and Oxidation (M) as variable modifications; Carbamidomethyl (C), iTRAQ 8-plex (N-term) and iTRAQ 8-plex (K) were set as fixed modifications. Only unique peptides with a 95% confidence and false discovery rate (FDR) <1% were considered for further analysis. Further normalization of the final protein quantification ratios was conducted using the median average of those ratios. The unique peptide ratios’ median represented the protein ratio. For differential analysis of the relative abundance of peptides and proteins between samples, both of the two values were transformed into log2 scale using the software Perseus (version 1.6.0.7) [89]. The *p*-values provided by Student’s *t*-test were used to reduce the overall false positive; the proteins with a fold-change >1.2 or <0.83 (*p* < 0.05) were considered to be significantly differently expressed. 

### 4.7. Biological Function Classification, Pathway Enrichment and Hierarchal Clustering Analysis of DAPs

To determine the biological functions of the identified DAPs, they were used as queries to search the Interpro (https://www.ebi.ac.uk/interpro/). The expression data were further processed with the hierarchal cluster function of Gene Cluster 3.0 software. The molecular functions of the DAPs were classified GO (http://www.geneontology.org/). Pathway mapping of identified DAPs was performed by KEGG (http://www.genome.jp/kegg/) databases. We employed hypergeometric test to perform GO and KEGG enrichment analysis, with *p*-values less than 0.05 defined as statistically significant to identify candidate biomarkers. Also, the website ReviGO (http://revigo.irb.hr) was used to identify GO functional categories (*p* < 0.05) [77]. 

### 4.8. RNA Extraction, cDNA Synthesis and qRT-PCR Analysis

Total RNA was extracted from seed tissues from control and PEG-treated germinating seeds (for both wild-type Vp16 and mutant vp16) using Omini Plant RNA Kit (DNase I) (CWBIO, Beijing, China). Using HiFiscript cDNA Synthesis Kit (CWBIO, Beijing, China), the extracted RNA was then reverse-transcribed in a 20 µL total volume as per manufacturer’ guidelines. Gene-specific primers for twenty-eight randomly selected DAPs (Table S9) were designed for qRT-PCR analysis using Primer Premier 5 software. The expression of these genes was studied by qRT-PCR in a C1000 (CFX96 Real-Time System) Thermal Cycler (Bio-Rad) using 2× Fast Super EvaGreen ^®^ qPCR Mastermix (US Everbright Inc., city, CA, USA); *GAPDH* was used for housekeeping. Each 1 µL of cDNA template was mixed with 1 µL of each primer (50 pmol), 10 µL of 2× Fast Super EvaGreen ^®^ qPCR Mastermix (US Everbright Inc., city, CA, USA) and 7 µL ddH_2_O in a 20 µL reaction mixture. The PCR schedule was run as follows: 95 °C for 2 min followed by 40 cycles of 95 °C for 10 s and 55 °C for 30 s. The relative expression level of each gene was determined using Livak and Schmittgen’s cycle threshold (2^–ΔΔ^CT) method [90], with three technical replications for each sample. 

### 4.9. Statistical Analysis of Morpho-Physiological Data

We conveniently used the IBM SPSS statistical package (Version 22.0; IBM Corp., Armonk, NY, USA) to analyze morpho-physiological data. For comparison of growth and physiological indices between treatments and across maize lines, two way analysis of variance (ANOVA) and least significant difference (LSD) tests were employed. Additionally, we performed one-way ANOVA and Duncan’s multiple range tests (DMRT) on qRT-PCR data. Meanwhile, to analyze the differences in protein expression levels between the control and PEG-treated maize germinating seed samples of each line, we used Student’s *t*-test.

## 5. Conclusions

We have applied a comprehensive morpho-physiological and proteomic analysis approach to decipher the differential responses of tolerant mutant vp16 and sensitive wild-type Vp16 maize lines to PEG-induced drought stress. At the physiological level, the tolerant line mutant vp16 exhibited better osmotic stress endurance than sensitive line wild-type Vp16, owing to its improved ROS scavenging competency, robust osmotic adjustment as a result of greater cell water retention and enhanced cell membrane stability. Proteomics analysis identified a total of 1200 DAPs responding to osmotic stress. Chief among these proteins were those related to CHO and energy metabolism, histone H2A-mediated epigenetic regulation, protein biogenesis, signal transduction, redox homeostasis and stress-response processes. Most of these proteins participated in the carbon metabolism, pentose phosphate and glutathione metabolism pathways. Overall, our study offers further insights into the mechanisms underpinning maize drought-tolerance and provides essential leads into the further functional validation of the identified drought-responsive proteins

## Figures and Tables

**Table 1:** Drought-responsive maize seed proteins observed specifically in mutant line vp16.

No	Accession ^1^	Gene Name/ID ^2^	Annotation ^3^	Cover. (%) ^4^	Pept. ^5^	Fold Change ^6^	*p*-Value ^7^	KEGG Pathways ^8^
1	B6T8C2	Zm00001d006547	Histone H2A	36.08	2	1.81	7.33 × 10^−3^	
2	B4FJK0	100216750	Histone H2A	33.75	2	1.73	4.53 × 10^−2^	
3	A0A1D6I7Y4	100282031	Peptidylprolyl isomerase	35.29	2	1.32	2.11 × 10^−2^	
4	A1Z197	Zm00001d024755	Uncharacterized protein	12.71	3	1.25	5.05 × 10^−4^	Plant–pathogen interaction
5	B4FVH1	Zm00001d009640	Malate dehydrogenase	69.41	5	1.23	2.66 × 10^−2^	Glyoxylate and dicarboxylate metabolism
6	B7ZYR5	Zm00001d044434	Uncharacterized protein	9.49	3	1.22	2.88 × 10^−2^	
7	C0PHK8	Zm00001d018529	Uncharacterized protein	15.73	6	1.20	4.66 × 10^−2^	
8	B4FR03	100272828	Nucleic acid-binding OB-fold-like protein	48.63	5	0.83	2.97 × 10^−2^	Homologous recombination
9	B4FYN0	100285351	Mitochondrial import inner membrane translocase subunit TIM13	28.74	2	0.83	6.11 × 10^−3^	
10	B4F8Q9	100191413	Calcium ion binding	3.14	2	0.80	2.69 × 10^−2^	
11	K7VPB0	103636586	Pentatricopeptide repeat-containing protein mitochondrial	7.06	3	0.73	6.22 × 10^−3^	

^1^ Accession, protein’s UniProt database identification number; ^2^ gene name/ ID, name or ID as searched against Gramene database (http://ensemble.gramene.org/Zea mays); ^3^ Annotation, biological function characterization as per the Gene Ontology (GO) analysis; ^4^ Coverage, % of protein sequence covered by identified peptides; ^5^ Peptides fragments, number of identified peptide fragments that were matched to the reference database; ^6^ Fold change, the ratio of intensities of up-regulated (>1.2) or down-regulated (<0.83) proteins between PEG treatment and control conditions; ^7^ *p*-value, statistical significant level (Student’s *t*-test, <0.05). ^8^ KEGG pathway, enriched metabolic pathway as per the Kyoto Encylopedia of Genes and Genomics (KEGG) database.

## Supplementary Materials

Supplementary materials can be found at https://www.mdpi.com/1422-0067/20/22/5586/s1. The mass spectrometry proteomics data have been deposited into the ProteomeXchange Consortium Database (http://proteomecentral.proteomexchange.org) via the iProX partner repository with the dataset identifier PXD015533.

## Abbreviations

CDPKs	Calcium dependent protein kinases
DAPs	Differentially abundant proteins
GADPH	Glyceraldehyde-3-phosphate dehydrogenase
GO	Gene ontology
GSTs	Glutathione-S-transferases
HSPs	Heat shock proteins
iTRAQ	Isobaric tags for relative and absolute quantification
KEGG	Kyoto Encyclopedia of Genes and Genomes
LC-MS/MS	Liquid chromatography-tandem mass spectrometry
MAPK	Mitogen-activated protein kinases
MDA	Malondialdehyde
MDH	Malate dehydrogenase
nsLTPs	Non-specific lipid transfer proteins
PEG	Polyethylene-glycol
POD	Guaiacol peroxidase
PPIase	Peptidylprolyl isomerase
qRT-PCR	Quantitative real-time polymerase chain reaction
RPs	Ribosomal proteins
ROS	Reactive oxygen species
SOD	Superoxide dismutase

## References

[1] Al-Whaibi M.H. (2010). Plant heat-shock proteins: A mini review. J. King Saud Univ. Sci..

[2] Farooq M., Wahid A., Kobayashi N., Fujita D., Basra S.M.A. (2009). Plant drought stress: Effects, mechanisms and management. Agron. Sustain. Dev..

[3] Aslam M., Maqbool M.A., Cengiz R. (2015). Drought stress in maize (*Zea mays* L.): Effects, resistance mechanisms, global achievements and biological strategies for improvement. SpringerBriefs in Agriculture.

[4] Miao Z., Han Z., Zhang T., Chen S., Ma C. (2017). A systems approach to spatio-temporal understanding of the drought stress response in maize. Sci. Rep..

[5] Fahad S., Bajwa A.A., Nazir U., Anjum S.A., Farooq A., Zohaib A., Sadia S., Nasim W., Adkins S., Saud S. (2017). Crop production under drought and heat Stress: Plant responses and management options. Front. Plant Sci..

[6] Feller U., Vaseva I.I. (2014). Extreme climatic events: Impacts of drought and high temperature on physiological processes in agronomically important plants. Front. Environ. Sci..

[7] F.A.O (Food and Agricultural Organization of the United Nations) (2009). High Level Expert Forum—How to Feed the World in 2050. www.fao.org/.

[8] Nepolean T., Kaul J., Mukri G. (2018). Genomics-enabled next-generation breeding approaches for developing system-specific drought tolerant hybrids in maize. Front. Plant Sci..

[9] Thirunavukkarasu N., Sharma R., Singh N., Shiriga K., Mohan S., Mittal S., Mittal S., Mallikarjuna M.G., Rao A.R., Dash P.K. (2017). Genomewide expression and functional interactions of genes under drought stress in maize. Int. J. Genom..

[10] Shiferaw B., Prasanna B.M., Hellin J., Bänziger M. (2011). Crops that feed the world 6. Past successes and future challenges to the role played by maize in global food security. Food Secur..

[11] Gong F., Yang L., Tai F., Hu X., Wang W. (2014). “Omics” of maize stress response for sustainable food production: Opportunities and challenges. OMICS.

[12] Yin X.G., Jørgen E.O., Wang M., Kersebaum K.-C., Chen H., Baby S., Ozturk I., Chen F. (2016). Adapting maize production to drought in the northeast farming region of China. Eur. J. Agron..

[13] Kaya M.D., Okçu G., Atak M., Çıkılı Y., Kolsarıcı Ö. (2006). Seed treatments to overcome salt and drought stress during germination in sunflower (*Helianthus annuus* L.). Eur. J. Agron..

[14] Hussain H.A., Hussain S., Khaliq A., Ashraf U., Anjum S.A., Men S.N., Wang L.C. (2018). Chilling and drought stresses in crop plants: Implications, cross talk, and potential management opportunities. Front. Plant Sci..

[15] Wu S., Ning F., Zhang Q., Wu X., Wang W. (2017). Enhancing omics research of crop responses to drought under field conditions. Front. Plant Sci..

[16] Khodarahmpour Z. (2017). Evaluation of maize (*Zea mays* L.) hybrids, seed germination and seedling characters in water stress conditions. Afr. J. Agric. Res..

[17] Álvarez-Iglesias L., de la Roza-Delgado B., Reigosa M.J., Revilla P., Pedrol N. (2017). A simple, fast and accurate screening method to estimate maize (*Zea mays* L) tolerance to drought at early stages. Maydica.

[18] Agrawal L., Gupta S., Mishra S.K., Pandey G., Kumar S., Chauhan P.S., Chakrabarty D., Nautiyal C.S. (2016). Elucidation of complex nature of PEG induced drought-stress response in rice root using comparative proteomics approach. Front. Plant Sci..

[19] Zhao Y., Wang Y., Yang H., Wang W., Wu J., Hu X. (2016). Quantitative proteomic analyses identify aba-related proteins and signal pathways in maize leaves under drought conditions. Front. Plant Sci..

[20] Osmolovskaya N., Shumilina J., Kim A., Didio A., Grishina T., Bilova T., Keltsieva O.A., Zhukov V., Tikhonovich I., Tarakhovskaya E. (2018). Methodology of drought stress research: Experimental setup and physiological characterization. Int. J. Mol. Sci..

[21] Wang X.L., Cai X.F., Xu C.X., Wang Q.H., Dai S.J. (2016). Drought-responsive mechanisms in plant leaves revealed by proteomics. Int. J. Mol. Sci..

[22] Wahid A., Gelani S., Ashraf M., Foolad M.R. (2007). Heat tolerance in plants: An overview. Environ. Exp. Bot..

[23] Shan X., Li Y., Jiang Y., Jiang Z., Hao W., Yuan Y. (2013). Transcriptome profile analysis of maize seedlings in response to high-salinity, drought and cold stresses by deep sequencing. Plant Mol. Biol. Rep..

[24] Min H., Chen C., Wei S., Shang X., Sun M., Xia R., Liu X.G., Hao D.Y., Chen H.B., Xie Q. (2016). Identification of drought tolerant mechanisms in maize seedlings based on transcriptome analysis of recombination inbred lines. Front. Plant Sci..

[25] Zhao Q., Zhang H., Wang T., Chen S., Dai S. (2013). Proteomics-based investigation of salt-responsive mechanisms in plant roots. J. Proteom..

[26] Kosova K., Vitamvas P., Prasil I.T., Renaut J. (2011). Plant proteome changes under abiotic stress- contribution of proteomics studies to understanding plant stress response. J. Proteom..

[27] Kamel C., Sonia A.R., Claudette J., Dominique J. (2006). Proteomic analysis of seed dormancy in Arabidopsis. Physiol. Plant.

[28] Zhang H., Lian C., Shen Z. (2009). Proteomic identification of small, copper-responsive proteins in germinating embryos of Oryza sativa. Ann. Bot..

[29] ProtTech (2010). Two Different Methods in Protein Identification by Mass Spectrometry.. http://www.prottech.com/.

[30] Wu X., Wang W. (2016). Increasing confidence of proteomics data regarding the identification of stress-responsive proteins in crop plants. Front. Plant Sci..

[31] Kamal A.H.M., Cho K., Choi J.S., Jin Y., Park C.S., Lee J.S., Woo S.H. (2013). Patterns of protein expression in water-stressed wheat chloroplasts. Biol. Plant..

[32] Cheng L., Gao X., Li S., Shi M., Javeed H., Jing X., Yang G., He G. (2010). Proteomic analysis of soybean [*Glycine max* (L.) Meer.] seeds during imbibition at chilling temperature. Mol. Breed..

[33] Zheng J., Fu J., Gou M., Huai J., Liu Y., Jian M., Huang Q., Guo X., Dong Z., Wang H. (2010). Genome-wide transcriptome analysis of two maize inbred lines under drought stress. Plant Mol. Biol..

[34] Anjum S.A., Xie X.Y., Wang L.C., Saleem M.F., Man C., Lei W. (2011). Morphological, physiological and biochemical responses of plants to drought stress. Afr. J. Agric. Res..

[35] Khodarahmpour Z., Motamedi M. (2011). Evaluation of drought and salinity stress effects on germination and early growth of two cultivars of maize (*Zea mays* L.). Afr. J. Biotechnol..

[36] Westgate M.E., Boyer J.S. (1985). Osmotic adjustment and the inhibition of leaf, root, stem and silk growth at low water potentials in maize. Planta.

[37] Moussa H.R., Abdel-Aziz S.M. (2008). Comparative response of drought tolerant and drought sensitive maize genotypes to water stress. Aust. J. Crop Sci..

[38] Mittler R. (2002). Oxidative stress, antioxidants and stress tolerance. Trends Plant Sci..

[39] Sharma P., Jha A.B., Dubey R.S., Pessarakli M. (2012). Reactive oxygen species, oxidative damage, and antioxidative defense mechanism in plants under stressful conditions. J. Bot..

[40] Fang Y., Xiong L. (2015). General mechanisms of drought response and their application in drought resistance improvement in plants. Cell. Mol. Life Sci..

[41] Wang T., Chen X., Zhu F., Li F., Li L., Yang Q., Chi X., Yu S. (2013). Characterization of peanut germin-like proteins, AhGLPs in plant development and defense. PLoS ONE.

[42] Caruso G., Cavaliere C., Foglia P., Gubbiotti R., Samperi R., Laganà A. (2009). Analysis of drought responsive proteins in wheat (*Triticum durum*) by 2D-PAGE and MALDI-TOF mass spectrometry. Plant Sci..

[43] Salekdeh G.H., Siopongco J., Wade L.J., Ghareyazie B., Bennett J. (2002). Proteomic analysis of rice leaves during drought stress and recovery. Proteomics.

[44] Urban M.O., Vasek J., Klima M., Krtkova J., Kosova K., Prasil I.T., Vitamvas P. (2016). Proteomic and physiological approach reveals drought- induced changes in rapeseeds: Water-saver and water- spender strategy. J. Proteom..

[45] Vitamvas P., Urban M.O., Škodáček K., Kosova K., Pitelkova I., Vitamvas J., Renaut J., Prášil I.T. (2015). Quantitative analysis of proteome extracted from barely crowns grown under different drought condition. Front. Plant Sci..

[46] Zhang X., Lei L., Lai J., Zhao H., Song W. (2018). Effects of drought stress and water recovery on physiological responses and gene expression in maize seedlings. BMC Plant Biol..

[47] Zenda T., Liu S., Wang X., Liu G., Jin H., Dong A., Yang Y., Duan H. (2019). Key maize drought-responsive genes and pathways revealed by comparative transcriptome and physiological analyses of contrasting inbred lines. Int. J. Mol. Sci..

[48] Kim J.M., Sasaki T., Ueda M., Sako K., Seki M. (2015). Chromatin changes in response to drought, salinity, heat, and cold stresses in plants. Front. Plant Sci..

[49] Mozgova I., Mikulski P., Pecinka A., Farrona S. (2019). Epigenetic mechanisms of abiotic stress response and memory in plants. Epigenet. Plants Agron. Import. Fundam. Appl..

[50] Yuan L., Liu X., Luo M., Yang S., Wu K. (2013). Involvement of histone modifications in plant abiotic stress responses. J. Integr. Plant Biol..

[51] Luo F., Deng X., Liu Y., Yan Y. (2018). Identification of phosphorylation proteins in response to water deficit during wheat flag leaf and grain development. Bot. Stud..

[52] Kim Y.E., Hipp M.S., Bracher A., Hayer-Hartl M., Ulrich Hartl F. (2013). Molecular chaperone functions in protein folding and proteostasis. Annu. Rev. Biochem..

[53] Fotovat R., Alikhani M., Valizadeh M., Mirzaei M., Salekdeh G.H. (2017). A proteomics approach to Discover drought tolerance proteins in wheat pollen grain at meiosis stage. Protein Pept. Lett..

[54] Yang Q., Wang Y., Zhang J., Shi W., Qian C., Peng X. (2007). Identification of aluminum-responsive proteins in rice roots by a proteomic approach: Cysteine synthase as a key player in Al response. Proteomics.

[55] Kaur G., Singh S., Singh H., Chawla M., Dutta T., Kaur H., Bender K., Snedden W.A., Kapoor S., Pareek A. (2015). Characterization of peptidyl-prolyl cis-trans isomerase- and calmodulin-binding activity of a cytosolic Arabidopsis thaliana cyclophilin AtCyp19-3. PLoS ONE.

[56] Kim S.G., Lee J.S., Kim J.T., Kwon Y.S., Bae D.W., Bae H.H., Son B.Y., Baek S.B., Kwon Y.U., Woo M.O. (2015). Physiological and proteomic analysis of the response to drought stress in an inbred Korean maize line. Plant Omics.

[57] Sharma A.D., Singh P. (2003). Comparative studies on drought-induced changes in peptidyl prolyl cis-trans isomerase activity in drought-tolerant and susceptible cultivars of Sorghum bicolor. Curr. Sci..

[58] Zhu J.K. (2016). Abiotic stress signaling and responses in plants. Cell.

[59] Cramer G.R., Urano K., Delrot S., Pezzotti M., Shinozaki K. (2011). Effects of abiotic stress on plants: A systems biology perspective. Biogeochemistry.

[60] Jonak C., Kiegerl S., Ligterink W., Barker P.J., Huskisson N.S., Hirt H. (1996). Stress signaling in plants: A mitogen-activated protein kinase pathway is activated by cold and drought. Proc. Natl. Acad. Sci. USA.

[61] Edstam M.M., Viitanen L., Salminen T.A., Edqvist J. (2011). Evolutionary history of the non-specific lipid transfer proteins. Mol. Plant.

[62] Molina A., Segura A., García-Olmedo F. (1993). Lipid transfer proteins (nsLTPs) from barley and maize leaves are potent inhibitors of bacterial and fungal plant pathogens. FEBS Lett..

[63] Zenda T., Liu S., Wang X., Jin H., Liu G., Duan H. (2018). Comparative proteomic and physiological analyses of two divergent maize inbred lines provide more insights into drought-stress tolerance mechanisms. Int. J. Mol. Sci..

[64] Pitzschke A., Forzani C., Hirt H. (2006). Reactive oxygen species signaling in plants. Antioxid. Redox Signal..

[65] Gill S.S., Tuteja N. (2010). Reactive oxygen species and antioxidant machinery in abiotic stress tolerance in crop plants. Plant Physiol. Biochem..

[66] Harb A. (2016). Identification of candidate genes for drought stress tolerance. Drought Stress Toler. Plants.

[67] Schloß P., Walter C., Mäder M. (1987). Basic peroxidases in isolated vacuoles of *nicotiana tabacum* L.. Planta.

[68] Faghani E., Gharechahi J., Komatsu S., Mirzaei M., Khavarinejad R.A., Najafi F., Farsad L.K., Salekdeh G.H. (2015). Comparative physiology and proteomic analysis of two wheat genotypes contrasting in drought tolerance. J. Proteom..

[69] Benešová M., Holá D., Fischer L., Jedelský P.L., Hnilička F., Wilhelmová N., Rothová O., Kočová M., Procházková D., Honnerová J. (2012). The physiology and proteomics of drought tolerance in maize: Early stomatal closure as a cause of lower tolerance to short-term dehydration?. PLoS ONE.

[70] Xu C.P., Huang B. (2010). Comparative analysis of drought responsive proteins in kentucky bluegrass cultivars contrasting in drought tolerance. Crop Sci..

[71] Zang X., Komatsu S. (2007). A proteomics approach for identifying osmotic-stress-related proteins in rice. Phytochemistry.

[72] Ji W., Zhu Y.M., Li Y., Yang L.A., Zhao X.W., Cai H., Bai X. (2010). Over-expression of a glutathione S-transferase gene, GsGST, from wild soybean (Glycine soja) enhances drought and salt tolerance in transgenic tobacco. Biotechnol. Lett..

[73] Waters E.R., Lee G.J., Vierling E. (1996). Evolution, structure and function of the small heat shock proteins in plants. J. Exp. Bot..

[74] Meyer Y., Siala W., Bashandy T., Riondet C., Vignols F., Reichheld J.P. (2008). Glutaredoxins and thioredoxins in plants. Biochem. Biophys. Acta.

[75] Campbell S.A., Close T.J. (1997). Dehydrins: Genes, proteins, and associations with phenotypic traits. New Phytol..

[76] Hanin M., Brini F., Ebel C., Toda Y., Takeda S., Masmoudi K. (2011). Plant dehydrins and stress tolerance versatile proteins for complex mechanisms. Plant Signal. Behav..

[77] Luo M., Zhao Y., Wang Y., Shi Z., Zhang P., Zhang Y., Song W., Zhao J. (2018). Comparative proteomics of contrasting maize genotypes provides insights into salt-stress tolerance mechanisms. J. Proteome Res..

[78] Zhao X., Bai X., Jiang C., Li Z. (2019). Phosphoproteomic analysis of two contrasting maize inbred lines provides insights into the mechanism of salt-stress tolerance. Int. J. Mol. Sci..

[79] Szalai G., Kellős T., Galiba G., Kocsy G. (2009). Glutathione as an antioxidant and regulatory molecule in plants under abiotic stress conditions. J. Plant Growth Regul..

[80] Zeng W., Peng Y., Zhao X., Wu B., Chen F., Ren B., Zhuang Z., Gao Q., Ding Y. (2019). Comparative proteomics analysis of the seedling root response of drought-sensitive and drought-tolerant maize varieties to drought stress. Int. J. Mol. Sci..

[81] Yang W.F., Tian Y.H., Wang T.T., Wang R.N., Tao Y.S. (2017). Isolating and confirming the *MuDR*-inserted flanking sequences of maize. Cytol. Genet..

[82] Bates T.S., Waldren R.P., Teare I.D. (1973). Rapid determination of free proline for water-stress studies. Plant Soil.

[83] Han L.B., Song G.L., Zhang X. (2008). Preliminary observation of physiological responses of three turfgrass species to traffic stress. HortTechnology.

[84] Ma Q.L., Kang J.M., Long R.C., Cui Y.J., Zhang T.J., Xiong J.B., Yang Q.C., Sun Y. (2016). Proteomic analysis of salt and osmotic-drought stress in alfalfa seedlings. J. Integr. Agric..

[85] Dhindsa R.S., Plumb-Dhindsa P., Thorpe T.A. (1981). Leaf senescence: Correlated with increased leaves of membrane permeability and lipid peroxidation, and decreased levels of superoxide dismutase and catalase. J. Exp. Bot..

[86] Wisniewski J.R., Zougman A., Nagaraj N., Mann M. (2009). Universal sample preparation method for proteome analysis. Nat. Methods.

[87] Zhang C., Shi S. (2018). Physiological and proteomic responses of contrasting alfalfa (*Medicago sativa* L.) varieties to PEG-Induced osmotic stress. Front. Plant Sci..

[88] Swägger H. (2006). Tricine-SDS-PAGE. Nat. Protoc..

[89] Tyanova S., Temu T., Sinitcyn P., Carlson A., Hein M.Y., Geiger T., Mann M., Cox J. (2016). The Perseus computational platform for comprehensive analysis of (prote)omics data. Nat. Method.

[90] Livak K.J., Schmittgen T.D. (2001). Analysis of relative gene expression data using real-time quantitative PCR and the 2−ΔΔCT method. Methods.

