# Supplementary material for: Comparative Proteomic and Morpho-Physiological Analyses of Maize Wild-Type Vp16 and Mutant vp16 Germinating Seed Responses to PEG-Induced Drought Stress
Source: Int J Mol Sci. 2019 Nov 8;20(22):5586. doi: 10.3390/ijms20225586 (PMC6888951; doi:10.3390/ijms20225586)
Supplement: Supplementary file 1 [file ijms-20-05586-s001.zip › Supplemantery Tables/Supplementary Table 7.doc]

**Supplementary Table 7.** Twenty-eight (28) representative genes selected for qRT-PCR validation

| **No.** | **Asseccion^1^** | **Gene ID/Name^2^** | **Description^3^** |
| --- | --- | --- | --- |
| 1 | A0A1D6MXK3 | Zm00001d041663 | Jasmonate-induced protein |
| 2 | B4FRS8 | 100272932 | Germin-like protein subfamily T member 1 |
| 3 | B6TFB6 | 100283176 | Stress responsive protein |
| 4 | B6T3V1 | 100282124 | Peroxidase |
| 5 | O82087 | PR-5 | Pathogenesis related protein-5 |
| 6 | B6U7D8 | Zm00001d015618 | Cinnamyl alcohol dehydrogenase |
| 7 | Q9FQA9 | 541841 | Glutathione S-transferase GST 30 |
| 8 | B4FGG7 | 100193836 | Calcium ion binding protein |
| 9 | B4F848 | 100191248 | 20 kDa chaperonin chloroplastic |
| 10 | B7ZEQ0 | hsp18 | Small heat-shock protein |
| 11 | B4G024 | 100193491 | Malate dehydrogenase |
| 12 | B6SLU3 | 100280779 | Aldehyde dehydrogenase |
| 13 | B8QWQ8 | PRms | Pathogenesis-related maize seed protein |
| 14 | Q9FQA3 | Zm00001d020780 | Glutathione transferase GST 23 |
| 15 | B4G231 | Zm00001d005890 | Alpha-amylase |
| 16 | A0A1D6JTN4 | Zm00001d028260 | Glutamine synthetase |
| 17 | A0A1D6K1T6 | 103634611 | 23-bisphosphoglycerate-independent phosphoglycerate mutase 1 |
| 18 | A0A1D6H1J8 | 100282981 | Glyceraldehyde-3-phosphate dehydrogenase |
| 19 | B6TX09 | 100284739 | Fructose-1,6-bisphosphatase, cytosolic |
| 20 | B4FT23 | 100283140 | 14-3-3-like protein |
| 21 | K7UY19 | 100502020 | Fructose-bisphosphate aldolase |
| 22 | B6SQM6 | 100280981 | Pathogenesis-related protein 10 |
| 23 | A0A1D6KL85 | Zm00001d031773 | HVA22-like protein |
| 24 | D2IPC3 | bt2 | ADP-glucose pyrophosphorylase small subunit |
| 25 | B4FVH1 | Zm00001d009640 | Malate dehydrogenase |
| 26 | B6T8C2 | Zm00001d006547 | Histone H2A |
| 27 | A0A1D6I7Y4 | 100282031 | Peptidylprolyl isomerase |
| 28 | K7VPB0 | 103636586 | Pentatricopeptide repeat-containing protein mitochondrial |

^1^ Accession, unique protein identifying number in the UniProt database; ^2^Gene ID/name, ID number of the corresponding gene of the identified differentially abundant protein as searched against the maize sequence database Gramene ([http://ensemble.gramene.org/Zea mays](http://ensemble.gramene.org/Zea%20mays)); ^3^ Description, annotated biological functions based on Gene Ontology (GO) analysis.
