# Supplementary material for: Comparative Proteomic and Morpho-Physiological Analyses of Maize Wild-Type Vp16 and Mutant vp16 Germinating Seed Responses to PEG-Induced Drought Stress
Source: Int J Mol Sci. 2019 Nov 8;20(22):5586. doi: 10.3390/ijms20225586 (PMC6888951; doi:10.3390/ijms20225586)
Supplement: Supplementary file 1 [file ijms-20-05586-s001.zip › Supplementary Figures.pdf]

Supplementary Figures (Total 7)

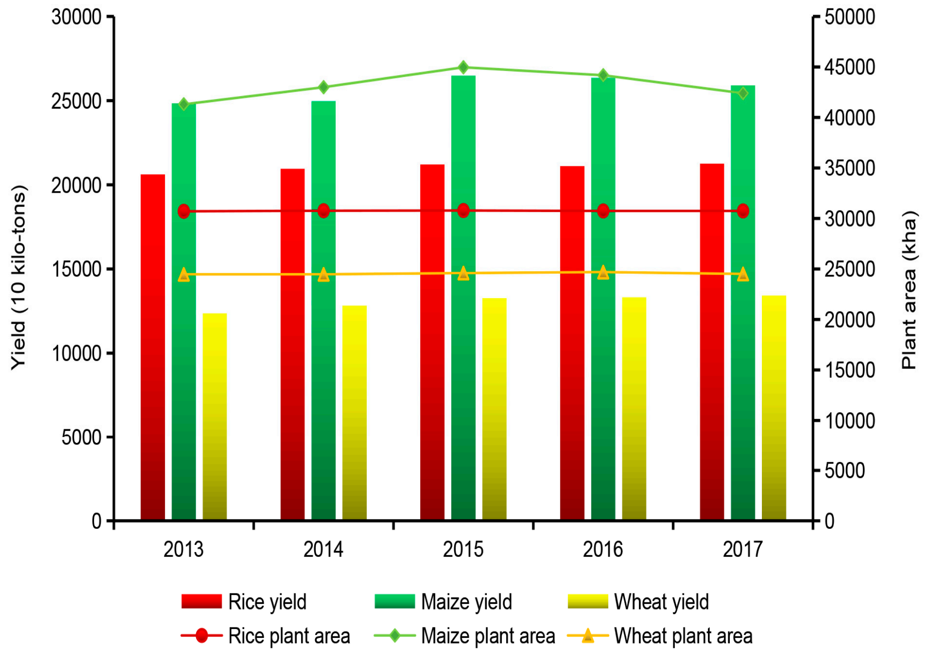

Supplementary Figure 1. The main food crops planting area and yield in recent years (2013-2017) in China (http://data.stats.gov.cn/index.htm)

2A

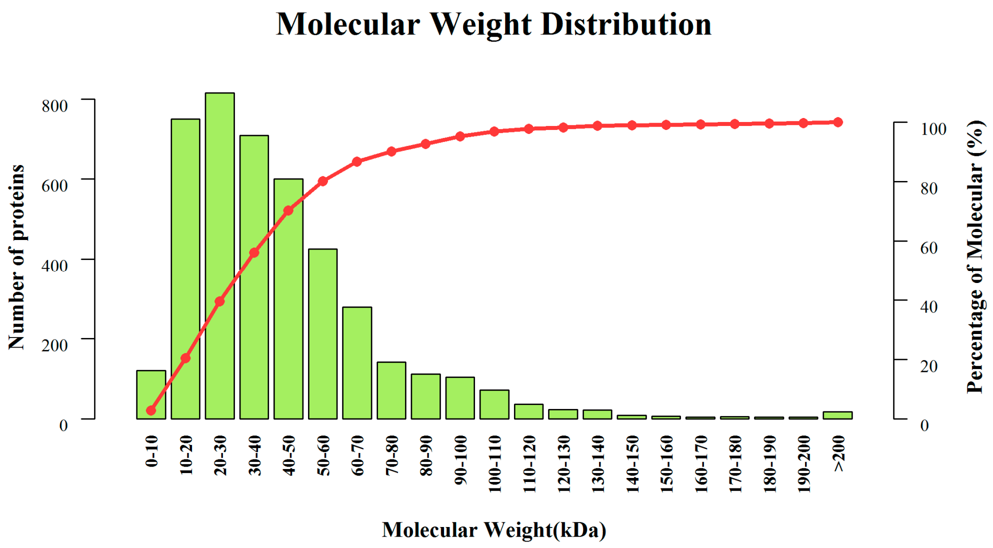

Molecular Weight Distribution
Number of proteins
800
600
400
200
0
0-10
10-20
20-30
30-40
40-50
50-60
60-70
70-80
80-90
90-100
100-110
110-120
120-130
130-140
140-150
150-160
160-170
170-180
180-190
190-200
>200
Molecular Weight(kDa)
100
80
60
40
20
0
Percentage of Molecular (%)

2B

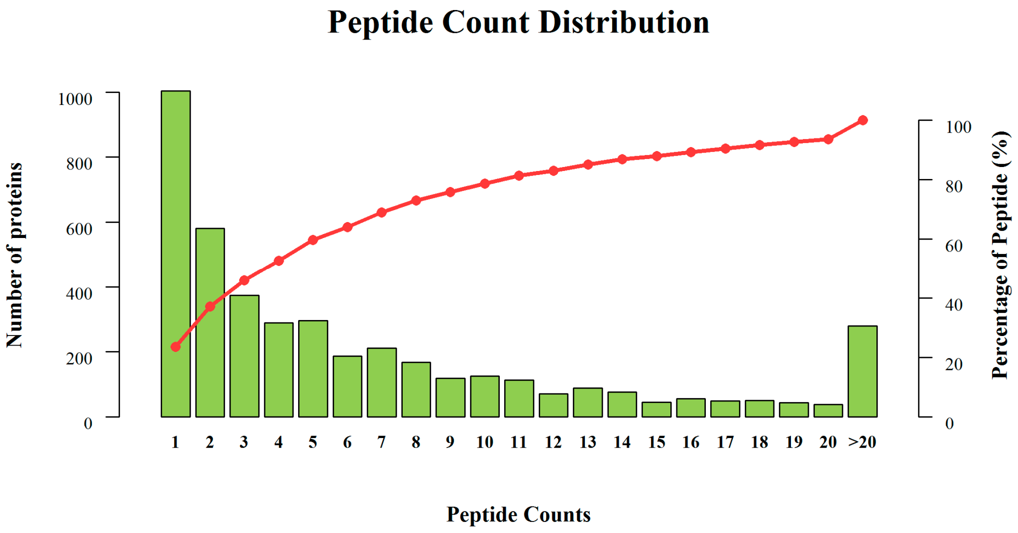

Peptide Count Distribution
Number of proteins
1000
800
600
400
200
0
1
2
3
4
5
6
7
8
9
10
11
12
13
14
15
16
17
18
19
20
>20
Peptide Counts
100
80
60
40
20
0
Percentage of Peptide (%)

2C

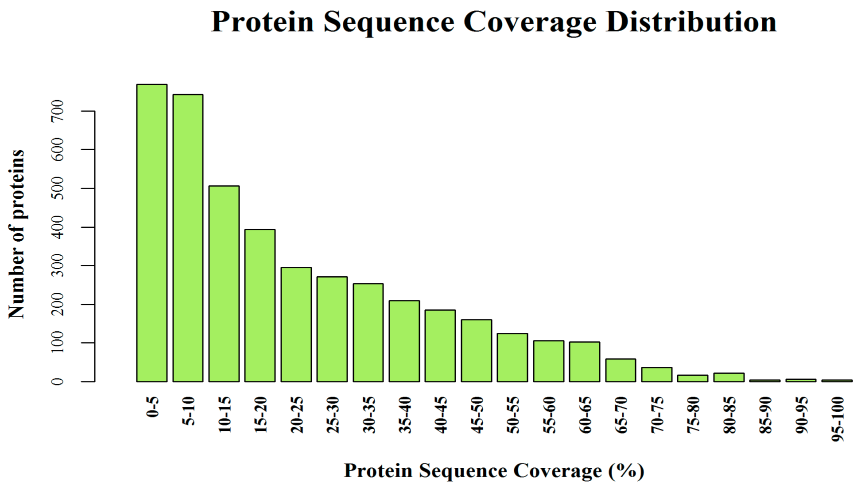

2D

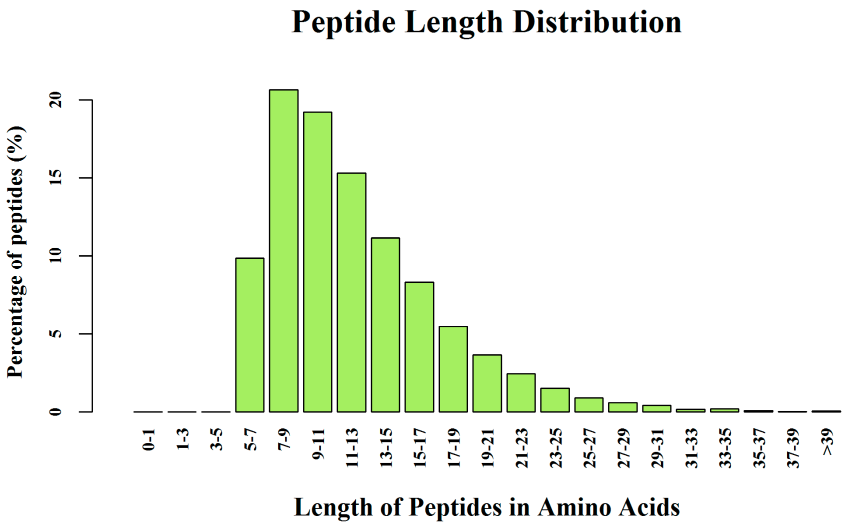

Supplementary Figure 2. Basic isobaric tags for relative and absolute quantification (iTRAQ) proteomics analysis output details. **(A)** Mass distribution of the identified proteins; **(B)** numbers of peptides that were matched to proteins; **(C)** sequence coverage range of the identified proteins; **(D)** length distribution of peptides defining each protein.

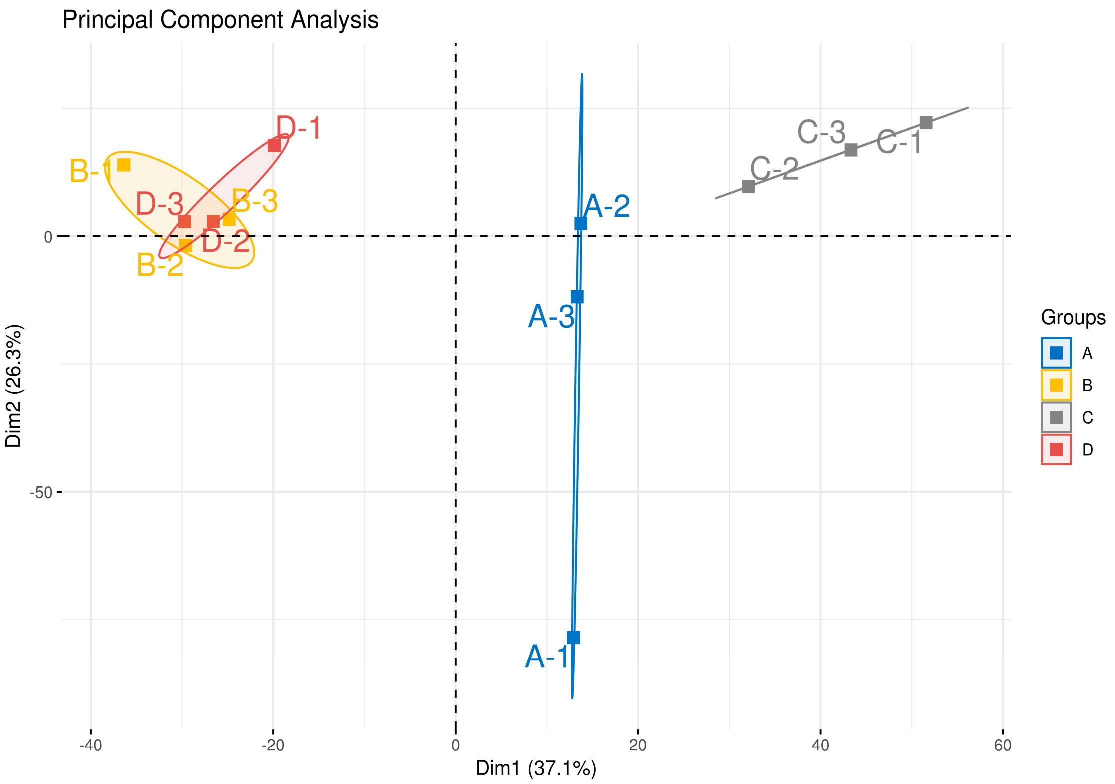

Supplementary Figure 3. Principal component (PCA) analysis of the twelve maize germinating-seed-tissue samples. PCA analysis was conducted using the dimension reduction and clustering visualization procedure. An orthogonal transformation was used to identify the principal components, which equal a linear combination of the protein levels. The distance of between two sample points represents the similarity level of those samples; the closer the distance, the higher the similarity between the samples. X axis represents the degree of contribution of a principal component N (PCN) in the two-dimensional graph to distinguish samples; Y axis represents the contribution of a principal component M (PCM) in the two-dimensional graph to distinguish samples.

4A

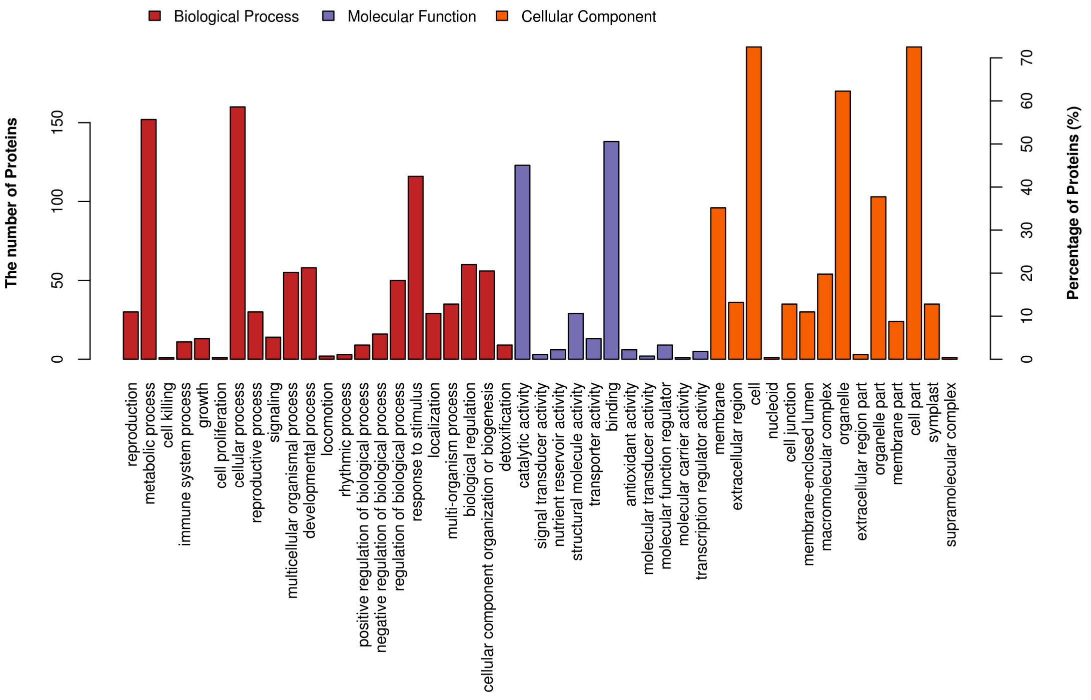
Biological Process
Molecular Function
Cellular Component
The number of Proteins
Percentage of Proteins (%)
reproduction
metabolic process
cell killing
immune system process
growth
cell proliferation
cellular process
reproductive process
signaling
multicellular organismal process
developmental process
locomotion
rhythmic process
positive regulation of biological process
negative regulation of biological process
regulation of biological process
response to stimulus
localization
multi-organism process
biological regulation
cellular component organization or biogenesis
detoxification
catalytic activity
signal transducer activity
nutrient reservoir activity
structural molecule activity
transporter activity
binding
antioxidant activity
molecular transducer activity
molecular function regulator
molecular carrier activity
transcription regulator activity
membrane
extracellular region
cell
nucleoid
cell junction
membrane-enclosed lumen
macromolecular complex
organelle
extracellular region part
organelle part
membrane part
cell part
symplast
supramolecular complex

4B

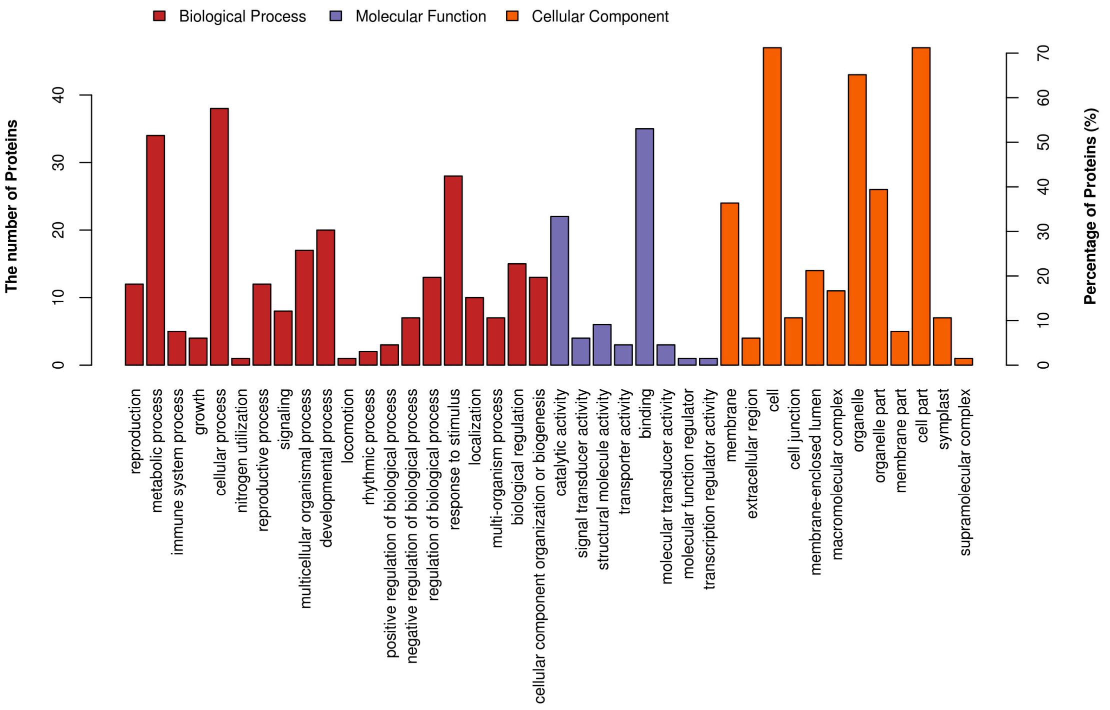
Biological Process
Molecular Function
Cellular Component
The number of Proteins
Percentage of Proteins (%)
reproduction
metabolic process
immune system process
growth
cellular process
nitrogen utilization
reproductive process
signaling
multicellular organismal process
developmental process
locomotion
rhythmic process
positive regulation of biological process
negative regulation of biological process
regulation of biological process
response to stimulus
localization
multi-organism process
biological regulation
cellular component organization or biogenesis
catalytic activity
signal transducer activity
structural molecule activity
transporter activity
binding
molecular transducer activity
molecular function regulator
transcription regulator activity
membrane
extracellular region
cell
cell junction
membrane-enclosed lumen
macromolecular complex
organelle
organelle part
membrane part
cell part
symplast
supramolecular complex

4C

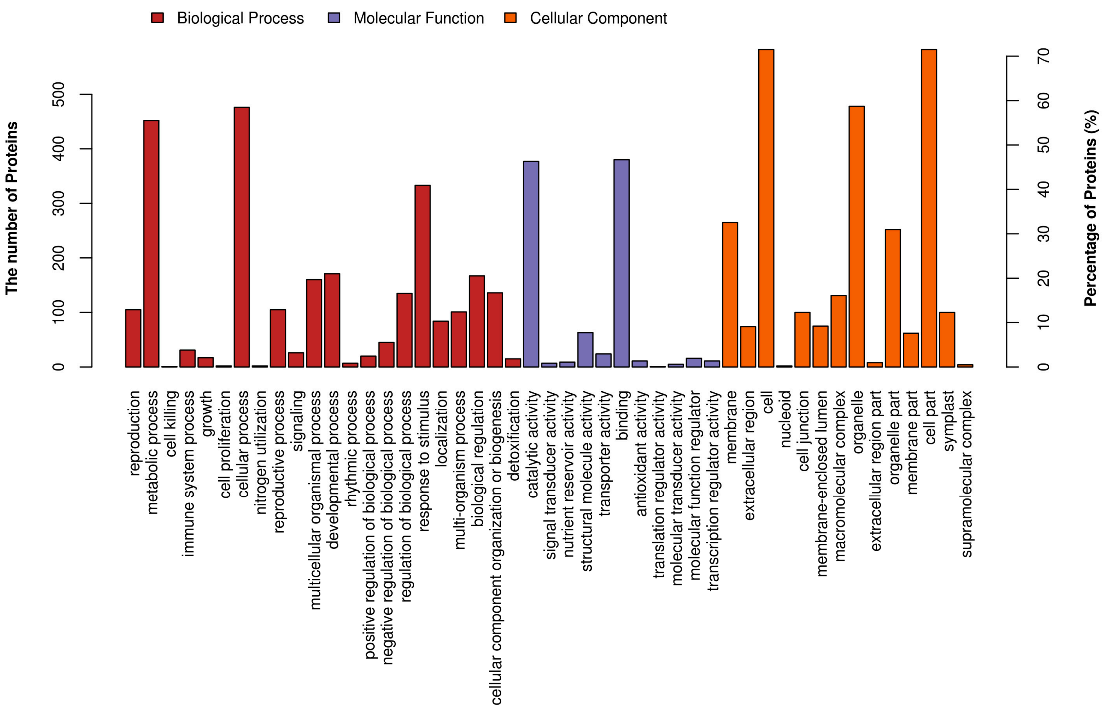

Supplementary Figure 4. Gene ontology (GO) functional classification of drought responsive differentially abundant proteins (DAPs) in different experimental comparisons. **(A)** WC_WD specific DAPs; **(B)** MC_MD specific DAPs; and (**C**) WD_MD specific DAPs. Y-axis represents the number of proteins in each function (and %); The X-axis shows the function of the protein, which is divided into three broad biological functional groups-biological process (BP), molecular function (MF) and cellular component (CC).

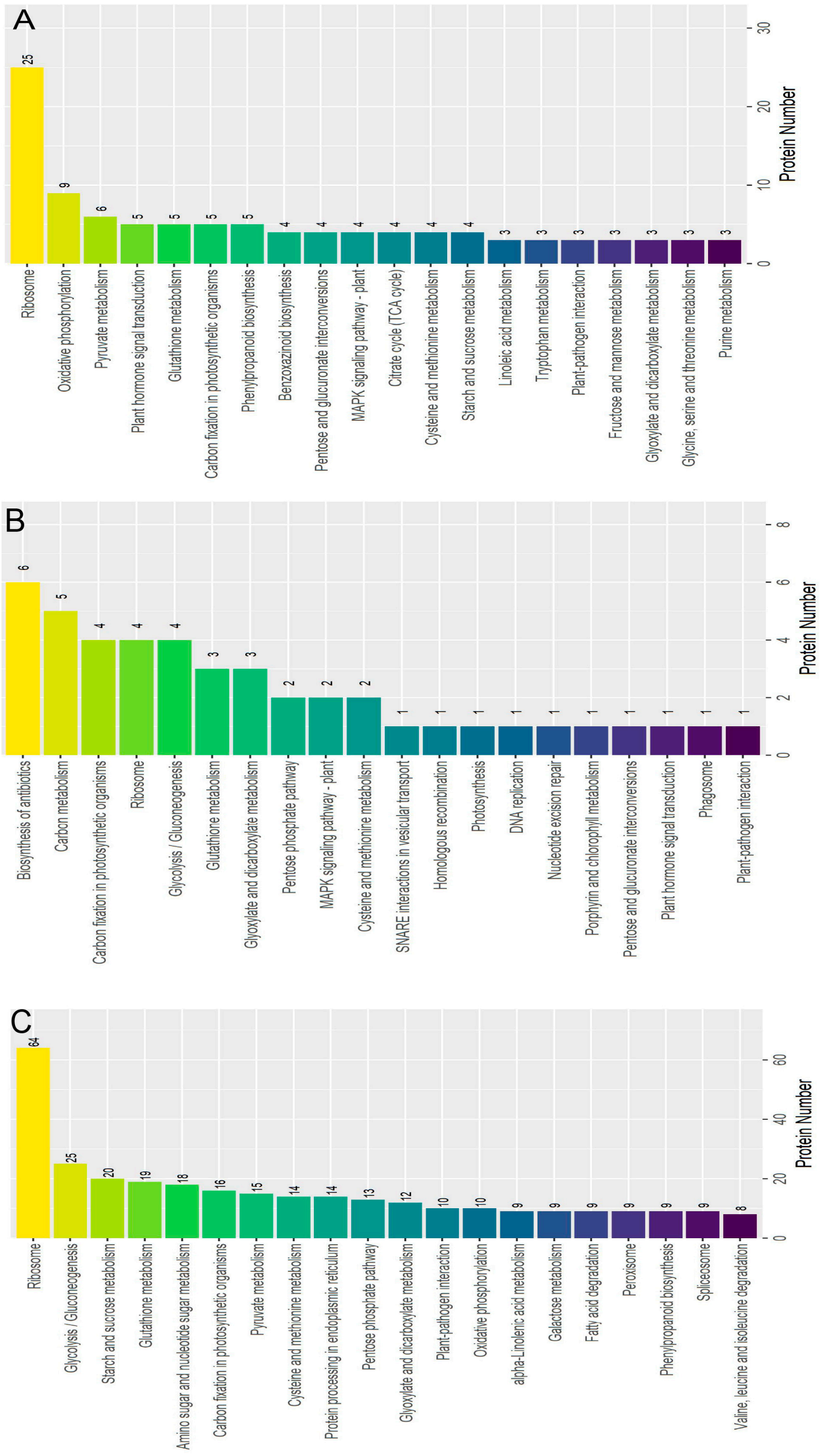

Supplementary Figure 5. Kyoto Encyclopedia of Genes and Genomes (KEGG) pathway analysis of the DAPs based on the KEGG database. **(A)** WC_WD; **(B)** MC_MD; and **(C)** WD_MD experimental comparisons. The y-axis represents number of DAPs enriched in the corresponding pathway, and the x-axis indicates the specific sub-categories of DAPs in that main pathway.

6A

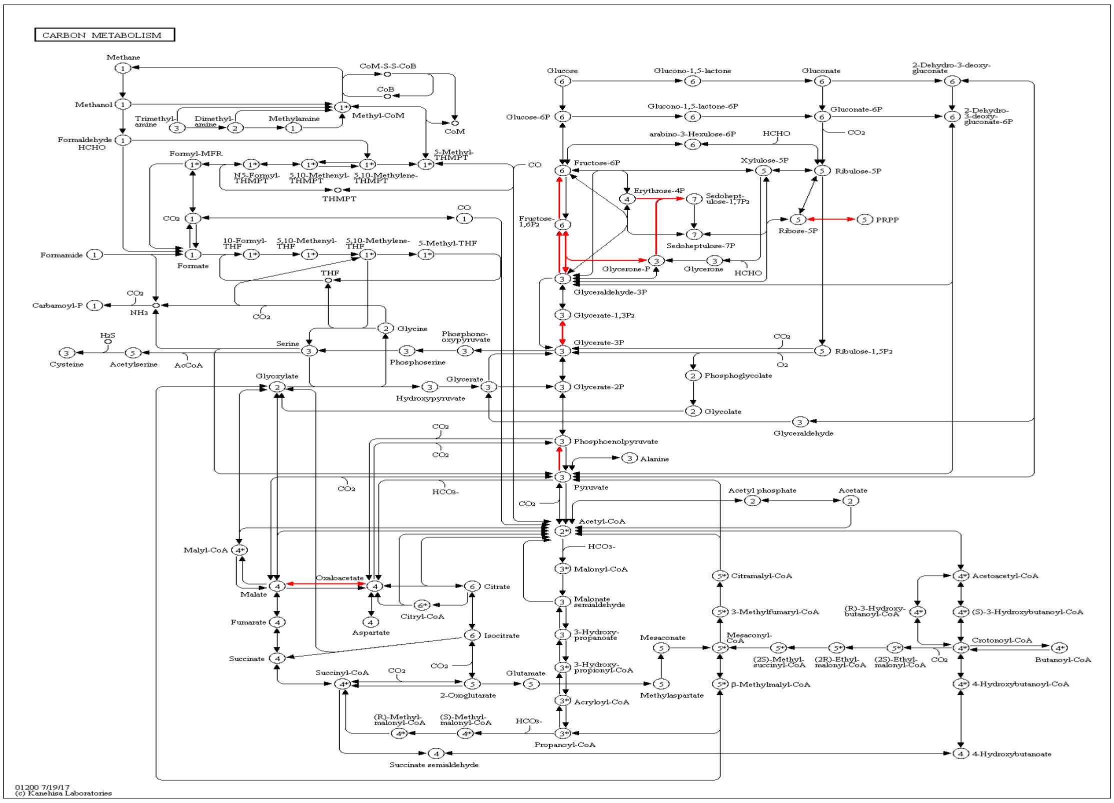
CARBON METABOLISM
Methane
Methanol
Formaldehyde
HCHO
Trimethyl-amine
Dimethyl-amine
Methylamine
Methyl-CoM
CoM-S-S-CoB
CoB
CoM
Formyl-MFR
N5-Formyl-THMPT
5,10-Methenyl-THMPT
5,10-Methylene-THMPT
5-Methyl-THMPT
THMPT
CO
Formamide
Formate
10-Formyl-THF
5,10-Methenyl-THF
5,10-Methylene-THF
5-Methyl-THF
THF
Carbamoyl-P
Glycine
Serine
Cysteine
Acetylserine
AcCoA
Phosphoserine
Glyoxylate
Hydroxypyruvate
Glycerate
Glucose
Glucono-1,5-lactone
Gluconate
2-Dehydro-3-deoxy-gluconate
Glucose-6P
Glucono-1,5-lactone-6P
Gluconate-6P
arabino-3-Hexulose-6P
Fructose-6P
Xylulose-5P
Ribulose-5P
Erythrose-4P
Sedoheptulose-7P
Ribose-5P
PRPP
Fructose-1,6P2
Glycerone-P
Glycerone
Glyceraldehyde-3P
Glycerate-1,3P2
Glycerate-3P
Ribulose-1,5P2
Phosphoglycolate
Glycerate-2P
Glycolate
Glyceraldehyde
Phosphoenolpyruvate
Alanine
Pyruvate
Acetyl phosphate
Acetate
Acetyl-CoA
Malonyl-CoA
Malyl-CoA
Malate
Oxaloacetate
Citrate
Citryl-CoA
Aspartate
Fumarate
Isocitrate
Succinate
Succinyl-CoA
2-Oxoglutarate
Glutamate
Malonate semialdehyde
3-Hydroxy-propanoate
3-Hydroxy-propionyl-CoA
Acryloyl-CoA
Propanoyl-CoA
(R)-Methyl-malonyl-CoA
(S)-Methyl-malonyl-CoA
Succinate semialdehyde
Citramalyl-CoA
3-Methylfumaryl-CoA
Mesaconate
Mesaconyl-CoA
Methylaspartate
β-Methylmalyl-CoA
(2S)-Methyl-succinyl-CoA
(2R)-Ethyl-malonyl-CoA
(2S)-Ethyl-malonyl-CoA
Acetoacetyl-CoA
(R)-3-Hydroxy-butanoyl-CoA
(S)-3-Hydroxybutanoyl-CoA
Crotonoyl-CoA
Butanoyl-CoA
4-Hydroxybutanoyl-CoA
4-Hydroxybutanoate
01200 7/19/17
(c) Kanehisa Laboratories

6B

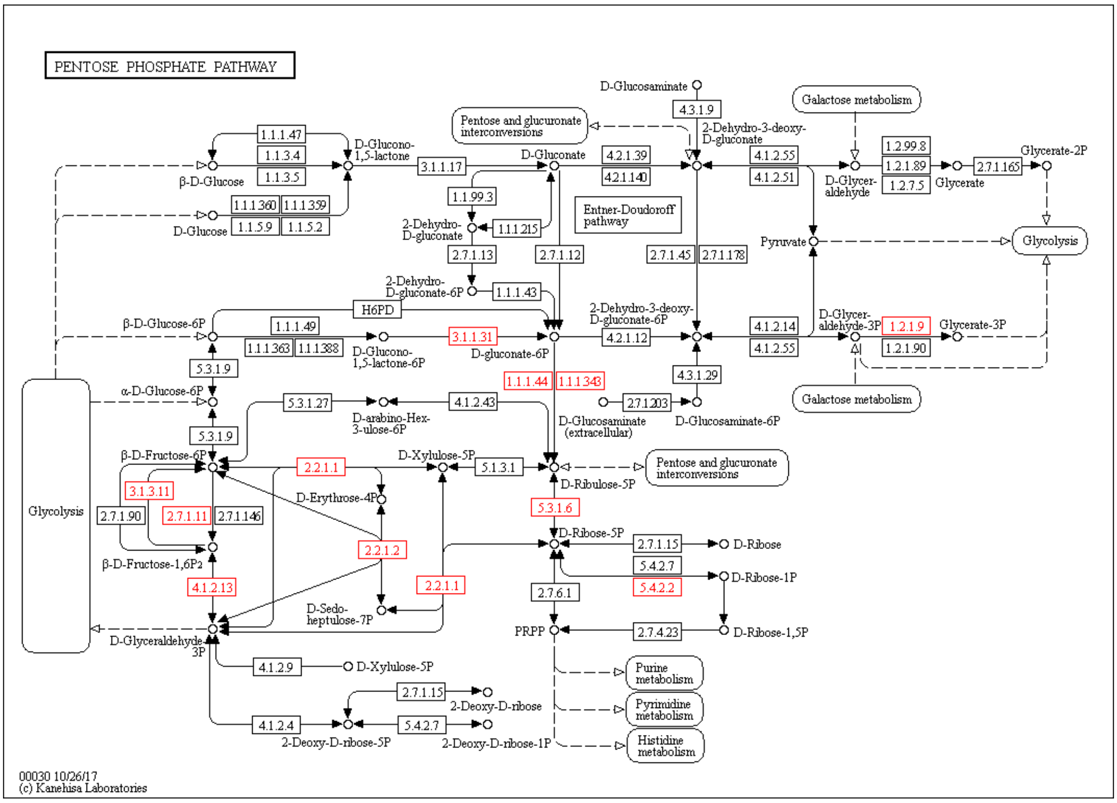
PENTOSE PHOSPHATE PATHWAY
D-Glucosaminate
4.3.1.9
Galactose metabolism
Pentose and glucuronate interconversions
2-Dehydro-3-deoxy-D-gluconate
1.1.1.47
1.1.3.4
1.1.3.5
D-Glucono-1,5-lactone
3.1.1.17
D-Gluconate
4.2.1.39
4.2.1.140
4.1.2.55
4.1.2.51
1.2.99.8
1.2.1.89
1.2.7.5
2.7.1.165
Glycerate-2P
Glycerate
D-Glyceraldehyde
β-D-Glucose
1.1.1.360
1.1.1.359
1.1.5.9
1.1.5.2
D-Glucose
1.1.99.3
2-Dehydro-D-gluconate
1.1.1.215
Entner-Doudoroff pathway
2.7.1.13
2.7.1.12
2.7.1.45
2.7.1.178
Pyruvate
Glycolysis
2-Dehydro-D-gluconate-6P
1.1.1.43
H6PD
2-Dehydro-3-deoxy-D-gluconate-6P
β-D-Glucose-6P
1.1.1.49
1.1.1.363
1.1.1.388
3.1.1.31
4.2.1.12
4.1.2.14
4.1.2.55
D-Glyceraldehyde-3P
1.2.1.9
1.2.1.90
Glycerate-3P
D-Glucono-1,5-lactone-6P
D-gluconate-6P
5.3.1.9
1.1.1.44
1.1.1.343
4.3.1.29
α-D-Glucose-6P
5.3.1.27
D-arabino-Hex-3-ulose-6P
4.1.2.43
2.7.1.203
D-Glucosamine (extracellular)
D-Glucosamine-6P
5.3.1.9
β-D-Fructose-6P
2.2.1.1
D-Xylulose-5P
5.1.3.1
D-Ribulose-5P
Pentose and glucuronate interconversions
3.1.3.11
2.7.1.90
2.7.1.11
2.7.1.146
D-Erythrose-4P
5.3.1.6
D-Ribose-5P
2.7.1.15
D-Ribose
2.2.1.2
β-D-Fructose-1,6P2
5.4.2.7
4.1.2.13
2.2.1.1
2.7.6.1
5.4.2.2
D-Ribose-1P
D-Sedo-heptulose-7P
PRPP
2.7.4.23
D-Ribose-1,5P
D-Glyceraldehyde-3P
4.1.2.9
D-Xylulose-5P
Purine metabolism
2.7.1.15
2-Deoxy-D-ribose
Pyrimidine metabolism
4.1.2.4
5.4.2.7
2-Deoxy-D-ribose-5P
2-Deoxy-D-ribose-1P
Histidine metabolism
00030 10/26/17
(c) Kanehisa Laboratories

6C

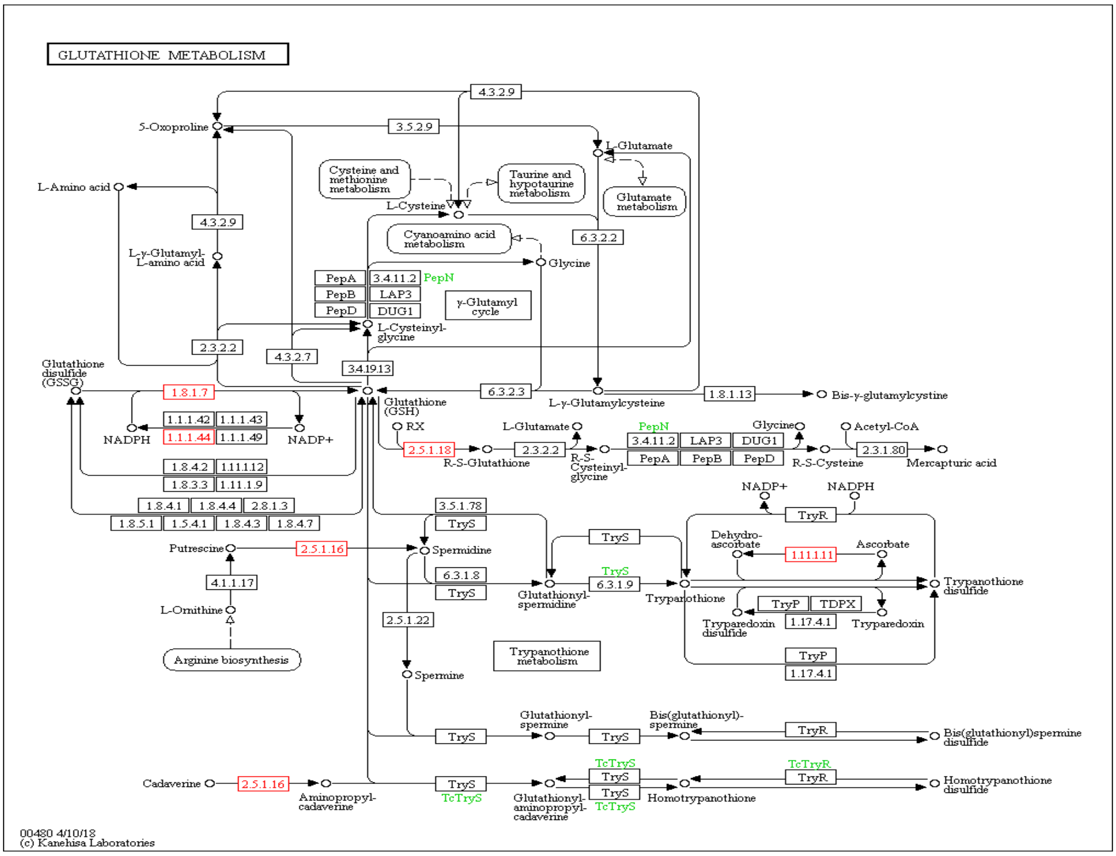

Supplementary Figure 6. Top most significantly enriched KEGG pathways in two maize lines under PEG-induced drought stress conditions. The red boxes are proteins that were differentially expressed under PEG treatment conditions. (**A**) 'Carbon metabolism' pathway was significantly enriched in tolerant line mutant vp16; **(B)** 'Pentose phosphate' pathway was significantly enriched in WD_MD and MC_MD experimental comparisons; and (**C**) 'Glutathione metabolism' pathway was significantly enriched in WD_MD comparison.

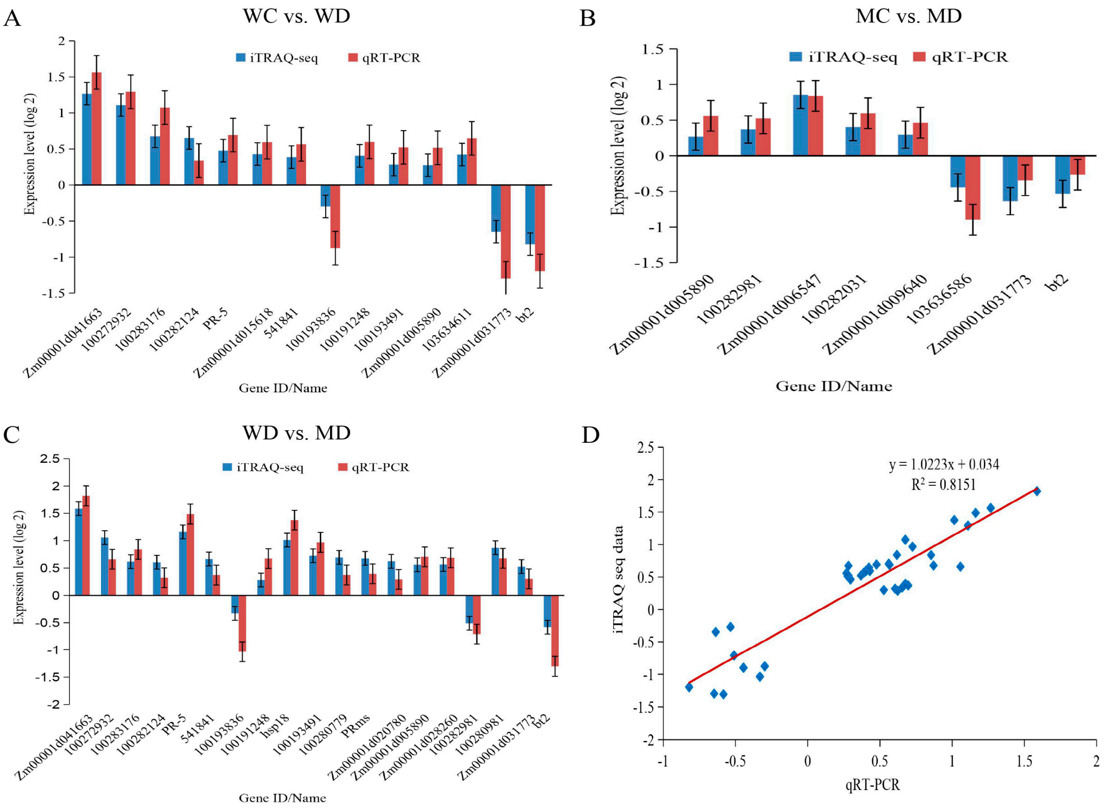

Supplementary Figure 7. Confirmation of iTRAQ-seq results by qRT-PCR analysis. Quantitative RT-PCR analysis of the expression patterns of the maize germinating seeds genes encoded by differentially abundant proteins (DAPs) from different experimental comparisons. (**A**) DAPs in the WC_WD; (**B**) DAPs in MC_MD; and (**C**) DAPs in WD_MD comparisons. (**D**) DAPs expression tendencies in both iTRAQ sequencing data and qRT-PCR experimental results. The x-axis shows the results of qRT-PCR, and the y-axis represents the DAPs relative expression level.
