# Supplementary material for: Comparative Proteomic and Morpho-Physiological Analyses of Maize Wild-Type Vp16 and Mutant vp16 Germinating Seed Responses to PEG-Induced Drought Stress
Source: Int J Mol Sci. 2019 Nov 8;20(22):5586. doi: 10.3390/ijms20225586 (PMC6888951; doi:10.3390/ijms20225586)
Supplement: Supplementary file 1 [file ijms-20-05586-s001.zip › Supplemantery Tables/Supplementary Table 1.doc]

**Supplementary Table1**. Results of drought resistance evaluation of different maize varieties at germination stage

| **Evaluation method** | **Drought tolerance type** | **Varieties** | | | | | |
| --- | --- | --- | --- | --- | --- | --- | --- |
| PI value of germinating stage* | Drought-tolerant type (PI > 0.80) | Denghai3707(0.96) | XianfengT24 (0.95) | Zhengdan958  (0.86) | Mutant vp16 (0.84) | Xianyu335 (0.83) |  |
|  | Relatively drought-tolerant type (PI > 0.40-0.80) | Jinhai5  (0.65) | Pudan4  (0.63) | Liyu16  (0.64) | Zhengdan518  (0.54) |  |  |
|  | General drought-tolerant type (PI > 0.20-0.40) | Anyu12  (0.36) | Shendan16  (0.34) | Gaoyou1  (0.32) | Chengyu5  (0.27) | Danke2123  (0.24) | Jinhai6  (0.23) |
|  | Drought intolerant type(PI < 0. 20) | Liyu6  (0.16) | Taiyu2  (0.14) | Wide-type Vp16 (0.11) | Ludan981  (0.04) | Zhongke4  (0.06) |  |

*The drought-resistance index of seed germination is calculated according to Bouslama and Schapugh (1984) formula. Seed germination drought resistance index = Seed germination index under water stress (PIS) / Seed germination index under well-water (PIC); PI= (1.00) nd2 + (0.75)nd4 + (0.50)nd6 + (0.25)nd8. The figures 2, 4, 6, 8 represent seed germination rate on the 2nd, 4th, 6th and 8th day, respectively.

[Bouslama, M., and Schapugh, W. T. (1984). Stress tolerance in soybeans. 1. Evaluation of 3 screening techniques for heat and drought tolerance. *Crop Sci.* 14, 933-937. doi:10.2135/cropsci1984.0011183X002400050026x]
