# Supplementary material for: Comparative Proteomic and Morpho-Physiological Analyses of Maize Wild-Type Vp16 and Mutant vp16 Germinating Seed Responses to PEG-Induced Drought Stress
Source: Int J Mol Sci. 2019 Nov 8;20(22):5586. doi: 10.3390/ijms20225586 (PMC6888951; doi:10.3390/ijms20225586)
Supplement: Supplementary file 1 [file ijms-20-05586-s001.zip › Supplemantery Tables/Supplementary Table 3 .doc]

**Supplementary Table 3. Drought-responsive maize germinating seeds proteins observed specifically in WD_MD experimental comparison**

| **No.** | **Accession^1^** | **Gene Name/ID^2^** | **Description^3^** | **Cover.^4^** | **Pept. ^5^** | **Fold change^6^** | **p value^7^** | **Expr.^8^** | **Pathways ^9^** |
| --- | --- | --- | --- | --- | --- | --- | --- | --- | --- |
| 1 | B6TXL8 | None | Dehydrin 13 | 20.78 | 2 | 3.03 | 2.92E-02 | Up |  |
| 2 | B7U627 | Zm00001d051420 | DHN2-like protein | 12.46 | 2 | 2.52 | 3.82E-03 | Up |  |
| 3 | B4FVP3 | 100273457 | Ent-cassadiene C2-hydroxylase | 14.46 | 7 | 1.91 | 1.53E-03 | Up |  |
| 4 | C0P948 | Zm00001d024886 | ^10^Uncharacterized protein | 36.59 | 14 | 1.91 | 1.86E-02 | Up |  |
| 5 | A0A1P7ZDI1 | Zm00001d043988 | Chitinase | 8.84 | 2 | 1.82 | 3.41E-03 | Up | Amino sugar and nucleotide sugar metabolism |
| 6 | B4FFV2 | Zm00001d053979 | SWIB/MDM2 domain superfamily protein | 27.61 | 4 | 1.81 | 1.11E-02 | Up |  |
| 7 | C0PMW2 | Zm00001d027387 | Uncharacterized protein | 11.4 | 3 | 1.8 | 1.71E-02 | Up |  |
| 8 | B4FUN3 | Zm00001d047765 | Uncharacterized protein | 50.83 | 4 | 1.74 | 2.41E-02 | Up | Glutathione metabolism |
| 9 | B6STN1 | Zm00001d028692 | IN2-1 protein | 56.07 | 5 | 1.74 | 9.63E-04 | Up | Glutathione metabolism |
| 10 | K7V5N4 | Zm00001d044967 | Eukaryotic aspartyl protease family protein | 8.87 | 4 | 1.7 | 7.37E-03 | Up |  |
| 11 | B6SQA2 | Zm00001d009417 | 60S ribosomal protein L28 | 50.68 | 5 | 1.63 | 1.89E-02 | Up | Ribosome |
| 12 | B6T1F1 | Zm00001d015300 | Ribosomal protein L19 | 28.02 | 2 | 1.62 | 8.94E-03 | Up | Ribosome |
| 13 | B6SLU3 | Zm00001d053776 | Aldehyde dehydrogenase | 27.38 | 2 | 1.62 | 1.78E-02 | Up | Butanoate metabolism/Alanine, aspartate and glutamate metabolism |
| 14 | Q2XX37 | None | Non-specific lipid-transfer protein | 46.22 | 2 | 1.61 | 3.47E-03 | Up |  |
| 15 | B4FFM6 | Zm00001d007687 | Tropinone reductase-like protein | 25.1 | 5 | 1.59 | 8.03E-04 | Up | Tropane, piperidine and pyridine alkaloid biosynthesis |
| 16 | B4FCK9 | Zm00001d022463 | 60S ribosomal protein L22-2 | 47.69 | 2 | 1.58 | 1.58E-02 | Up | Ribosome |
| 17 | B4FML8 | Zm00001d049666 | 40S ribosomal protein S13 | 54.97 | 2 | 1.55 | 1.11E-02 | Up | Ribosome |
| 18 | B4G1Q1 | Zm00001d015375 | 60S ribosomal protein L27a-2 | 41.1 | 3 | 1.55 | 7.00E-04 | Up | Ribosome |
| 19 | Q9FQA3 | Zm00001d020780 | Glutathione transferase GST | 19.82 | 4 | 1.54 | 2.12E-03 | Up | Glutathione metabolism |
| 20 | B6T1H0 | Zm00001d022180 | 40S ribosomal protein S6 | 50.6 | 2 | 1.54 | 2.69E-03 | Up | Ribosome |
| 21 | A0A1D6N0K3 | Zm00001d042022 | Peroxidase | 34.07 | 10 | 1.53 | 1.03E-02 | Up | Phenylpropanoid biosynthesis |
| 22 | B6TK66 | Zm00001d027832 | Embryonic protein DC-8 | 31.56 | 11 | 1.52 | 1.28E-02 | Up |  |
| 23 | B6SPI9 | Zm00001d052718 | 60S ribosomal protein L38 | 53.62 | 5 | 1.51 | 9.71E-03 | Up | Ribosome |
| 24 | B4G144 | Zm00001d011349 | Uncharacterized protein | 18.51 | 2 | 1.48 | 2.83E-02 | Up |  |
| 25 | K7VEA3 | Zm00001d047567 | Embryonic cell protein 63 | 18.87 | 5 | 1.48 | 2.13E-02 | Up |  |
| 26 | B6UHH1 | Zm00001d023387 | Uncharacterized protein | 22.92 | 3 | 1.47 | 6.88E-03 | Up |  |
| 27 | B6TE40 | Zm00001d047500 | 60S ribosomal protein L22-2 | 25.2 | 2 | 1.46 | 1.57E-03 | Up | Ribosome |
| 28 | B6SLF6 | Zm00001d052958 | Antimicrobial peptide MBP-1 | 11.96 | 6 | 1.46 | 2.76E-02 | Up |  |
| 29 | A0A1D6IUF2 | Zm00001d023617 | Laccase | 3.93 | 2 | 1.45 | 1.09E-02 | Up |  |
| 30 | C0P493 | Zm00001d028655 | Uncharacterized protein | 10.44 | 2 | 1.45 | 2.94E-02 | Up | mRNA surveillance pathway/RNA transport |
| 31 | B6TB21 | Zm00001d001856 | Anamorsin homolog | 42.97 | 8 | 1.44 | 6.85E-03 | Up |  |
| 32 | B6TUI0 | Zm00001d006796 | Estradiol 17-beta-dehydrogenase 8 | 12.09 | 2 | 1.43 | 4.99E-02 | Up | Biotin metabolism/Monobactam biosynthesis/Biosynthesis of unsaturated fatty acids |
| 33 | A0A1D6E5Q7 | Zm00001d002960 | Kunitz trypsin inhibitor 1 | 25.66 | 4 | 1.42 | 4.43E-03 | Up |  |
| 34 | A0A1D6JKW8 | Zm00001d027332 | Nonspecific lipid-transfer protein | 31.63 | 4 | 1.42 | 8.53E-04 | Up |  |
| 35 | B8A068 | Zm00001d024754 | S-adenosylmethionine synthase | 44.08 | 6 | 1.42 | 3.17E-02 | Up |  |
| 36 | B6T7A1 | Zm00001d018485 | Voltage-gated potassium channel beta subunit | 15.55 | 5 | 1.42 | 3.30E-03 | Up |  |
| 37 | B6SYW0 | Zm00001d034609 | 40S ribosomal protein S26 | 50.76 | 2 | 1.42 | 1.00E-03 | Up | Ribosome |
| 38 | A0A1D6N1C9 | Zm00001d042088 | Uridine 5'-monophosphate synthase | 23.56 | 7 | 1.42 | 2.58E-03 | Up | Pyrimidine metabolism |
| 39 | A0A1D6MTY1 | Zm00001d041079 | Quinolinate synthase chloroplastic | 13.41 | 3 | 1.4 | 1.83E-02 | Up |  |
| 40 | B6UCK4 | Zm00001d020343 | Catalytic/ ligase | 12.33 | 8 | 1.4 | 7.22E-04 | Up |  |
| 41 | Q6JBP8 | None | Chitinase | 54.84 | 11 | 1.39 | 3.10E-02 | Up |  |
| 42 | A0A1D6IZ49 | Zm00001d024414 | Seed maturation protein | 41.51 | 4 | 1.38 | 3.71E-02 | Up |  |
| 43 | B4G1A3 | Zm00001d050323 | Uncharacterized protein | 6.04 | 3 | 1.38 | 1.02E-02 | Up |  |
| 44 | A0A1D6IV85 | Zm00001d023734 | Glycine-rich protein2 | 15.11 | 2 | 1.38 | 2.97E-03 | Up | Spliceosome |
| 45 | B7ZXM4 | Zm00001d040667 | Uncharacterized protein | 8.24 | 2 | 1.37 | 3.84E-02 | Up | Amino sugar and nucleotide sugar metabolism |
| 46 | C0PH34 | Zm00001d017047 | 60S ribosomal protein L6 | 42.92 | 3 | 1.37 | 2.58E-03 | Up | Ribosome |
| 47 | B4FID1 | Zm00001d037111 | 40S ribosomal protein S17-1 | 62.68 | 2 | 1.37 | 1.45E-02 | Up | Ribosome |
| 48 | B6UDY2 | Zm00001d049815 | 60S ribosomal protein L17 | 53.8 | 5 | 1.36 | 3.57E-04 | Up | Ribosome |
| 49 | Q7SIC9 | Zm00001d045451 | Transketolase, chloroplastic | 33.48 | 18 | 1.36 | 1.59E-03 | Up | Zeatin biosynthesis/Carbon fixation in photosynthetic organisms/Pentose phosphate pathway |
| 50 | B4FS90 | Zm00001d026577 | Cysteine protease 1 | 12.77 | 5 | 1.36 | 2.42E-02 | Up |  |
| 51 | B4FDH0 | Zm00001d045043 | Uncharacterized protein | 16.99 | 3 | 1.35 | 3.19E-03 | Up |  |
| 52 | K7TFB6 | Zm00001d023664 | ABA-responsive protein | 46.69 | 10 | 1.34 | 1.24E-03 | Up |  |
| 53 | B8A1K8 | Zm00001d034760 | Ferredoxin | 42.86 | 2 | 1.34 | 4.18E-03 | Up | Photosynthesis |
| 54 | B4G1Y5 | Zm00001d015956 | NAD(P)-binding Rossmann-fold superfamily protein | 36.75 | 9 | 1.34 | 1.41E-03 | Up |  |
| 55 | B6TF33 | Zm00001d034037 | Circumsporozoite protein | 13.31 | 3 | 1.34 | 4.30E-02 | Up |  |
| 56 | B4FN24 | Zm00001d011581 | Peroxiredoxin-2B | 58.64 | 6 | 1.34 | 8.21E-03 | Up |  |
| 57 | B6THU9 | Zm00001d014467 | Peroxidase | 15.55 | 4 | 1.34 | 4.00E-04 | Up | Phenylpropanoid biosynthesis |
| 58 | B4FRR1 | Zm00001d018197 | Soluble inorganic pyrophosphatase 6 chloroplastic | 12.15 | 3 | 1.34 | 3.17E-02 | Up | Oxidative phosphorylation |
| 59 | Q9SYS1 | Zm00001d021702 | Beta-amylase | 47.13 | 18 | 1.33 | 2.41E-03 | Up |  |
| 60 | B4FVJ0 | Zm00001d007824 | 60S ribosomal protein L4-1 | 68.81 | 7 | 1.33 | 3.38E-02 | Up | Ribosome |
| 61 | C0P4M0 | Zm00001d049954 | Monodehydroascorbate reductase 1 peroxisomal | 27.25 | 10 | 1.33 | 3.74E-04 | Up | Ascorbate and aldarate metabolism |
| 62 | A0A1D6LTL9 | Zm00001d037059 | Alpha-glucan water dikinase 1 chloroplastic | 6.13 | 8 | 1.33 | 6.90E-04 | Up |  |
| 63 | Q2XX18 | None | Non-specific lipid-transfer protein (Fragment) | 60.55 | 4 | 1.33 | 1.42E-02 | Up |  |
| 64 | B6TNR8 | Zm00001d019147 | 40S ribosomal protein S2 | 58.06 | 4 | 1.33 | 3.06E-03 | Up | Ribosome |
| 65 | K7TQJ7 | Zm00001d025777 | Eukaryotic translation initiation factor isoform 4G-2 | 25.86 | 8 | 1.33 | 2.53E-02 | Up |  |
| 66 | B6UE05 | Zm00001d031640 | 60S ribosomal protein L7a | 43.92 | 4 | 1.33 | 2.62E-03 | Up | Ribosome |
| 67 | B6U2S6 | Zm00001d023213 | Transposon protein | 9.87 | 4 | 1.33 | 2.69E-02 | Up |  |
| 68 | B4FBC8 | Zm00001d052335 | Peroxidase | 20 | 5 | 1.32 | 2.09E-02 | Up | Phenylpropanoid biosynthesis |
| 69 | A0A1D6JYA0 | Zm00001d028653 | dTDP-glucose dehydratase homolog csu219 | 41.5 | 2 | 1.32 | 1.95E-02 | Up | Amino sugar and nucleotide sugar metabolism |
| 70 | B6SGY5 | Zm00001d012824 | RNA binding protein | 29.61 | 4 | 1.32 | 1.55E-02 | Up |  |
| 71 | C0PA71 | Zm00001d013667 | Uncharacterized protein | 16.75 | 2 | 1.32 | 4.18E-03 | Up |  |
| 72 | B6TZD1 | Zm00001d049042 | Methylthioribose-1-phosphate isomerase | 46.05 | 13 | 1.31 | 1.65E-03 | Up | Cysteine and methionine metabolism |
| 73 | K7URC3 | Zm00001d049399 | Putative nucleolin-like family protein | 9.71 | 6 | 1.31 | 5.93E-03 | Up |  |
| 74 | B6TDT6 | Zm00001d026573 | Methylthioribose kinase | 36.94 | 14 | 1.31 | 2.47E-03 | Up | Cysteine and methionine metabolism |
| 75 | C0PKN5 | Zm00001d003188 | Endochitinase EP3 | 7.45 | 2 | 1.31 | 1.11E-03 | Up | Amino sugar and nucleotide sugar metabolism |
| 76 | Q9FQC9 | Zm00001d027542 | Glutathione S-transferase GST 10 | 12.06 | 2 | 1.31 | 5.67E-03 | Up | Glutathione metabolism |
| 77 | C0PBE2 | Zm00001d035032 | Uncharacterized protein | 28.13 | 5 | 1.31 | 2.94E-03 | Up |  |
| 78 | B6SU66 | Zm00001d053715 | Glycosyltransferase | 14.6 | 5 | 1.3 | 3.78E-02 | Up |  |
| 79 | B6TBZ1 | Zm00001d004413 | Cysteine synthase | 11.26 | 3 | 1.3 | 1.07E-02 | Up | Cysteine and methionine metabolism/Cyanoamino acid metabolism/Sulfur metabolism |
| 80 | A0A1D6GEK0 | Zm00001d012978 | 40S ribosomal protein S23-2 | 42.48 | 7 | 1.3 | 1.51E-03 | Up | Ribosome |
| 81 | Q8LK07 | Zm00001d002601 | Histone H1 | 25.51 | 5 | 1.3 | 1.54E-02 | Up |  |
| 82 | K7V5I3 | Zm00001d044906 | 12-oxo-phytodienoic acid reductase2 | 11.53 | 3 | 1.3 | 5.14E-03 | Up | alpha-Linolenic acid metabolism |
| 83 | Q7FS90 | Zm00001d038449 | Putative aldose reductase-related protein | 12.39 | 2 | 1.29 | 4.83E-02 | Up | Glycerolipid metabolism/Pentose and glucuronate interconversions/Glycolysis / Gluconeogenesis |
| 84 | B6U9D5 | Zm00001d034937 | Glutathione S-transferase T1 | 6.43 | 2 | 1.29 | 2.69E-02 | Up | Glutathione metabolism |
| 85 | B6T2J0 | Zm00001d038653 | Thioredoxin | 41.41 | 6 | 1.28 | 1.56E-02 | Up |  |
| 86 | B6TMB2 | Zm00001d040766 | PDIL2-2-Zea mays protein disulfide isomerase | 51.23 | 15 | 1.28 | 2.28E-02 | Up | Protein processing in endoplasmic reticulum |
| 87 | A0A1D6EIR5 | Zm00001d005019 | Acyl-CoA synthetase long-chain family member 3 | 9.92 | 5 | 1.28 | 1.07E-04 | Up | Fatty acid degradation/Fatty acid biosynthesis/Peroxisome |
| 88 | B4FPP1 | Zm00001d044497 | Thioredoxin family Trp26 | 18.14 | 3 | 1.28 | 1.18E-03 | Up |  |
| 89 | B4FXZ3 | Zm00001d003081 | Grx_C2.2-glutaredoxin subgroup I | 70.8 | 6 | 1.28 | 4.28E-02 | Up |  |
| 90 | B6TS38 | Zm00001d030701 | Ribose-5-phosphate isomerase | 19.77 | 3 | 1.28 | 7.14E-03 | Up | Carbon fixation in photosynthetic organisms/Pentose phosphate pathway |
| 91 | B4G0K5 | Zm00001d028998 | Uncharacterized protein | 80.23 | 17 | 1.28 | 8.52E-03 | Up |  |
| 92 | B4FN17 | Zm00001d011125 | USP family protein | 69.46 | 3 | 1.28 | 7.01E-03 | Up |  |
| 93 | A0A1D6J0S0 | Zm00001d024633 | Monodehydroascorbate reductase 5 mitochondrial | 11.89 | 5 | 1.27 | 1.34E-02 | Up | Ascorbate and aldarate metabolism |
| 94 | B6TLM5 | Zm00001d043795 | Glutathione S-transferase GSTU6 | 35.34 | 8 | 1.27 | 1.02E-02 | Up | Ascorbate and aldarate metabolism/Glutathione metabolism |
| 95 | B4FT46 | Zm00001d028693 | Safener induced1 | 36.63 | 7 | 1.26 | 1.99E-03 | Up | Glutathione metabolism |
| 96 | A0A1D6KTS3 | Zm00001d032748 | Nucleolin 2 | 5.6 | 2 | 1.26 | 4.83E-02 | Up |  |
| 97 | B4FKX0 | Zm00001d012667 | Uncharacterized protein | 10.34 | 3 | 1.26 | 3.37E-02 | Up |  |
| 98 | B7ZXZ2 | Zm00001d038365 | Aldo-keto reductase family 4 member C9 | 27.65 | 5 | 1.26 | 1.72E-02 | Up | Glycerolipid metabolism/Pentose and glucuronate interconversions/Glycolysis / Gluconeogenesis |
| 99 | B6T2K5 | Zm00001d004052 | 60S ribosomal protein L35 | 33.33 | 3 | 1.26 | 1.32E-02 | Up | Ribosome |
| 100 | A0A1D6QLL5 | Zm00001d053028 | Protein TPLATE | 1.73 | 2 | 1.26 | 2.25E-02 | Up |  |
| 101 | A0A1D6L8E2 | Zm00001d034528 | Cytochrome b-c1 complex subunit 7 | 26.02 | 3 | 1.26 | 3.40E-02 | Up | Oxidative phosphorylation |
| 102 | B8A1V8 | Zm00001d027519 | Uncharacterized protein | 18.2 | 7 | 1.25 | 1.66E-02 | Up | alpha-Linolenic acid metabolism |
| 103 | B6T339 | Zm00001d008841 | 17.5 kDa class II heat shock protein | 36.59 | 3 | 1.25 | 1.51E-02 | Up | Protein processing in endoplasmic reticulum |
| 104 | B6T769 | Zm00001d021061 | 60S ribosomal protein L7a | 49.22 | 6 | 1.25 | 4.91E-02 | Up | Ribosome |
| 105 | Q9FER8 | Zm00001d032239 | HMGI/Y protein | 18.85 | 3 | 1.25 | 2.41E-04 | Up |  |
| 106 | A0A096PQR7 | Zm00001d028282 | Cytochrome P450 CYP74A19 | 48.96 | 7 | 1.25 | 7.16E-03 | Up | alpha-Linolenic acid metabolism |
| 107 | A0A1D6PIA7 | Zm00001d048176 | Vicilin-like seed storage protein | 31.96 | 17 | 1.25 | 3.02E-02 | Up |  |
| 108 | B4FHM5 | Zm00001d004009 | 60S ribosomal protein L37a | 45.65 | 6 | 1.24 | 5.42E-03 | Up | Ribosome |
| 109 | A0A1D6KR83 | Zm00001d032519 | Uncharacterized protein | 51.19 | 17 | 1.24 | 3.38E-02 | Up |  |
| 110 | A0A1D6JMZ7 | Zm00001d027557 | Glutathione transferase31 | 18.75 | 3 | 1.24 | 3.59E-02 | Up | Glutathione metabolism |
| 111 | C0PGI9 | Zm00001d021881 | Uncharacterized protein | 8.33 | 2 | 1.24 | 1.64E-03 | Up |  |
| 112 | A0A1D6H4X4 | Zm00001d015992 | Uncharacterized protein | 41.95 | 7 | 1.24 | 5.63E-03 | Up | Fatty acid degradation/alpha-Linolenic acid metabolism |
| 113 | A0A1D6QR90 | Zm00001d053658 | Ubiquitin family protein | 16.11 | 4 | 1.24 | 6.66E-03 | Up |  |
| 114 | B4FS99 | Zm00001d031825 | ATP synthase subunit d, mitochondrial | 60.59 | 10 | 1.24 | 1.46E-04 | Up | Oxidative phosphorylation |
| 115 | B6SST9 | Zm00001d006439 | ATP synthase delta chain | 48.28 | 3 | 1.24 | 1.55E-02 | Up | Oxidative phosphorylation |
| 116 | A0A1D6LZ57 | Zm00001d037573 | USP family protein | 79.04 | 4 | 1.24 | 9.60E-03 | Up |  |
| 117 | B6SJE6 | Zm00001d027500 | Flower-specific gamma-thionin | 36.92 | 3 | 1.24 | 6.62E-03 | Up |  |
| 118 | B8A377 | Zm00001d031136 | Cysteine synthase | 64.62 | 16 | 1.24 | 8.41E-03 | Up | Cysteine and methionine metabolism/Sulfur metabolism |
| 119 | B6TF12 | Zm00001d034768 | Glycine-rich RNA-binding protein 8 | 21.72 | 4 | 1.24 | 4.53E-03 | Up | Spliceosome |
| 120 | B6T7B2 | Zm00001d041760 | 40S ribosomal protein S9 | 61.34 | 6 | 1.24 | 9.95E-03 | Up |  |
| 121 | Q9FQB7 | Zm00001d024963 | Glutathione S-transferase GST 22 (Fragment) | 37.55 | 7 | 1.24 | 4.43E-03 | Up | Glutathione metabolism |
| 122 | B4FCK0 | Zm00001d012610 | USP family protein | 41.8 | 3 | 1.24 | 3.45E-05 | Up |  |
| 123 | A0A1D6Q167 | Zm00001d050336 | Methylcrotonoyl-CoA carboxylase beta chain mitochondrial | 49.81 | 19 | 1.24 | 4.80E-02 | Up | Valine, leucine and isoleucine degradation |
| 124 | A0A1D6LAE8 | Zm00001d034733 | Galactokinase | 12.97 | 4 | 1.23 | 3.65E-02 | Up |  |
| 125 | A0A1D6J4J1 | Zm00001d025059 | Germin-like protein subfamily 1 member 8 | 22.03 | 5 | 1.23 | 2.46E-04 | Up |  |
| 126 | B6T8Q2 | Zm00001d013755 | Uncharacterized protein | 23.24 | 5 | 1.23 | 2.57E-02 | Up |  |
| 127 | C0P4E9 | Zm00001d042104 | Uncharacterized protein | 23.66 | 5 | 1.22 | 1.51E-02 | Up | Glutathione metabolism |
| 128 | A0A1D6LA70 | Zm00001d034701 | Protein DEFECTIVE IN EXINE FORMATION 1 | 4.78 | 3 | 1.22 | 9.82E-03 | Up |  |
| 129 | B4FRL5 | Zm00001d023223 | Ferritin | 13.49 | 2 | 1.22 | 2.45E-03 | Up |  |
| 130 | B4FKP0 | Zm00001d026664 | NDR1/HIN1-like 1 | 12.67 | 3 | 1.22 | 1.94E-02 | Up |  |
| 131 | B6T5Q6 | Zm00001d004176 | Uncharacterized protein | 14.76 | 3 | 1.22 | 1.35E-02 | Up |  |
| 132 | B4F855 | Zm00001d013838 | Tetratricopeptide repeat (TPR)-like superfamily protein | 4.95 | 3 | 1.22 | 1.73E-02 | Up |  |
| 133 | B6U9L8 | Zm00001d012909 | Epoxide hydrolase 2 | 16.67 | 4 | 1.22 | 4.34E-03 | Up |  |
| 134 | B6T5R1 | Zm00001d010504 | Ran-binding protein 1 | 28.05 | 3 | 1.22 | 1.12E-02 | Up |  |
| 135 | B4FLP7 | Zm00001d020139 | Cysteine desulfurase mitochondrial | 11.31 | 4 | 1.22 | 1.11E-03 | Up | Thiamine metabolism/Sulfur relay system |
| 136 | B6SI37 | Zm00001d037988 | Embryo specific protein1 | 68.13 | 7 | 1.22 | 3.63E-02 | Up |  |
| 137 | B6SHZ1 | Zm00001d008387 | 40S ribosomal protein S5 | 43.22 | 4 | 1.22 | 3.82E-03 | Up | Ribosome |
| 138 | B7ZYH5 | Zm00001d048669 | Uncharacterized protein | 6.05 | 4 | 1.21 | 3.54E-02 | Up | Cysteine and methionine metabolism |
| 139 | B4FU52 | Zm00001d012837 | Histone H2A | 36.67 | 3 | 1.21 | 1.02E-02 | Up |  |
| 140 | C4JBS8 | Zm00001d049641 | Glyceraldehyde-3-phosphate dehydrogenase | 83.68 | 8 | 1.21 | 1.95E-02 | Up | Carbon fixation in photosynthetic organisms/ |
| 141 | B4FPT8 | Zm00001d033128 | 60S ribosomal protein L10-3 | 58.18 | 14 | 1.21 | 4.69E-04 | Up | Ribosome |
| 142 | A0A1D6PUL7 | Zm00001d049409 | Aconitate hydratase | 53.38 | 26 | 1.21 | 7.29E-04 | Up | Glyoxylate and dicarboxylate metabolism/Citrate cycle (TCA cycle) |
| 143 | B8A3K0 | Zm00001d018809 | Glutathione transferase6 | 22.97 | 5 | 1.21 | 3.22E-04 | Up | Glutathione metabolism |
| 144 | C0HJ94 | Zm00001d046949 | Uncharacterized protein | 9.95 | 2 | 1.21 | 3.32E-02 | Up |  |
| 145 | C4JA45 | Zm00001d012161 | 60S ribosomal protein L5-1 homolog b | 52.98 | 6 | 1.21 | 9.14E-03 | Up | Ribosome |
| 146 | B4FQG7 | Zm00001d052767 | Uncharacterized protein | 6.35 | 3 | 1.21 | 3.94E-02 | Up |  |
| 147 | Q9SAZ6 | Zm00001d016166 | Phosphoenolpyruvate carboxylase | 44.38 | 7 | 1.21 | 2.21E-02 | Up | Carbon fixation in photosynthetic organisms/Pyruvate metabolism |
| 148 | B6TX09 | Zm00001d010172 | Fructose-1,6-bisphosphatase, cytosolic | 20.47 | 6 | 1.21 | 2.12E-02 | Up | Glycolysis / Gluconeogenesis/Carbon fixation in photosynthetic organisms |
| 149 | B6TNY2 | Zm00001d038532 | Lipid binding protein | 18.44 | 2 | 1.2 | 1.66E-04 | Up |  |
| 150 | A0A1D6FSD2 | Zm00001d010614 | 40S ribosomal protein S26 | 37.14 | 2 | 1.2 | 2.10E-02 | Up | Ribosome |
| 151 | B6T8U5 | Zm00001d038616 | ER6 protein | 29.23 | 3 | 1.2 | 1.88E-02 | Up |  |
| 152 | K7V686 | Zm00001d011100 | Eukaryotic translation initiation factor 3 subunit F | 34.15 | 3 | 0.83 | 2.71E-02 | Down |  |
| 153 | B6TRV3 | Zm00001d005238 | Eukaryotic translation initiation factor 3 subunit E | 38.3 | 8 | 0.83 | 1.11E-02 | Down | RNA transport |
| 154 | Q41834 | Zm00001d019648 | Nucleic acid-binding protein | 17.49 | 4 | 0.83 | 2.72E-05 | Down |  |
| 155 | B4FS59 | Zm00001d040191 | Zinc ion binding | 13.46 | 4 | 0.83 | 5.29E-04 | Down |  |
| 156 | A0A1D6PDS3 | Zm00001d047828 | Protein TOC75-3 chloroplastic | 48.91 | 7 | 0.83 | 1.97E-02 | Down |  |
| 157 | B4FPM2 | Zm00001d034824 | Aldose 1-epimerase | 16.67 | 5 | 0.83 | 1.12E-03 | Down | Glycolysis / Gluconeogenesis/Galactose metabolism |
| 158 | B4FAD9 | Zm00001d021421 | UDP-glucose pyrophosphorylase2 | 79.7 | 10 | 0.83 | 1.19E-02 | Down | Pentose and glucuronate interconversions |
| 159 | B4F8H6 | Zm00001d009866 | Signal peptidase I | 28.33 | 5 | 0.83 | 3.19E-02 | Down | Protein export |
| 160 | B6UBL0 | Zm00001d052061 | Protein CYPRO4 | 28.5 | 12 | 0.83 | 1.40E-02 | Down |  |
| 161 | K7WAM2 | Zm00001d046581 | Isoleucine--tRNA ligase cytoplasmic | 28.67 | 9 | 0.83 | 7.97E-03 | Down | Aminoacyl-tRNA biosynthesis |
| 162 | B6TXJ8 | Zm00001d035899 | Glycoside hydrolase, family 28 | 10.93 | 3 | 0.83 | 9.49E-05 | Down |  |
| 163 | P93804 | Zm00001d033746 | Phosphoglucomutase, cytoplasmic 1 | 67.58 | 8 | 0.82 | 1.72E-02 | Down | alactose metabolism/Pentose phosphate pathway/Starch and sucrose metabolism |
| 164 | A0A1D6LA97 | Zm00001d034713 | Anthranilate synthase alpha subunit 2 chloroplastic | 29.29 | 12 | 0.82 | 3.95E-03 | Down | Monobactam biosynthesis/Phenylalanine, tyrosine and tryptophan biosynthesis |
| 165 | B6T6B3 | Zm00001d008757 | Eukaryotic translation initiation factor 3 subunit M | 58.31 | 19 | 0.82 | 4.50E-04 | Down |  |
| 166 | C0P4U5 | Zm00001d002953 | Uncharacterized protein | 16.72 | 5 | 0.82 | 4.25E-04 | Down |  |
| 167 | C0PIX5 | Zm00001d045340 | Uncharacterized protein | 22.64 | 8 | 0.82 | 6.86E-03 | Down | Cysteine and methionine metabolism/Sulfur metabolism |
| 168 | A0A1D6LYS5 | Zm00001d037546 | Uncharacterized protein O | 11.97 | 3 | 0.82 | 1.90E-03 | Down |  |
| 169 | P93805 | Zm00001d033746 | Phosphoglucomutase, cytoplasmic 2 | 67.58 | 6 | 0.82 | 2.68E-02 | Down | Galactose metabolism/Purine metabolism/Pentose phosphate pathway |
| 170 | A0A1D6I6H5 | Zm00001d020827 | Heat shock protein 90-5 chloroplastic | 31.62 | 7 | 0.82 | 7.71E-03 | Down |  |
| 171 | B6TX10 | Zm00001d049187 | 6-phosphogluconate dehydrogenase, decarboxylating | 39.84 | 11 | 0.82 | 2.33E-02 | Down | Glutathione metabolism/Pentose phosphate pathway |
| 172 | G4XT43 | Zm00001d007765 | Prolyl 4-hydroxylase 4 | 22.63 | 5 | 0.82 | 5.87E-03 | Down | Arginine and proline metabolism |
| 173 | B6TF54 | Zm00001d012674 | Chorismate mutase | 32.69 | 2 | 0.82 | 1.85E-02 | Down | Phenylalanine, tyrosine and tryptophan biosynthesis |
| 174 | B4FIK0 | Zm00001d039066 | Glucose-6-phosphate 1-epimerase | 17.61 | 5 | 0.82 | 2.68E-02 | Down | Glycolysis / Gluconeogenesis |
| 175 | A0A1D6FH31 | Zm00001d008993 | DUF4378 domain protein | 8.87 | 2 | 0.82 | 1.17E-02 | Down | Glyoxylate and dicarboxylate metabolism/Glycine, serine and threonine metabolism |
| 176 | A0A1D6GZY9 | Zm00001d015208 | Cleavage and polyadenylation specificity factor (CPSF) A subunit protein | 21.97 | 22 | 0.82 | 1.38E-03 | Down | Spliceosome |
| 177 | B6TXL9 | Zm00001d008651 | Pyruvate decarboxylase isozyme 1 | 41.82 | 13 | 0.82 | 3.15E-03 | Down | Glycolysis / Gluconeogenesis |
| 178 | K7UGR2 | Zm00001d054089 | Putative TCP-1/cpn60 chaperonin family protein isoform 1 | 62.98 | 21 | 0.82 | 7.02E-04 | Down | RNA degradation |
| 179 | B4FHF0 | Zm00001d044277 | Elongation factor Ts, mitochondrial | 10.03 | 4 | 0.82 | 1.05E-03 | Down |  |
| 180 | B4FTH5 | Zm00001d024379 | Xyloglucan endotransglucosylase/hydrolase | 19.23 | 4 | 0.82 | 6.27E-03 | Down |  |
| 181 | B4F999 | Zm00001d032504 | Uncharacterized protein | 5.65 | 3 | 0.82 | 3.75E-02 | Down |  |
| 182 | B6SMQ5 | Zm00001d012407 | Triose phosphate isomerase5 | 55.47 | 10 | 0.82 | 1.66E-02 | Down | Fructose and mannose metabolism/Inositol phosphate metabolism |
| 183 | A0A1D6IQM5 | Zm00001d023222 | Ubiquitin-activating enzyme E1 2 | 33.89 | 3 | 0.82 | 4.23E-02 | Down | Ubiquitin mediated proteolysis |
| 184 | A0A1D6QSB0 | Zm00001d053798 | Glyceraldehyde-3-phosphate dehydrogenase | 48.38 | 2 | 0.82 | 2.50E-02 | Down | Carbon fixation in photosynthetic organisms/Glycolysis / Gluconeogenesis |
| 185 | A0A1D6HSR3 | Zm00001d018857 | Putative carboxylesterase 15 | 34.15 | 8 | 0.81 | 1.15E-02 | Down |  |
| 186 | A0A1D6LTJ4 | Zm00001d037052 | Ubiquitin-conjugating enzyme E2 11 | 29.69 | 2 | 0.81 | 4.48E-02 | Down |  |
| 187 | B6T940 | Zm00001d042478 | VTC2 | 20.34 | 6 | 0.81 | 2.57E-02 | Down | Ascorbate and aldarate metabolism |
| 188 | A0A1D6LSM6 | Zm00001d036949 | Serine/threonine-protein phosphatase | 28.76 | 4 | 0.81 | 1.63E-02 | Down | Autophagy - other eukaryotes/mRNA surveillance pathway |
| 189 | B6T402 | Zm00001d033906 | Endoplasmic oxidoreductin-1 | 20.72 | 7 | 0.81 | 2.57E-03 | Down | Protein processing in endoplasmic reticulum |
| 190 | A0A1D6PTQ0 | Zm00001d049297 | Chromophore lyase CRL chloroplastic | 10.55 | 2 | 0.81 | 6.21E-03 | Down |  |
| 191 | P49353 | Zm00001d009431 | Farnesyl pyrophosphate synthase | 49.43 | 12 | 0.81 | 8.92E-03 | Down | Terpenoid backbone biosynthesis/Starch and sucrose metabolism |
| 192 | A0A1D6Q540 | Zm00001d051112 | RAN GTPase-activating protein 2 | 24.03 | 2 | 0.81 | 3.36E-02 | Down | RNA transport |
| 193 | K7U1S1 | Zm00001d052079 | Lysine-ketoglutarate reductase/saccharopine dehydrogenase1 | 24.27 | 21 | 0.81 | 2.03E-02 | Down | Lysine degradation |
| 194 | B4F9C8 | Zm00001d052561 | Uncharacterized protein | 13.67 | 2 | 0.81 | 4.12E-02 | Down | RNA degradation |
| 195 | A0A1D6I097 | Zm00001d019757 | Beta-amylase | 5.37 | 3 | 0.81 | 2.73E-02 | Down |  |
| 196 | A0A1D6HUP0 | Zm00001d019040 | D-2-hydroxyglutarate dehydrogenase mitochondrial | 6.63 | 3 | 0.81 | 2.04E-03 | Down |  |
| 197 | A0A1D6KCZ2 | Zm00001d030557 | Alanine aminotransferase 2 mitochondrial | 62.86 | 11 | 0.81 | 8.64E-04 | Down | Carbon fixation in photosynthetic organisms/Arginine biosynthesis/Alanine, aspartate and glutamate metabolism |
| 198 | A0A1D6I8F0 | Zm00001d021126 | Ankyrin repeat domain-containing protein 2A | 26.17 | 3 | 0.81 | 2.47E-03 | Down |  |
| 199 | A0A1D6GJ34 | Zm00001d013415 | Receptor-like kinase TMK2 | 13.72 | 9 | 0.81 | 1.48E-02 | Down |  |
| 200 | A0A1D6LL20 | Zm00001d036161 | Coatomer subunit beta' | 18 | 7 | 0.81 | 4.48E-03 | Down |  |
| 201 | B4FI21 | Zm00001d053003 | ARM repeat superfamily protein | 43.55 | 4 | 0.8 | 2.83E-02 | Down |  |
| 202 | A8DMN5 | Zm00001d003398 | Protein FLOURY 1 | 30.79 | 9 | 0.8 | 1.18E-02 | Down |  |
| 203 | K7UDG5 | Zm00001d053196 | Proline--tRNA ligase cytoplasmic | 62.2 | 8 | 0.8 | 2.06E-03 | Down | Aminoacyl-tRNA biosynthesis |
| 204 | A4GWX8 | Zm00001d026514 | Inositol-pentakisphosphate 2-kinase | 6.14 | 3 | 0.8 | 4.20E-02 | Down | Inositol phosphate metabolism/Phosphatidylinositol signaling system |
| 205 | A0A1D6LIW0 | Zm00001d035812 | Translocase of chloroplast 159 chloroplastic | 17.34 | 3 | 0.8 | 7.37E-04 | Down |  |
| 206 | C0P3Z8 | Zm00001d020003 | Ubiquitin-conjugating enzyme E2 variant 1C | 62.84 | 4 | 0.8 | 1.36E-02 | Down |  |
| 207 | A0A1D6L1Y6 | Zm00001d033745 | Putative inactive receptor kinase | 12.1 | 7 | 0.8 | 1.48E-03 | Down |  |
| 208 | A0A1D6PSC4 | Zm00001d049099 | Protein disulfide-isomerase | 76.96 | 23 | 0.8 | 1.89E-03 | Down | Protein processing in endoplasmic reticulum |
| 209 | A0A096R382 | Zm00001d034074 | Alpha-1,4 glucan phosphorylase | 57.42 | 44 | 0.8 | 2.68E-03 | Down | Starch and sucrose metabolism |
| 210 | A0A1D6GWP6 | Zm00001d014844 | 14-alpha-glucan-branching enzyme 2-2 chloroplastic/amyloplastic | 34.62 | 22 | 0.8 | 2.08E-03 | Down | Starch and sucrose metabolism |
| 211 | B6TJW0 | Zm00001d032781 | Uncharacterized protein | 50 | 13 | 0.8 | 3.80E-03 | Down |  |
| 212 | A0A1D6FJ37 | Zm00001d009359 | Ubiquitin fusion degradation UFD1 family protein | 3.26 | 2 | 0.8 | 2.91E-03 | Down |  |
| 213 | Q946V2 | Zm00001d035700 | Legumin 1 | 52.38 | 2 | 0.8 | 1.70E-02 | Down |  |
| 214 | A0A1D6M201 | Zm00001d037939 | Uncharacterized protein | 9.61 | 5 | 0.8 | 4.44E-02 | Down | Fatty acid degradation/alpha-Linolenic acid metabolism |
| 215 | B4FQ28 | Zm00001d009572 | Ras-related protein RABA1f | 46.76 | 5 | 0.8 | 1.27E-03 | Down |  |
| 216 | A0A1D6P4L6 | Zm00001d046714 | NAD(P)-binding Rossmann-fold superfamily protein | 26.46 | 3 | 0.79 | 4.70E-02 | Down |  |
| 217 | B4FLX5 | Zm00001d016435 | Glycosyltransferase | 23.2 | 8 | 0.79 | 2.51E-03 | Down |  |
| 218 | B4FQ44 | Zm00001d037498 | L-tryptophan--pyruvate aminotransferase 1 | 28.54 | 7 | 0.79 | 3.19E-03 | Down | Tryptophan metabolism |
| 219 | B4FKD5 | Zm00001d031747 | Eukaryotic translation initiation factor 6 | 29.39 | 4 | 0.79 | 2.11E-03 | Down | Ribosome biogenesis in eukaryotes |
| 220 | B4FTF3 | Zm00001d043538 | BURP domain protein RD22 | 36.84 | 11 | 0.79 | 6.47E-04 | Down |  |
| 221 | A0A1D6MLT1 | Zm00001d039918 | Cytochrome P450 family 77 protein | 9.48 | 4 | 0.79 | 4.68E-03 | Down |  |
| 222 | A0A1D6NRU4 | Zm00001d044845 | Binding | 5.14 | 8 | 0.79 | 5.06E-03 | Down |  |
| 223 | A0A1D6H1L8 | Zm00001d015399 | Choline-phosphate cytidylyltransferase 2 | 13.84 | 3 | 0.79 | 3.84E-02 | Down | Glycerophospholipid metabolism/Phosphonate and phosphinate metabolism |
| 224 | B6TVP8 | Zm00001d050430 | Stromal cell-derived factor 2 | 14.81 | 3 | 0.79 | 4.48E-03 | Down |  |
| 225 | B4FTR6 | Zm00001d011188 | 3'-5' exonuclease domain-containing protein / K homology domain-containing protein / KH domain-containing protein | 24.48 | 3 | 0.79 | 3.86E-03 | Down |  |
| 226 | B4FT23 | Zm00001d052698 | 14-3-3-like protein | 59.13 | 5 | 0.79 | 3.57E-03 | Down |  |
| 227 | A0A1Q1BN65 | Zm00001d008663 | Uncharacterized protein | 25.34 | 5 | 0.79 | 6.15E-03 | Down |  |
| 228 | A0A1D6E8H3 | Zm00001d003309 | Amine oxidase | 10.73 | 6 | 0.79 | 2.32E-03 | Down | Glycine, serine and threonine metabolism/Phenylalanine metabolism |
| 229 | B6U6K9 | Zm00001d000178 | Autophagy-related protein 3 | 16.4 | 4 | 0.79 | 1.25E-02 | Down | Autophagy - other eukaryotes |
| 230 | B4G1K7 | Zm00001d039154 | 26S proteasome regulatory subunit RPN13 | 26.54 | 5 | 0.79 | 4.01E-02 | Down | Proteasome |
| 231 | C0PCW9 | Zm00001d038059 | Uncharacterized protein | 2.69 | 2 | 0.79 | 2.05E-03 | Down |  |
| 232 | B4FAQ5 | Zm00001d009446 | VIP1 protein | 38.61 | 3 | 0.79 | 2.58E-02 | Down |  |
| 233 | B4FEC3 | Zm00001d018961 | Pyruvate kinase | 28.87 | 8 | 0.78 | 1.58E-04 | Down | Pyruvate metabolism/Purine metabolism/Glycolysis / Gluconeogenesis |
| 234 | A0A1D6FNY1 | Zm00001d010050 | Cytosolic endo-beta-N-acetylglucosaminidase 1 | 3.07 | 2 | 0.78 | 1.23E-02 | Down | Other glycan degradation |
| 235 | B6SRI4 | Zm00001d032231 | 14-3-3-like protein | 71.48 | 3 | 0.78 | 1.51E-03 | Down |  |
| 236 | B6U0H2 | Zm00001d022077 | 6-phosphogluconolactonase | 19.57 | 4 | 0.78 | 6.89E-04 | Down | Pentose phosphate pathway |
| 237 | C0PGE4 | Zm00001d031794 | Alpha-amylase | 46.03 | 14 | 0.78 | 2.63E-02 | Down | Starch and sucrose metabolism |
| 238 | B6T1V6 | Zm00001d038543 | Calmodulin | 64.43 | 2 | 0.78 | 5.94E-03 | Down | Phosphatidylinositol signaling system/Plant-pathogen interaction/MAPK signaling pathway - plant |
| 239 | A0A1D6F7X9 | Zm00001d007679 | Sister-chromatid cohesion protein 3 | 5.8 | 2 | 0.78 | 2.36E-02 | Down |  |
| 240 | A0A1D6Q0H2 | Zm00001d050225 | Thioredoxin domain-containing protein 9-like protein | 25.47 | 6 | 0.78 | 8.43E-03 | Down |  |
| 241 | A0A1D6MKP5 | Zm00001d039710 | Thioredoxin H-type | 28.99 | 2 | 0.78 | 2.12E-02 | Down |  |
| 242 | B4F9T3 | Zm00001d025893 | Uncharacterized protein | 74.92 | 17 | 0.78 | 9.61E-04 | Down |  |
| 243 | C0PE60 | Zm00001d031489 | Uncharacterized protein | 8.14 | 3 | 0.78 | 3.77E-03 | Down |  |
| 244 | B6UC34 | Zm00001d008846 | Glutaminyl-tRNA synthetase | 45.1 | 9 | 0.77 | 1.16E-03 | Down | Aminoacyl-tRNA biosynthesis |
| 245 | B6TFS9 | Zm00001d052796 | 14-3-3-like protein A | 47.49 | 6 | 0.77 | 3.82E-04 | Down |  |
| 246 | A0A1D6J3M4 | Zm00001d024998 | Acetyl-coenzyme A carboxylase1 | 18.86 | 3 | 0.77 | 8.53E-03 | Down | Propanoate metabolism/Monobactam biosynthesis/Fatty acid biosynthesis/Pyruvate metabolism |
| 247 | B6TFK5 | Zm00001d050969 | Elongation factor Tu | 36.05 | 2 | 0.77 | 2.64E-03 | Down |  |
| 248 | B6TMW7 | Zm00001d008744 | Transaminase/ transferase, transferring nitrogenous groups | 50.25 | 7 | 0.77 | 1.31E-03 | Down | Ribosome |
| 249 | A0A1D6G4W4 | Zm00001d011920 | WD repeat-containing protein domain phosphoinositide-interacting protein 3 | 12.17 | 4 | 0.77 | 9.37E-03 | Down |  |
| 250 | B6TQ36 | Zm00001d000227 | Pyruvate dehydrogenase E1 component subunit beta | 23.48 | 8 | 0.77 | 1.36E-02 | Down | Pyruvate metabolism/Glycolysis / Gluconeogenesis/Citrate cycle (TCA cycle) |
| 251 | B6TE61 | Zm00001d046713 | Dihydroflavonol-4-reductase | 29.08 | 2 | 0.77 | 4.94E-02 | Down |  |
| 252 | C0P9T6 | Zm00001d044673 | Sterol 14-demethylase | 18.78 | 8 | 0.77 | 4.42E-03 | Down | Steroid biosynthesis |
| 253 | B4FUM8 | Zm00001d022393 | Ankyrin repeat family protein | 25.49 | 2 | 0.77 | 7.34E-03 | Down |  |
| 254 | A0A1D6NT44 | Zm00001d045042 | Sucrose synthase | 60.38 | 39 | 0.77 | 8.19E-04 | Down | Starch and sucrose metabolism |
| 255 | B6UGN8 | Zm00001d029628 | Cysteine proteinase inhibitor | 60.47 | 5 | 0.77 | 4.09E-03 | Down |  |
| 256 | A0A1D6MCY7 | Zm00001d039038 | Eukaryotic translation initiation factor 3 subunit D | 13.92 | 3 | 0.77 | 2.26E-03 | Down |  |
| 257 | A0A1D6M063 | Zm00001d037734 | Serine/threonine-protein phosphatase 5 | 7.63 | 3 | 0.76 | 3.39E-03 | Down |  |
| 258 | B6SK58 | Zm00001d001772 | Harpin inducing protein | 25.5 | 3 | 0.76 | 1.73E-02 | Down |  |
| 259 | B4FVK0 | Zm00001d037538 | Arginine--tRNA ligase chloroplastic/mitochondrial | 20.36 | 3 | 0.76 | 4.17E-04 | Down | Aminoacyl-tRNA biosynthesis |
| 260 | B4FY98 | Zm00001d014734 | Arogenate dehydrogenase 1 chloroplastic | 9.72 | 3 | 0.76 | 1.61E-02 | Down | Phenylalanine, tyrosine and tryptophan biosynthesis |
| 261 | C0P7R2 | Zm00001d031071 | Albino or pale green mutant1 | 28.29 | 7 | 0.76 | 6.56E-03 | Down | Ubiquinone and other terpenoid-quinone biosynthesis |
| 262 | A0A1D6H888 | Zm00001d016496 | Polyadenylate-binding protein-interacting protein 3 | 13.56 | 4 | 0.76 | 7.97E-04 | Down |  |
| 263 | A0A1D6EAK0 | Zm00001d003654 | Protein disulfide isomerase10 | 18.34 | 2 | 0.76 | 3.99E-02 | Down |  |
| 264 | B4FCA4 | Zm00001d028143 | COP9 signalosome complex subunit 4 | 12.78 | 4 | 0.76 | 4.12E-02 | Down |  |
| 265 | B4F9U9 | Zm00001d007187 | Hydroxyproline-rich glycoprotein family protein | 46.65 | 14 | 0.76 | 4.02E-03 | Down |  |
| 266 | B6SI41 | Zm00001d030548 | Leucoanthocyanidin dioxygenase | 12.25 | 4 | 0.76 | 2.10E-02 | Down |  |
| 267 | A0A1D6L560 | Zm00001d034062 | PLASMODESMATA CALLOSE-BINDING PROTEIN 2 | 36.89 | 2 | 0.76 | 9.88E-03 | Down |  |
| 268 | A0A1D6GBP8 | Zm00001d012748 | Trehalose-6-phosphate synthase15 | 27.1 | 4 | 0.75 | 8.12E-03 | Down | Starch and sucrose metabolism |
| 269 | A0A1D6FRP1 | Zm00001d010530 | Cysteine--tRNA ligase 1 cytoplasmic | 31.27 | 3 | 0.75 | 1.44E-02 | Down | Aminoacyl-tRNA biosynthesis |
| 270 | B6T916 | Zm00001d027703 | N-acetyltransferase | 21.15 | 3 | 0.75 | 2.14E-02 | Down |  |
| 271 | C4J5B2 | Zm00001d028606 | Oxysterol-binding protein-related protein 3B | 24.95 | 7 | 0.75 | 6.65E-04 | Down |  |
| 272 | B4F9P7 | Zm00001d006179 | Rhomboid-like protein | 17.34 | 3 | 0.75 | 6.90E-03 | Down |  |
| 273 | A0A1D6MV33 | Zm00001d041214 | Plasma membrane ATPase | 32.74 | 8 | 0.75 | 1.84E-03 | Down | Oxidative phosphorylation |
| 274 | B6TUE5 | Zm00001d027976 | FIP1 | 14.29 | 3 | 0.75 | 1.18E-03 | Down |  |
| 275 | A0A1D6JW40 | Zm00001d028425 | Chloride conductance regulatory protein ICln | 45.69 | 4 | 0.75 | 2.16E-03 | Down | RNA transport |
| 276 | A0A1D6HZW0 | Zm00001d019706 | UNC-50 family protein | 25 | 2 | 0.75 | 1.15E-02 | Down |  |
| 277 | B6TB19 | Zm00001d046995 | Patellin-5 | 4.23 | 2 | 0.74 | 2.42E-02 | Down |  |
| 278 | A0A1D6P4G7 | Zm00001d046683 | Stromal processing peptidase chloroplastic | 9.75 | 6 | 0.74 | 1.99E-02 | Down |  |
| 279 | A0A1D6GPQ4 | Zm00001d014058 | Heterogeneous nuclear ribonucleoprotein 1 | 14.61 | 2 | 0.74 | 4.52E-02 | Down | mRNA surveillance pathway |
| 280 | Q9FPK7 | Zm00001d028180 | Inositol-3-phosphate synthase | 28.43 | 3 | 0.73 | 1.04E-03 | Down | Inositol phosphate metabolism |
| 281 | A0A1D6N2W9 | Zm00001d042329 | Mitochondrial import receptor subunit TOM20-4 | 45.61 | 6 | 0.72 | 2.65E-04 | Down |  |
| 282 | B4FSH1 | Zm00001d047983 | LisH and RanBPM domains containing protein | 12.24 | 2 | 0.72 | 1.09E-04 | Down |  |
| 283 | Q5TIM9 | Zm00001d051681 | Alpha-6-galactosyltransferase | 4.73 | 2 | 0.71 | 3.40E-02 | Down |  |
| 284 | A0A1D6I1V0 | Zm00001d020055 | Polyadenylate-binding protein-interacting protein 9 | 17.5 | 5 | 0.71 | 9.83E-03 | Down |  |
| 285 | Q71RX2 | Zm00001d021709 | Isopentenyl pyrophosphate isomerase | 56.96 | 7 | 0.71 | 2.75E-03 | Down | Terpenoid backbone biosynthesis |
| 286 | B4FEM2 | Zm00001d028243 | Uncharacterized protein | 7.89 | 2 | 0.7 | 8.50E-04 | Down | Starch and sucrose metabolism |
| 287 | A0A1D6GG86 | Zm00001d013147 | Uncharacterized protein | 6.54 | 2 | 0.69 | 2.45E-02 | Down |  |
| 288 | B6SGR7 | Zm00001d007897 | Ubiquitin-conjugating enzyme E2 2 | 17.76 | 2 | 0.68 | 3.18E-02 | Down | Ubiquitin mediated proteolysis |
| 289 | A0A1D6IN22 | Zm00001d022467 | Glycosyltransferase | 16.46 | 8 | 0.65 | 1.95E-03 | Down |  |

^1^ Accession, unique protein identifying number in the UniProt database; ^2^Gene ID/name, ID number of the corresponding gene of the identified differentially abundant protein as searched against the maize sequence database Gramene (<http://ensemble.gramene.org/Zea mays>); ^3^ Description, annotated biological functions based on Gene Ontology (GO) analysis; ^4^ Coverage (%), sequence coverage is calculated as the number of amino acids in the peptide fragments observed divided by the protein amino acid length; ^5^ Peptides fragments, refer to the number of matched peptide fragments generated by trypsin digestion;^6^ Fold change, is expressed as the ratio of intensities of up-regulated or down-regulated proteins between drought stress treatments and control (well-watered conditions); All the fold change figures below 0.83 represents that the proteins were down-regulated. All the figures above 1.2 means the proteins were up-regulated; ^7^ *P-*value, statistical level (using Student’s *t*-test) below <0.05, at which protein differential expression was accepted as significant;^8^ Expr., gene expression level. Up-, up-regulated; Down- , down-regulated;^9^ Pathways, metabolic Kyoto Encyclopedia of Genes and Genomes (KEGG) pathways in which the identified protein was found to be significantly enriched; ^10^ uncharacterized protein, a protein without any functional annotations ascribed to it at the present.
