# Supplementary material for: Comparative Proteomic and Morpho-Physiological Analyses of Maize Wild-Type Vp16 and Mutant vp16 Germinating Seed Responses to PEG-Induced Drought Stress
Source: Int J Mol Sci. 2019 Nov 8;20(22):5586. doi: 10.3390/ijms20225586 (PMC6888951; doi:10.3390/ijms20225586)
Supplement: Supplementary file 1 [file ijms-20-05586-s001.zip › Supplemantery Tables/Supplementary Table 4.doc]

**Supplementary Table 4.** Drought responsive DAPs of the mutant vp16 that were also differentially expressed between the vp16 and Vp16 after PEG treatment

| **№** | **Accession**^1^ | **Gene Name/ID** ^2^ | **Description**^3^ | **Cover**.(%)^4^ | **Pept.** ^5^ | **WD_MD** | | | **MC_MD** | | | **Pathways** ^8^ |
| --- | --- | --- | --- | --- | --- | --- | --- | --- | --- | --- | --- | --- |
|  |  |  |  |  |  | **Fold change**^6^ | **p Value**^7^ | **Expr.**^8^ | **Fold change**^6^ | **p Value**^7^ | **Expr.**^8^ |  |
| 1 | B6T2T4 | Zm00001d013907 | Nonspecific lipid-transfer protein | 41.57 | 3 | 1.45 | 5.45E-03 | Up | 0.79 | 2.75E-02 | Down |  |
| 2 | A0A1D6I6R9 | 103632923 | ^9^Uncharacterized protein | 20.18 | 2 | 1.45 | 2.78E-02 | Up | 0.81 | 4.38E-02 | Down |  |
| 3 | B6SP06 | Zm00001d053908 | Glycine-rich protein 2b | 16.83 | 2 | 1.27 | 2.33E-02 | Up | 0.78 | 1.63E-02 | Down |  |
| 4 | B4FF03 | Zm00001d045792 | Uncharacterized protein | 16.98 | 4 | 0.76 | 1.62E-03 | Down | 1.2 | 2.27E-02 | Up |  |
| 5 | C4JA94 | Zm00001d037401 | Uncharacterized protein | 4.79 | 2 | 0.76 | 2.94E-02 | Down | 1.29 | 4.05E-02 | Up | Proteasome |
| 6 | A0A1D6H1J8 | 10028298 | Glyceraldehyde-3-phosphate dehydrogenase | 60.58 | 5 | 0.7 | 5.56E-03 | Down | 1.29 | 2.42E-02 | Up | Carbon fixation in photosynthetic organisms |
| 7 | A0A1D6M323 | 100283907 | Ribosomal protein | 5.22 | 2 | 0.68 | 1.30E-03 | Down | 1.23 | 2.95E-03 | Up | Ribosome |

^1^ Accession, unique protein identifying number in the UniProt database; ^2^Gene ID/name, ID number of the corresponding gene of the identified differentially abundant protein as searched against the maize sequence database Gramene (<http://ensemble.gramene.org/Zea mays>); ^3^ Description, annotated biological functions based on Gene Ontology (GO) analysis; ^4^ Coverage (%), sequence coverage is calculated as the number of amino acids in the peptide fragments observed divided by the protein amino acid length; ^5^ Peptides fragments, refer to the number of matched peptide fragments generated by trypsin digestion;^6^ Fold change, is expressed as the ratio of intensities of up-regulated or down-regulated proteins between drought stress treatments and control (well-watered conditions); All the fold change figures below 0.83 represents that the proteins were down-regulated. All the figures above 1.2 means the proteins were up-regulated; ^7^ *P-*value, statistical level (using Student’s *t*-test) below <0.05, at which protein differential expression was accepted as significant;^8^ Expr., gene expression level. Up-, up-regulated; Down- , down-regulated;^9^ Pathways, metabolic Kyoto Encyclopedia of Genes and Genomes (KEGG) pathways in which the identified protein was found to be significantly enriched; ^10^ uncharacterized protein, a protein without any functional annotations ascribed to it at the present.
