# Supplementary material for: Comparative Proteomic and Morpho-Physiological Analyses of Maize Wild-Type Vp16 and Mutant vp16 Germinating Seed Responses to PEG-Induced Drought Stress
Source: Int J Mol Sci. 2019 Nov 8;20(22):5586. doi: 10.3390/ijms20225586 (PMC6888951; doi:10.3390/ijms20225586)
Supplement: Supplementary file 1 [file ijms-20-05586-s001.zip › Supplemantery Tables/Supplementary Table 5.doc]

**Supplementary Table 5.** Common (overlapping) drought-responsive germinating seeds DAPs between vp16 and Vp16

| **№** | **Accession**^1^ | **Gene Name/ID** ^2^ | **Description**^3^ | **Cover.(%)**^4^ | **Pept.** ^5^ | **WC_WD** | | | **MC_MD** | | | **Pathways** ^9^ |
| --- | --- | --- | --- | --- | --- | --- | --- | --- | --- | --- | --- | --- |
|  |  |  |  |  |  | **Fold change**^6^ | **p Value**^7^ | **Expr.**^8^ | **Fold change**^6^ | **p Value**^7^ | **Expr.**^8^ |  |
| 1 | A0A1D6FZP4 | Zm00001d011386 | Uroporphyrinogen decarboxylase | 13.9 | 3 | 0.81 | 3.49E-03 | Down | 0.82 | 6.14E-03 | Down | Porphyrin and chlorophyll metabolism |
| 2 | A0A1D6KL85 | Zm00001d031773 | HVA22-like protein | 17.39 | 2 | 0.64 | 1.98E-02 | Down | 0.64 | 1.07E-04 | Down |  |
| 3 | B4FHW2 | Zm00001d013568 | ^9^Uncharacterized protein | 18.75 | 2 | 1.4 | 3.74E-02 | Up | 0.81 | 3.18E-02 | Down |  |
| 4 | B4G231 | Zm00001d005890 | Alpha-amylase | 67.2 | 20 | 1.21 | 1.21E-02 | Up | 1.2 | 7.36E-03 | Up | Starch and sucrose metabolism |
| 5 | B6TJE9 | Zm00001d045310 | Mitogen-activated protein kinase | 20.85 | 6 | 0.82 | 2.01E-02 | Down | 0.8 | 3.10E-02 | Down | MAPK signaling pathway - plant/Plant hormone signal transduction |
| 6 | C0PK64 | Zm00001d039221 | Nucleoside diphosphate kinase | 49.38 | 2 | 0.66 | 3.73E-02 | Down | 0.75 | 3.17E-02 | Down |  |
| 7 | D2IPC3 | Zm00001d050032 | ADP-glucose pyrophosphorylase small subunit (Fragment) | 71.12 | 2 | 0.57 | 9.46E-03 | Down | 0.69 | 1.66E-03 | Down |  |

^1^ Accession, unique protein identifying number in the UniProt database; ^2^Gene ID/name, ID number of the corresponding gene of the identified differentially abundant protein as searched against the maize sequence database Gramene (<http://ensemble.gramene.org/Zea mays>); ^3^ Description, annotated biological functions based on Gene Ontology (GO) analysis; ^4^ Coverage (%), sequence coverage is calculated as the number of amino acids in the peptide fragments observed divided by the protein amino acid length; ^5^ Peptides fragments, refer to the number of matched peptide fragments generated by trypsin digestion;^6^ Fold change, is expressed as the ratio of intensities of up-regulated or down-regulated proteins between drought stress treatments and control (well-watered conditions); All the fold change figures below 0.83 represents that the proteins were down-regulated. All the figures above 1.2 means the proteins were up-regulated; ^7^ *P-*value, statistical level (using Student’s *t*-test) below <0.05, at which protein differential expression was accepted as significant;^8^ Expr., gene expression level. Up-, up-regulated; Down- , down-regulated;^9^ Pathways, metabolic Kyoto Encyclopedia of Genes and Genomes (KEGG) pathways in which the identified protein was found to be significantly enriched; ^10^ uncharacterized protein, a protein without any functional annotations ascribed to it at the present.
