# Supplementary material for: Comparative Proteomic and Morpho-Physiological Analyses of Maize Wild-Type Vp16 and Mutant vp16 Germinating Seed Responses to PEG-Induced Drought Stress
Source: Int J Mol Sci. 2019 Nov 8;20(22):5586. doi: 10.3390/ijms20225586 (PMC6888951; doi:10.3390/ijms20225586)
Supplement: Supplementary file 1 [file ijms-20-05586-s001.zip › Supplemantery Tables/Supplementary Table 6.doc]

**Supplementary Table 6. Drought-responsive maize germinating seed proteins observed specifically in WC_WD comparison**

| **№** | **Accession^1^** | **Gene Name/ID ^2^** | **Description^3^** | **Cover.(%)^4^** | **Pept. ^5^** | **Fold Change^6^** | **P-value^7^** | **Expr.^8^** | **Pathways^9^** |
| --- | --- | --- | --- | --- | --- | --- | --- | --- | --- |
|  |  |  |  |  |  |  |  |  |  |
| 1 | C0PBZ6 | Zm00001d005971 | Non-specific lipid-transfer protein | 24.47 | 5 | 1.70 | 3.21E-02 | Up |  |
| 2 | A0A096TAY4 | Zm00001d019163 | Stachyose synthase | 4.5 | 3 | 1.37 | 1.70E-02 | Up | Galactose metabolism |
| 3 | A0A1D6PJL0 | Zm00001d048358 | Aconitate hydratase | 28.72 | 2 | 1.34 | 3.27E-02 | Up |  |
| 4 | C0HHF9 | Zm00001d002853 | ^10^Uncharacterized protein | 7.94 | 4 | 1.30 | 2.40E-02 | Up |  |
| 5 | A0A1D6HPC4 | Zm00001d018479 | NADH dehydrogenase [ubiquinone] 1 alpha subcomplex subunit 9 mitochondrial | 35.38 | 10 | 1.27 | 2.92E-02 | Up | Oxidative phosphorylation |
| 6 | B6SKA0 | Zm00001d012675 | Glutathione S-transferase 1 | 51.87 | 9 | 1.27 | 3.07E-02 | Up | Glutathione metabolism |
| 7 | A0A1D6JT22 | Zm00001d028207 | ATP synthase subunit gamma | 40.83 | 3 | 1.25 | 1.17E-02 | Up | Oxidative phosphorylation |
| 8 | B4FMB1 | Zm00001d037018 | DUF538 family protein | 58.39 | 7 | 1.24 | 3.23E-02 | Up |  |
| 9 | B6T8U6 | Zm00001d030618 | Cytochrome c oxidase polypeptide | 24.19 | 2 | 1.24 | 3.03E-02 | Up |  |
| 10 | Q8W0Q2 | Zm00001d038841 | Putative aldehyde dehydrogenase MIS1 | 40.98 | 18 | 1.24 | 2.20E-02 | Up | Arginine and proline metabolism |
| 11 | A0A1D6HFD0 | Zm00001d017522 | Tripeptidyl-peptidase 2 | 10.81 | 10 | 1.23 | 2.93E-02 | Up |  |
| 12 | A0A1D6GBB9 | Zm00001d012710 | SGS domain-containing protein | 31.79 | 4 | 1.23 | 4.30E-02 | Up |  |
| 13 | A0A1D6I5L0 | Zm00001d020669 | Formate tetrahyrofolate ligase | 36.56 | 22 | 1.23 | 3.07E-02 | Up | One carbon pool by folate |
| 14 | B6TPP7 | Zm00001d038904 | Threonine synthase | 27.01 | 7 | 1.22 | 4.59E-02 | Up | Glycine, serine and threonine metabolism |
| 15 | B6UH55 | Zm00001d053765 | Vacuolar ATP synthase catalytic subunit A | 64.41 | 8 | 1.22 | 1.68E-02 | Up | Phagosome |
| 16 | A0A1D6H483 | Zm00001d015837 | Outer envelope protein 80 chloroplastic | 3.04 | 2 | 1.21 | 7.46E-03 | Up |  |
| 17 | A0A1D6MNJ0 | Zm00001d040163 | Deoxy xylulose reductoisomerase1 | 27.75 | 11 | 1.21 | 1.88E-02 | Up | Terpenoid backbone biosynthesis |
| 18 | B6U0C2 | Zm00001d008287 | Phenazine biosynthesis PhzC/PhzF protein | 35.81 | 8 | 1.21 | 1.07E-02 | Up |  |
| 19 | B6TEP5 | Zm00001d020004 | Triose phosphate/phosphate translocator, non-green plastid,chloroplast | 11.84 | 5 | 1.21 | 4.13E-02 | Up |  |
| 20 | A0A1D6LJU9 | Zm00001d035957 | Plant/F18O14-17 protein | 2.84 | 2 | 1.21 | 1.44E-02 | Up |  |
| 21 | B4FE88 | Zm00001d021309 | Uncharacterized protein | 11.03 | 2 | 1.20 | 2.20E-02 | Up | Basal transcription factors |
| 22 | A0A1D6FM39 | Zm00001d009795 | Cellulose synthase1 | 3.09 | 2 | 1.20 | 3.80E-02 | Up |  |
| 23 | B6T9C2 | Zm00001d028359 | Transmembrane 9 superfamily member | 10.46 | 2 | 0.83 | 5.64E-03 | Down |  |
| 24 | B4FLZ5 | Zm00001d006904 | Multifunctional methyltransferase subunit TRM112-like protein | 30.53 | 3 | 0.83 | 1.45E-03 | Down |  |
| 25 | B4G0F2 | Zm00001d019311 | IAA-amino acid hydrolase ILR1 | 35.97 | 10 | 0.83 | 3.46E-03 | Down |  |
| 26 | A0A1D6L9R2 | Zm00001d034640 | NEDD8-activating enzyme E1 regulatory subunit | 21.17 | 8 | 0.82 | 3.38E-02 | Down |  |
| 27 | C4J2G4 | Zm00001d021289 | Late embryogenesis abundant (LEA) hydroxyproline-rich glycoprotein family | 18.26 | 3 | 0.82 | 4.66E-02 | Down |  |
| 28 | B6SPL7 | Zm00001d031132 | Copper chaperone | 83.33 | 5 | 0.81 | 3.78E-02 | Down |  |
| 29 | C0HHR4 | Zm00001d032480 | Actin-7 | 63.4 | 3 | 0.81 | 2.96E-02 | Down |  |
| 30 | A0A1D6EVX9 | Zm00001d006410 | COP9 signalosome complex subunit 7 | 12.7 | 2 | 0.80 | 5.15E-03 | Down |  |
| 31 | B6TGG7 | Zm00001d037108 | 3-oxoacyl-[acyl-carrier-protein] synthase | 43.17 | 4 | 0.80 | 1.73E-02 | Down | Fatty acid biosynthesis |
| 32 | B4FRS6 | Zm00001d011650 | VIP1 protein | 8.75 | 2 | 0.79 | 3.49E-02 | Down |  |
| 33 | A0A1D6L913 | Zm00001d034590 | Mitochondrial Rho GTPase | 38.33 | 3 | 0.77 | 8.85E-03 | Down |  |
| 34 | C0PD88 | Zm00001d043350 | Putative indole-3-acetic acid-amido synthetase | 34.11 | 11 | 0.77 | 1.54E-02 | Down | Plant hormone signal transduction |
| 35 | C0PNC3 | Zm00001d019155 | Uncharacterized protein | 7.58 | 2 | 0.73 | 2.86E-04 | Down |  |
| 36 | Q00LN8 | Zm00001d005793 | 16 kDa gamma zein | 18.89 | 2 | 0.72 | 1.29E-02 | Down |  |
| 37 | B4FAB1 | Zm00001d049649 | Succinate dehydrogenase [ubiquinone] iron-sulfur subunit, mitochondrial | 30.69 | 2 | 0.69 | 4.10E-02 | Down | Oxidative phosphorylation |
| 38 | A0A1D6E7I8 | Zm00001d003207 | Putative E3 ubiquitin-protein ligase ARI8 | 7.96 | 2 | 0.68 | 4.64E-02 | Down |  |
| 39 | C4JBV8 | Zm00001d002847 | (+)-neomenthol dehydrogenase | 29.62 | 8 | 0.64 | 2.02E-02 | Down | Monoterpenoid biosynthesis |
| 40 | K7U234 | Zm00001d024960 | Bowman-Birk type trypsin inhibitor | 35.05 | 2 | 0.62 | 1.42E-02 | Down |  |
| 41 | B6SK40 | Zm00001d012017 | Mitochondrial import receptor subunit TOM20 | 15.35 | 2 | 0.57 | 3.39E-02 | Down |  |

^1^ Accession, unique protein identifying number in the UniProt database; ^2^Gene ID/name, ID number of the corresponding gene of the identified differentially abundant protein as searched against the maize sequence database Gramene (<http://ensemble.gramene.org/Zea mays>); ^3^ Description, annotated biological functions based on Gene Ontology (GO) analysis; ^4^ Coverage (%), sequence coverage is calculated as the number of amino acids in the peptide fragments observed divided by the protein amino acid length; ^5^ Peptides fragments, refer to the number of matched peptide fragments generated by trypsin digestion;^6^ Fold change, is expressed as the ratio of intensities of up-regulated or down-regulated proteins between drought stress treatments and control (well-watered conditions); All the fold change figures below 0.83 represents that the proteins were down-regulated. All the figures above 1.2 means the proteins were up-regulated; ^7^ *P-*value, statistical level (using Student’s *t*-test) below <0.05, at which protein differential expression was accepted as significant;^8^ Expr., gene expression level. Up-, up-regulated; Down- , down-regulated;^9^ Pathways, metabolic Kyoto Encyclopedia of Genes and Genomes (KEGG) pathways in which the identified protein was found to be significantly enriched; ^10^ uncharacterized protein, a protein without any functional annotations ascribed to it at the present.
