# Supplementary material for: Comparative Proteomic and Morpho-Physiological Analyses of Maize Wild-Type Vp16 and Mutant vp16 Germinating Seed Responses to PEG-Induced Drought Stress
Source: Int J Mol Sci. 2019 Nov 8;20(22):5586. doi: 10.3390/ijms20225586 (PMC6888951; doi:10.3390/ijms20225586)
Supplement: Supplementary file 1 [file ijms-20-05586-s001.zip › Supplemantery Tables/Supplementary Table 9.doc]

**Supplementary Table 9. Gene specific primers used for qRT-PCR analysis**

| Assession^1^ | Description^2^ | Gene ID/Name^3^ | F(5'-3')^4^ | R(5'-3')^5^ |
| --- | --- | --- | --- | --- |
| A0A1D6KL85 | HVA22-like protein | Zm00001d031773 | CCTGCCCTACCGAATCTTT | GACCAGGACTTGCCCATCT |
| D2IPC3 | ADP-glucose pyrophosphorylase small subunit | Zm00001d050032 | TTCCCTCAACCGTCACCTCTC | TGCTCCTCAAACAACCACAAG |
| A0A1D6MXK3 | Jasmonate-induced protein | Zm00001d041663 | ACAAAAAACTCAACAAG | TACATAGAGGAAGAAGC |
| B4FRS8 | Germin-like protein subfamily T member 1 | 100272932 | TGGGCGTGTCCATGAACCG | AGCCGAACAGCGTGAGGGG |
| B6TFB6 | Stress responsive protein | 100283176 | CAGCCCTACTACTGAACCA | ATACACTCCGAGCAACTTT |
| B6T3V1 | Peroxidase | 100282124 | CAAGGAGACCAGGATGGCGGCA | CCAGGGCGGTGGAATCACGAGC |
| O82087 | Pathogenesis related protein-5 | PR-5 | GGCAGCCAGGACTTCTAC | GGCAGCCAGGACTTCTAC |
| B6U7D8 | Cinnamyl alcohol dehydrogenase | Zm00001d015618 | CGTCAAGGTAGCCAAGG | CGTCAAGGTAGCCAAGG |
| Q9FQA9 | Glutathione S-transferase GST 30 | 541841 | AACGAGTCCCAGGTCATC | GAACAGCATCCCCAGCCA |
| B4FGG7 | Calcium ion binding protein | 100193836 | GTTGATGCGTTTGTCTTCTT | TGTCACCATTCCGTTCTTGT |
| B4F848 | 20 kDa chaperonin chloroplastic | 100191248 | AAGCCGAAGACAAAACTCCT | ATCCGACGCTCTCAACACAA |
| B7ZEQ0 | Small heat-shock protein | hsp18 | CGCAGGAGAACAGGGACAACA | GGCGACATCGGGTCAACTAAT |
| B4G024 | Malate dehydrogenase | 100193491 | GGATGACAAGGGATGATT | ACGGTAGAGTTGACAGGA |
| B6SLU3 | Aldehyde dehydrogenase | 100280779 | AGTTCCGTAACAGTGGACAGAC | ACATTCCCTACTACAGTTGGCT |
| B8QWQ8 | Pathogenesis-related maize seed protein | PRms | CACCCTTCCTACTCTGA | GTTTCTCGTCTACCCAC |
| Q9FQA3 | Glutathione transferase GST 23 | Zm00001d020780 | CCAAGAAGGTGCCCGTGCT | GCGTCCCCGCCGAAGAACT |
| B4G231 | Alpha-amylase | Zm00001d005890 | AAGGGCGTCCAGTGCGT | AGCCGTCGAAGCCGAGG |
| A0A1D6JTN4 | Glutamine synthetase | Zm00001d028260 | CAAAGCGAGGACGGTGAA | GTCGGGGTGGCTGAAAAC |
| A0A1D6K1T6 | 23-bisphosphoglycerate-independent phosphoglycerate mutase 1 | 103634611 | ACATCCAGCAGTCCTTCCA | CAGACGCTATTCTCGCATC |
| A0A1D6H1J8 | Glyceraldehyde-3-phosphate dehydrogenase | 100282981 | GTGGGGAGAAGACAAAG | CGACACGAATAGAACCT |
| B6TX09 | Fructose-1,6-bisphosphatase, cytosolic | 100284739 | GAAGTATGGTTGCTGAT | GCTGTTTGACATTGAAG |
| B4FT23 | 14-3-3-like protein | 100283140 | TAACCTTCTATCTGTTGCCTAC | TAACCTTCTATCTGTTGCCTAC |
| K7UY19 | Fructose-bisphosphate aldolase | 100502020 | TCCAACAATGAATCGTGGTGCC | CTGCTTCCTTCGCTGCTAATGC |
| B6SQM6 | Pathogenesis-related protein 10 | 100280981 | CGGCAGCGTTACCACCATG | AGCACCTCCGTCCTCACCA |
| B4FVH1 | Malate dehydrogenase | Zm00001d009640 | GCCACTCCTGCAAGCAATTC | CCGGAACTCCATTCAGACCC |
| B6T8C2 | Histone H2A | Zm00001d006547 | GCGTTATGCCCAACATCCAC | TGGGCAGCTTCAGTTGTCAT |
| A0A1D6I7Y4 | Peptidylprolyl isomerase | 100282031 | GTCCTGCCGCTGTCTGTTAT | ACACGTCCCCGGATTTCTTG |
| K7VPB0 | Pentatricopeptide repeat-containing protein mitochondrial | 103636586 | TCGACTCACAGAAGCCGTTC | ATGGAGGACTTGTGCTTCGG |

^1^ Accession, unique protein identifying number in the UniProt database; ^2^ Description, annotated biological functions based on Gene Ontology (GO) analysis; ^3^Gene ID/name, ID number of the corresponding gene of the identified differentially abundant protein as searched against the maize sequence database Gramene (<http://ensemble.gramene.org/Zea mays>); ^4^F, forward primer; ^5^R, reverse primer.
